# Application of 7-azaisatins in enantioselective Morita–Baylis–Hillman reaction

**DOI:** 10.3762/bjoc.12.33

**Published:** 2016-02-18

**Authors:** Qing He, Gu Zhan, Wei Du, Ying-Chun Chen

**Affiliations:** 1 Key Laboratory of Drug-Targeting and Drug Delivery System of the Ministry of Education, West China School of Pharmacy, Sichuan University, Chengdu 610041, China

**Keywords:** 7-azaisatins, β-isocupreidine, bifunctional catalysis, maleimide, MBH reaction

## Abstract

7-Azaisatin and 7-azaoxindole skeletons are valuable building blocks in diverse biologically active substances. Here 7-azaisatins turned out to be more efficient electrophiles than the analogous isatins in the enantioselective Morita–Baylis–Hillman (MBH) reactions with maleimides using a bifunctional tertiary amine, β-isocupreidine (β-ICD), as the catalyst. This route allows a convenient approach to access multifunctional 3-hydroxy-7-aza-2-oxindoles with high enantiopurity (up to 94% ee). Other types of activated alkenes, such as acrylates and acrolein, could also be efficiently utilized.

## Introduction

The asymmetric Morita–Baylis–Hillman (MBH) reaction is one of the most powerful synthetic methods in organic chemistry, as it directly constructs carbon–carbon bonds in an atom-economical manner and provides densely functionalized molecules [1–4]. In particular, the direct formation of stereogenic quaternary carbon centers by enantioselective MBH reactions has been a fascinating and challenging area, because the compatible electrophiles are always limited to aldehydes or derivatives thereof. Since the first elegant work on the enantioselective MBH reaction between isatins and acrolein catalyzed by β-isocupreidine (β-ICD) was reported by the Zhou group [5], isatin derivatives, as highly activated electrophiles, have been utilized by other groups for similar transformations with acrylates or acrylamides, affording the 3-hydroxyoxindole derivatives with moderate to excellent stereocontrol [6–12]. Maleimides are also good nucleophilic precursors in the MBH reactions and in 2013, Chimni developed an asymmetric MBH reaction of isatins and maleimides with excellent enantioselectivity [13]. Later, the same group expanded this strategy to isatin-derived ketimines under the identical catalytic conditions [14]. Remarkably, these reactions were usually promoted by bifunctional catalysts, such as β-ICD, whose C6’-OH group served as a H-bond donor to facilitate the proton-transfer step and to stabilize the transition state in MBH reactions [11–14]. Nevertheless, all these cases suffered from low reactivity and long reaction times were always required (usually > 48 h) for better conversions.

**Figure 1:**
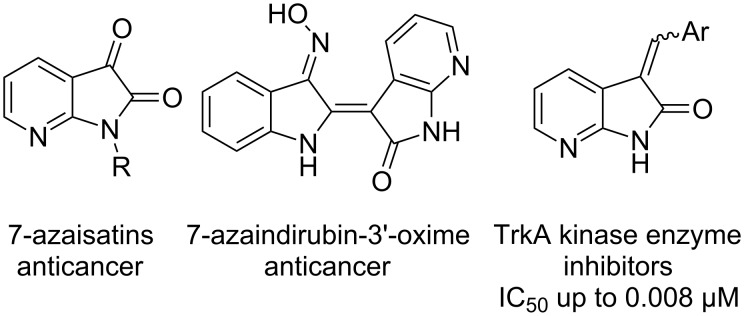
Bioactive 7-azaisatins and their derivatives.

7-Azaisatins bearing an additional nitrogen atom at the 7-position of the 2-oxindole scaffold might be better electrophiles than isatins owing to the electron-withdrawing effect of the pyridine motif. More importantly, a number of 7-azaisatins and 7-azaoxindoles were shown to exhibit significant anticancer or TrkA kinase inhibitory activities (Figure 1) [15–17]. This would render their derivatives as pharmaceutically interesting compounds with high potential. In addition, 7-azaisatins were also applied in asymmetric synthesis [18–19]. Therefore, with our continuing interest in the catalytic application of bifunctional β-ICD [20–23], herein we report the enantioselective MBH reaction of 7-azaisatins with maleimides. A series of 3-hydroxy-7-aza-2-oxindoles have been synthesized in good to excellent yields and with moderate to high enantioselectivity in a shorter time (for most cases, the reaction time is 24 h).

## Results and Discussion

The enantioselective MBH reaction was first investigated with *N*-methyl-7-azaisatin (**1a**) and *N*-phenylmaleimide (**2a**) in toluene catalyzed by β-ICD. To our delight, the desired product **3a** was obtained in 45% yield and with excellent enantioselectivity at 50 °C after 72 h (Table 1, entry 1). The reaction could be accelerated significantly by increasing the amounts of **2a** (Table 1, entries 2–5), and almost a quantitative yield could be obtained after 24 h by employing 6 equiv of **2a** (Table 1, entry 5). Other solvents, such as MeCN, THF and CHCl_3_, were also tested but provided inferior results (Table 1, entries 6–8). The attempt to improve the enantioselectivity by lowering the temperature failed (Table 1, entry 9), and the enantiocontrol was diminished at 70 °C (Table 1, entry 10). On the other hand, when α-isocupreine (α-IC) [24–25] was employed as the catalyst instead of β-ICD, unfortunately no desired product was obtained even after 120 h (Table 1, entry 11). Finally, we compared the reaction of 7-azaisatin **1a** and maleimide **2a** under the catalytic conditions developed by Chimni [13]. The reaction proceeded slowly, but a high yield with an outstanding ee value could be obtained after 72 h (Table 1, entry 12).

**Table 1:** Screening conditions of the enantioselective MBH reaction of **1a** and **2a**.

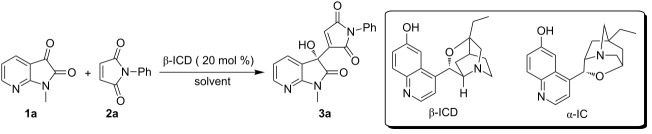						

Entry^a^	**2a** (equiv)	Solvent	Temperature (°C)	Time (h)	Yield (%)^b^	ee (%)^c^

1	1.5	toluene	50	72	45	91
2	3.0	toluene	50	72	66	92
3	4.0	toluene	50	48	83	92
4	5.0	toluene	50	48	89	91
5	6.0	toluene	50	24	98	94
6	6.0	MeCN	50	24	98	86
7	6.0	THF	50	24	98	88
8	6.0	CHCl_3_	50	96	90	91
9	6.0	toluene	rt	48	91	91
10	6.0	toluene	70	24	85	88
11^d^	6.0	toluene	50	>120	–	–
12	2.0	CHCl_3_	rt	72	90	95

^a^Unless noted otherwise, reactions were performed with **1a** (0.05 mmol), **2a** and catalyst (0.01 mmol) in solvent (0.5 mL). ^b^Isolated yield. ^c^Determined by HPLC analysis on a chiral stationary phase. ^d^α-IC was used as the catalyst.

With the optimal conditions in hand, next, the substrate scope of the MBH reaction was studied under the catalysis of β-ICD (Table 2). At first, a variety of N-substituted maleimides **2** were explored in the reaction with 7-azaisatin **1a** in toluene. Maleimides bearing an electron-rich N-aryl ring generally afforded the corresponding 3-hydroxy-7-aza-2-oxindoles in high yields and with excellent enantioselectivity (Table 2, entries 2–4) while good results were obtained in a mixture of THF and DCM for a maleimide with an electron-deficient N-aryl group because of better solubility (Table 2, entry 5). In addition, N-alkylated maleimides provided the desired products in good yields (Table 2, entries 6–9), while only moderate enantioselectivity was observed for products **3h** and **3i** (Table 2, entries 8 and 9). 7-Azaisatins with different N-protecting groups were also applied to the MBH reaction with *N*-phenylmaleimide (**2a**), including methoxymethyl (MOM), benzyl (Bn) and 4-chlorophenyl substituents. All of them showed a lower reactivity and enantioselectivity than that of the methyl-substituted one, and better results were generally obtained in a mixture of THF and DCM (Table 2, entries 10–12). The C5-phenyl substituted or halogenated 7-azaisatins could be smoothly applied, although a longer reaction time was required for the halogenated substrates (Table 2, entries 13–15).

**Table 2:** Substrate scope of the enantioselective MBH reaction.

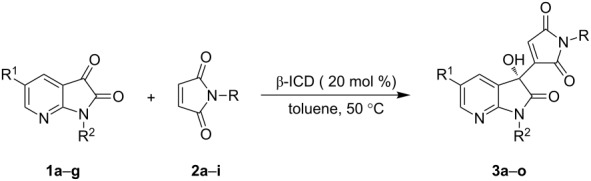						

Entry^a^	R^1^	R^2^	R	Time (h)	Yield (%)^b^	ee (%)^c^

1	H	**1a**, Me	**2a**, Ph	24	**3a**, 98	94
2	H	**1a**,Me	**2b**, 4-MePh	24	**3b**, 87	90
3	H	**1a**, Me	**2c**, 4-MeOPh	24	**3c**, 88	92
4	H	**1a**, Me	**2e**, 2,4,6-MePh	24	**3e**, 90	79
5^d^	H	**1a**, Me	**2d**, 4-ClPh	24	**3d**, 87	92
6	H	**1a**, Me	**2f**, Me	24	**3f**, 84	89
7	H	**1a**, Me	**2g**, Bn	24	**3g**, 86	89
8	H	**1a**, Me	**2h**, *n*-Bu	24	**3h**, 86	66
9	H	**1a**, Me	**2i**, cyclohexyl	24	**3i**, 84	61
10^d^	H	**1b**, MOM	**2a**, Ph	24	**3j**, 92	91
11^d^	H	**1c**, Bn	**2a**, Ph	48	**3k**, 93	87
12^d^	H	**1d**, 4-ClPh	**2a**, Ph	96	**3l**, 37	71
13^d^	Ph	**1e**, Me	**2a**, Ph	24	**3m**, 88	92
14	Cl	**1f**, Me	**2a**, Ph	48	**3n**, 81	85
15	Br	**1g**, Me	**2a**, Ph	48	**3o**, 82	88

^a^Unless otherwise noted, all reactions were performed with **1** (0.1 mmol), **2** (0.6 mmol) and β-ICD (0.02 mmol) in toluene (1.0 mL) at 50 °C. ^b^Isolated yield. ^c^Determined by HPLC analysis on a chiral stationary phase. The absolute configuration of the chiral products was assigned by analogy to Chimni’s work [13]. ^d^In a mixture of THF and DCM (0.5/0.5 mL).

**Scheme 1:**
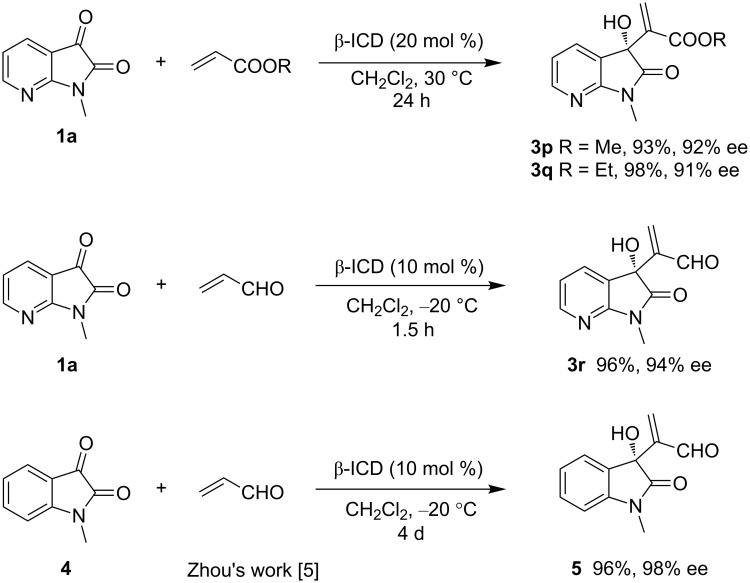
Further exploration with 7-azaisatin **1a** and comparison with the previous work by Zhou [5].

To further assess the high electrophilicity of 7-azaisatins in MBH reactions, more activated alkenes were explored with **1a**. Methyl and ethyl acrylates afforded products **3p** and **3q**, respectively, with comparable results to Wu’s work [7], albeit in a shorter time (24 h vs 2–3 d). Notably, the MBH reaction of acrolein and 7-azaisatin **1a** was carried out under the same conditions as described in Zhou’s work [5], providing the highly enantio-enriched product **3r** in an excellent yield after 1.5 h. The reaction with **1a** was much more efficient, as it took 4 d to afford the product **5** in a high yield by using *N*-methylisatin (**4**) as the substrate (Scheme 1).

## Conclusion

In summary, we have developed an efficient and enantioselective Morita–Baylis–Hillman reaction between 7-azaisatins and maleimides and other activated alkenes in the presence of the bifunctional catalyst β-ICD. 7-Azaisatins were proven to be better electrophiles than the analogous isatins, and furnished a convenient protocol to enantio-enriched multifunctional 3-hydroxy-7-aza-2-oxindoles. Such substances might be further applied in organic synthesis for potential biological and pharmaceutical studies in the future.

## Experimental

**General procedure for the synthesis of 3-hydroxy-7-aza-2-oxindoles:** A solution of N-protected 7-azaisatin **1** (0.1 mmol), N-substituted maleimide **2** (0.6 mmol) and β-ICD (20 mol %) was stirred in dry toluene (1.0 mL) at 50 °C. The progress of the reaction was monitored by TLC. After completion, the MBH reaction product **3** was purified by flash chromatography on silica gel using petroleum ether/EtOAc 6:1–3:1 as the eluent.

## Supporting Information

File 1 Detailed experimental procedures and analytical data for the compounds.
