# Supplementary material for: Application of 7-azaisatins in enantioselective Morita–Baylis–Hillman reaction
Source: Beilstein J Org Chem. 2016 Feb 18;12:309–13. doi: 10.3762/bjoc.12.33 (PMC4778495; doi:10.3762/bjoc.12.33)
Supplement: File 1 — Detailed experimental procedures and analytical data for the compounds. [file Beilstein_J_Org_Chem-12-309-s001.pdf]

Supporting Information

for

Application of 7-azaisatins in enantioselective Morita–Baylis–Hillman reaction

Qing He, Gu Zhan, Wei Du and Ying-Chun Chen*

Address: Key Laboratory of Drug-Targeting and Drug Delivery System of the Ministry of Education, West China School of Pharmacy, Sichuan University, Chengdu 610041, China

Email: Ying-Chun Chen* - ycchen@scu.edu.cn

* Corresponding author

Full experimental details and analytical data

1. General methods S2
2. Preparation of N-protected 7-azaisatins **1a**–**d** S2
3. Preparation of N-protected 7-azaisatin **1e** S4
4. Preparation of N-protected 7-azaisatins **1f** and **1g** S4
5. Preparation of *N*-protected 7-azaisatin **1h** S5
6. Preparation of N-substituted maleimides **2a**–**i** S5
7. General procedure for MBH reaction S5
8. References S13
9. NMR spectra and HPLC chromatograms S15

1. General methods

NMR data were obtained for ^{1}H at 400 MHz and for ^{13}C at 100 MHz or 150 MHz. Chemical shifts were given in parts per million (δ) from tetramethylsilane with the residual solvent resonance as the internal standard in $CDCl_3$ solution. In all cases, the enantiomeric ratio was determined by HPLC analysis on a chiral column, using a Daicel Chiralpak IC Column (250 x 4.6 mm), Chiralpak ID Column (250 x 4.6 mm), Chiralpak IE Column (250 x 4.6 mm), Chiralpak AD Column (250 x 4.6 mm) or Chiralpak AS Column (250 x 4.6 mm). UV detection was monitored at 254 nm. Optical rotation data were examined in $CHCl_3$ solution at 20 °C. Column chromatography was performed on silica gel (200–300 mesh) eluting with ethyl acetate and petroleum ether. TLC was performed on glass-backed silica plates. UV light and I_2 were used to visualize the products. All commercially available chemicals were used without purification unless otherwise noted. THF, ethyl acetate, petroleum ether, methylene chloride (CH_2Cl_2), and toluene were distilled before use. Cinchona alkaloids catalysts β-ICD and α-IC were prepared according to the literature procedures [1,2].

2. Preparation of N-protected 7-azaisatins 1a–d

The preparation of the known compound **1a**: A dried round-bottomed flask equipped with a magnetic stirring bar was charged with 7-azaindole (2 g, 16.95 mmol) and DMF (10 mL) under a nitrogen atmosphere. The mixture was cooled to 0 °C, NaH (1.2 equiv) was added and stirring continued for 1 h. Then, methyl iodide (1.1 equiv) was added and the mixture was stirred for another 1 h. Afterwards the reaction was quenched with ice cold water (100 mL) and extracted with ethyl acetate (3 x 100 mL). The combined organic layers were dried over Na_2SO_4 and the solvent was removed under reduced pressure to give *N*-methyl-7-azaindoles in a quantitative yield [3].

PCC (5.37 g, 25 mmol) was ground with silica gel (5.37 g, 70–230 mesh) and transferred to a 250 mL round-bottomed flask containing DCE (40 mL). To the orange suspension was added a solution of *N*-methyl-7-azaindole (1.32 g, 10 mmol) in DCE (5 mL) while stirring at room temperature. Then, $AlCl_3$ (15 wt %, 1.3 mol % with respect to *N*-methyl-7-azaindole) was added and the mixture was stirred at 80 °C. The progress of the reaction was monitored by TLC. After completion, the solvent was removed under reduced pressure and the black solid was treated with 50 mL of *n*-hexane/ethyl acetate (4:1) and filtered under suction through a sintered funnel layered with silica gel (5 cm, 70–230 mesh). The filtrate was evaporated to furnish **1a** as a yellow solid (1.29 g, 80%). In addition, **1c** was prepared according to the literature procedure [4]. A similar procedure was utilized for the preparation of **1b** and **1d**.

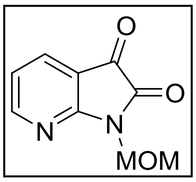

1b: Yellow solid, ^1H NMR (400 MHz, $CDCl_3$) δ 8.54 (d, *J* = 4.4 Hz, 1H), 7.91 (d, *J* = 7.2 Hz, 1H), 7.17 (dd, *J* = 7.2 Hz, *J* = 5.2 Hz, 1H), 5.29 (s, 2H), 3.48 (s, 3H) ppm; ^{13}C NMR (100 MHz, $CDCl_3$) δ 181.3, 163.2, 158.4, 156.3, 133.4, 120.2, 111.8, 70.0, 57.7 ppm.

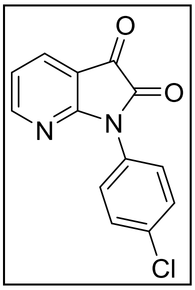

1d: Yellow solid, ^1H NMR (400 MHz, $CDCl_3$) δ 8.47 (d, *J* = 4.8 Hz, 1H), 7.97 (d, *J* = 7.6 Hz, 1H), 7.52 (s, 4H), 7.20 (dd, *J* = 7.2 Hz, *J* = 5.6 Hz, 1H), ppm; ^{13}C NMR (100 MHz, $CDCl_3$) δ 180.9, 163.4, 157.0, 155.9, 134.3, 133.6, 130.0, 129.6, 127.2, 120.6, 112.1 ppm.

3. Preparation of *N*-protected 7-azaisatin 1e

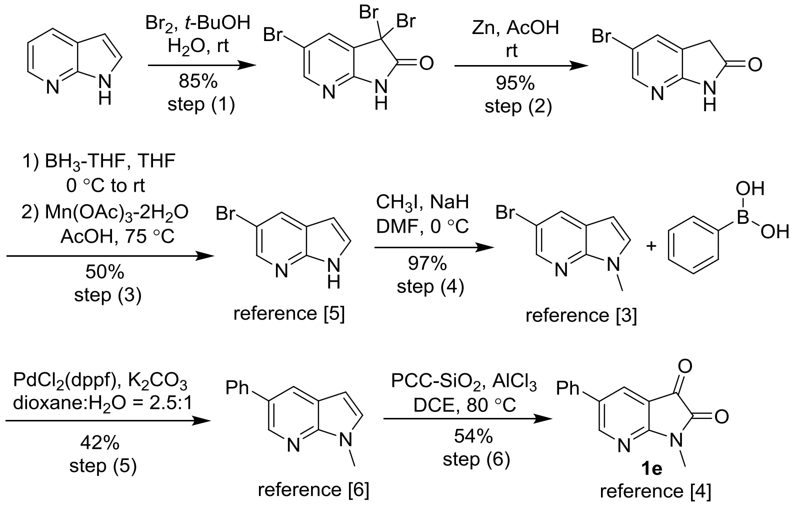

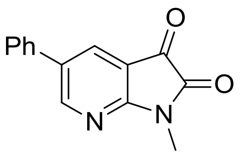

1e: Yellow solid, ^{1}H NMR (400 MHz, $CDCl_3$) δ 8.67 (d, *J* = 2.0 Hz, 1H), 8.02 (d, *J* = 2.0 Hz, 1H), 7.51-7.44 (m, 5H), 3.39 (s, 3H) ppm; ^{13}C NMR (100 MHz, $CDCl_3$) δ 182.0, 162.9, 158.5, 154.0, 136.1, 133.5, 131.2, 129.3, 128.5, 126.6, 111.9, 25.2 ppm.

4. Preparation of N-protected 7-azaisatins 1f and 1g

To a solution of **1a** (0.10 g, 0.6 mmol) in DMF (5 mL) was added NCS or NBS (0.16 g, 0.9 mmol) at room temperature. The solution was stirred for 4 h and then at 50 °C for 41 h. The progress of the reaction was monitored by TLC. After the disappearance of **1a**, the mixture was poured into water (30 mL), followed by extraction with CH_2Cl_2 (10 mL x 3). The combined organic layers were washed three times with distilled water and dried (Na_2SO_4). After removal of the solvent, the residue was purified by flash chromatography on silica gel with dichloromethane as an eluent to give the pure product **1f** and **1g** [7].

5. Preparation of N-protected 7-azaisatin 1h

Compound **1h** was synthesized by literature procedure [8].

6. Preparation of N-substituted maleimides 2a–i

Maleimides **2a–i** were synthesized by known literature procedures [9-13].

7. General procedure for MBH reaction

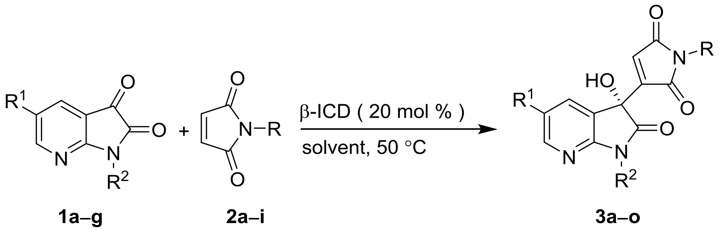

A solution of N-protected 7-azaisatin **1** (0.1 mmol), N-substituted maleimide **2** (0.6 mmol) and catalyst β-ICD (20 mol %) in dry solvent (1.0 mL) was stirred at 50 °C. Purification by flash chromatography on silica gel (EtOAc/petroleum ether) gave the corresponding MBH product **3a–o**.

Typical procedure for the synthesis of 3p and 3q: A solution of *N*-methyl-7-azaisatin (**1**, 0.1 mmol), acrylate (0.6 mmol) and catalyst β-ICD (20 mol %) in dry DCM (1.0 mL) was stirred at 30 °C. Purification by flash chromatography on silica gel (EtOAc/petroleum ether) gave the corresponding MBH product **3p** and **3q**.

Typical procedure for the synthesis of 3r: Acrolein (0.15 mmol) was added dropwise to a solution of 7-azaisatin (0.1 mmol) and β-ICD (10 mol %) in dry DCM (1.0 mL) at −20 °C. The progress of the reaction was monitored by TLC. After completion, the MBH reaction product **3r** was purified by flash chromatography on silica gel with petroleum ether/EtOAc as the eluent.

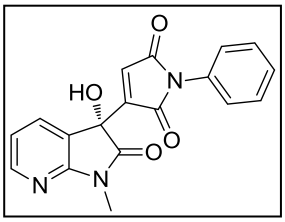

3a: 98% yield, 33.0 mg, light yellow solid, $[\alpha]_D^{20}$ = –80.4 (*c* = 4.4 in $CHCl_3$), 94% ee, determined by HPLC analysis [Daicel Chiralpak AD, (*n*-hexane/*i*-PrOH = 80/20, 1 mL/min, UV 254nm, t_{major} = 9.91 min, t_{minor} = 12.26 min]; ^1H NMR (400 MHz, $CDCl_3$) δ 8.29 (d, *J* = 4.8 Hz, 1H), 7.66 (d, *J* = 6.8 Hz, 1H), 7.42 (t, *J* = 7.6 Hz, 2H), 7.33 (t, *J* = 7.2 Hz, 1H), 7.27-7.25 (m, 2H), 7.03 (dd, *J* = 6.8 Hz, *J* = 5.2 Hz, 1H), 6.94 (s, 1H), 4.24 (brs, 1H), 3.35 (s, 3H) ppm; ^{13}C NMR (150 MHz, $CDCl_3$) δ 174.1, 167.8, 167.5, 157.1, 149.7, 146.1, 132.6, 130.7, 129.4, 129.1, 128.1, 125.8, 122.2, 119.2, 74.0, 25.9 ppm; ESI HRMS: calcd. for $C_{18}H_{13}N_3O_4$+Na^+ 358.0798, found 358.0796.

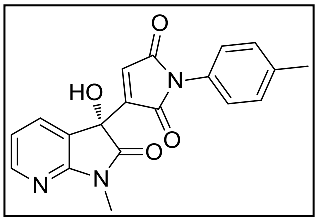

3b: 87% yield, 30.0 mg, light yellow solid, $[\alpha]_D^{20}$ = –69.6 (*c* = 6.5 in $CHCl_3$), 90% ee, determined by HPLC analysis [Daicel Chiralpak AD, (*n*-hexane/*i*-PrOH = 60/40, 1 mL/min, UV 254 nm, t_{major} = 5.40 min, t_{minor} = 6.62 min]; ^1H NMR (400 MHz, $CDCl_3$) δ 8.28 (d, *J* = 5.2 Hz, 1H), 7.65 (d, *J* = 7.2 Hz, 1H), 7.20 (d, *J* = 8.0 Hz, 2H), 7.12(d, *J* = 8.4 Hz, 2H), 7.04-7.01 (m, 1H), 6.93 (s, 1H), 4.50 (brs, 1H), 3.34 (s, 3H), 2.34 (s, 3H) ppm; ^{13}C NMR (100 MHz, $CDCl_3$) δ 174.1, 167.9, 167.6, 157.2, 149.7, 146.0, 138.2, 132.6, 129.7, 129.3, 128.0, 125.7, 122.2, 119.2, 74.0, 25.9, 21.1 ppm; ESI HRMS: calcd. for $C_{19}H_{15}N_3O_4$+Na^+ 372.0955, found 372.0952.

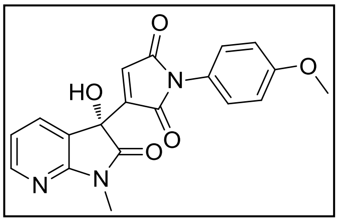

3c: 88% yield, 32.0 mg, yellow solid, $[\alpha]_D^{20}$ = –86.2 (*c* = 10 in $CHCl_3$), 92% ee, determined by HPLC analysis [Daicel Chiralpak AD, (*n*-hexane/*i*-PrOH = 60/40, 1 mL/min, UV 254 nm, t_{major} = 6.80 min, t_{minor} = 8.34 min]; ^1H NMR (400 MHz, $CDCl_3$) δ 8.27 (d, *J* = 5.2 Hz, 1H), 7.64 (d, *J* = 7.2 Hz, 1H), 7.14 (d, *J* = 8.8 Hz, 2H), 7.04-7.01 (m, 1H), 6.93-6.90 (m, 3H), 4.60-4.54 (brs, 1H), 3.79 (s, 3H), 3.33 (s, 3H)

ppm; ^{13}C NMR (100 MHz, $CDCl_3$) δ 174.2, 168.1, 167.7, 159.1, 157.1, 149.6, 146.0, 132.6, 129.3, 127.3, 123.2, 122.3, 119.2, 114.4, 74.0, 55.4, 25.9 ppm; ESI HRMS: calcd. for $C_{19}H_{15}N_3O_5$+Na^+ 388.0904, found 388.0909.

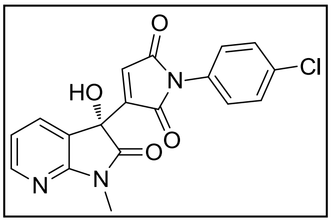

3d: 87% yield, 32.1 mg, white solid, $[\alpha]_D^{20}$ = –94.3 (*c* = 5.5 in $CHCl_3$), 92% ee, determined by HPLC analysis [Daicel Chiralpak AS, (*n*-hexane/*i*-PrOH = 60/40, 1 mL/min, UV 254 nm, t_{minor} = 5.97 min, t_{major} = 8.88 min]; ^{1}H NMR (400 MHz, $CDCl_3$) δ 8.30 (d, *J* = 5.2 Hz, 1H), 7.65 (d, *J* = 7.2 Hz, 1H), 7.26-7.23 (m, 2H), 7.12-7.08 (m, 2H), 7.04 (dd, *J* = 7.2 Hz, *J* = 1.6 Hz, 1H), 6.95 (s, 1H), 4.29 (brs, 1H), 3.35 (s, 3H) ppm; ^{13}C NMR (100 MHz, $CDCl_3$) δ 174.0, 167.6, 167.4, 157.1, 149.8, 146.2, 132.6, 129.4, 127.7, 127.6, 122.0, 119.2, 116.2, 116.0, 74.0, 25.9 ppm; ESI HRMS: calcd. for $C_{18}H_{12}ClN_3O_4$+Na^+ 392.0409, found 392.0403.

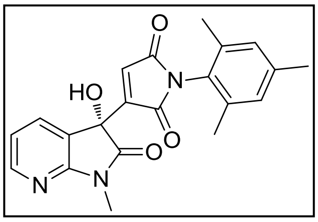

3e: 90% yield, 34.0 mg, white solid, $[\alpha]_D^{20}$ = –71.9 (*c* = 4.4 in $CHCl_3$), 79% ee, determined by HPLC analysis [Daicel Chiralpak IE, (*n*-hexane/*i*-PrOH = 80/20, 1 mL/min, UV 254 nm, t_{major} = 8.80 min, t_{minor} = 9.92 min]; ^{1}H NMR (400 MHz, $CDCl_3$) δ 8.29-8.28 (m, 1H), 7.67 (d, *J* = 7.2 Hz, 1H), 7.03 (dd, *J* = 7.2 Hz, *J* = 1.6 Hz, 1H), 6.92-6.90 (m, 3H), 4.55 (brs, 1H), 3.33 (s, 3H), 2.27 (s, 3H), 2.01 (s, 3H), 1.94 (s, 3H) ppm; ^{13}C NMR (100 MHz, $CDCl_3$) δ 174.0, 168.0, 167.8, 157.2, 149.7, 146.0, 139.5, 136.4, 136.2, 132.4, 129.3, 129.2, 129.0, 125.9, 122.3, 119.2, 74.0, 25.8, 21.0, 17.8, 17.7 ppm; ESI HRMS: calcd. for $C_{21}H_{19}N_3O_4$+Na^+ 400.1268, found 400.1275.

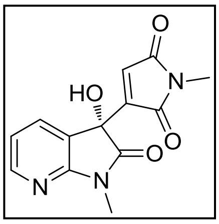

3f: 84% yield, 23.0 mg, white solid, $[\alpha]_D^{20}$ = –114.4 (*c* = 4.0 in $CHCl_3$), 89% ee, determined by HPLC analysis [Daicel Chiralpak ID, (*n*-hexane/*i*-PrOH = 60/40, 1 mL/min, UV 254 nm, t_{major} = 6.17 min, t_{minor} = 7.48 min]; ^{1}H NMR (400 MHz, $CDCl_3$) δ 8.27 (d, *J* = 5.2 Hz, 1H), 7.58 (d, *J* = 7.2 Hz, 1H), 7.03-7.00 (m, 1H), 6.80 (s, 1H), 4.89-4.86 (brs, 1H), 3.33 (s, 3H), 2.92 (s, 3H) ppm; ^{13}C NMR (100 MHz, $CDCl_3$) δ 174.3, 169.1, 168.7, 157.1, 149.6, 146.3, 132.5, 129.2, 122.3, 119.1, 73.9, 25.8, 23.8 ppm; ESI HRMS: calcd. for $C_{13}H_{11}N_3O_4$+Na^+ 296.0642, found 296.0656.

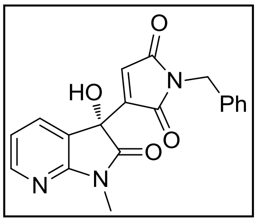

3g: 86% yield, 30.0 mg, white solid, $[\alpha]_D^{20}$ = –79.7 (*c* = 5.0 in $CHCl_3$), 89% ee, determined by HPLC analysis [Daicel Chiralpak AS, (*n*-hexane/*i*-PrOH = 60/40, 1 mL/min, UV 254 nm, t_{minor} = 6.54 min, t_{major} = 15.29 min]; ^{1}H NMR (400 MHz, $CDCl_3$) δ 8.27 (d, *J* = 5.2 Hz, 1H), 7.59 (d, *J* = 7.2 Hz, 1H), 7.26 (m, 5H), 7.02-6.99 (m, 1H), 6.73 (s, 1H), 4.58 (s, 2H), 4.34 (brs, 1H), 3.33 (s, 3H) ppm; ^{13}C NMR (100 MHz, $CDCl_3$) δ 174.0, 168.6, 168.4, 157.1, 149.7, 145.8, 135.6, 132.7, 129.2, 128.7, 128.5, 128.0, 122.0, 119.2, 73.9, 41.7, 25.8 ppm; ESI HRMS: calcd. for $C_{19}H_{15}N_3O_4$+Na^+ 372.0955, found 372.0958.

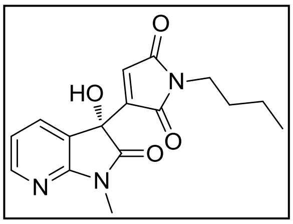

3h: 86% yield, 26.7 mg, white solid, $[\alpha]_D^{20}$ = –62.8 (*c* = 8.0 in $CHCl_3$), 66% ee, determined by HPLC analysis [Daicel Chiralpak IC, (*n*-hexane/*i*-PrOH = 60/40, 1 mL/min, UV 254 nm, t_{minor} = 4.97 min, t_{major} = 6.11 min]; ^{1}H NMR (400 MHz, $CDCl_3$) δ 8.28 (d, *J* = 4.4 Hz, 1H), 7.60 (d, *J* = 7.2 Hz, 1H), 7.02 (dd, *J* = 7.6 Hz, *J* = 1.6 Hz, 1H), 6.73 (s, 1H), 4.52 (brs, 1H), 3.41 (t, *J* = 3.2 Hz, 2H), 3.34 (s, 3H), 1.51-1.46 (m, 2H), 1.27-1.20 (m, 2H), 0.90-0.86 (m, 3H) ppm; ^{13}C NMR (100 MHz, $CDCl_3$) δ 174.2,

169.1, 168.8, 157.1, 149.6, 145.7, 132.6, 129.1, 122.2, 119.1, 73.9, 38.0, 30.4, 25.8, 19.9, 13.5 ppm; ESI HRMS: calcd. for $C_{16}H_{17}N_3O_4+Na^+$ 338.1111, found 338.1107.

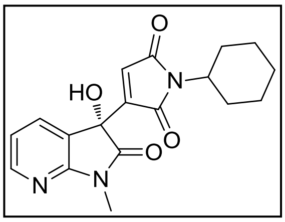

3i: 84% yield, 28.6 mg, white solid, $[\alpha]_D^{20}$ = –80.1 (*c* = 9.5 in $CHCl_3$), 61% ee, determined by HPLC analysis [Daicel Chiralpak ID, (*n*-hexane/*i*-PrOH = 80/20, 1 mL/min, UV 254 nm, t_{major} = 7.10 min, t_{minor} = 8.07 min]; ^{1}H NMR (400 MHz, $CDCl_3$) δ 8.27 (d, *J* = 5.2 Hz, 1H), 7.61 (d, *J* = 7.2 Hz, 1H), 7.02 (dd, *J* = 7.2 Hz, *J* = 1.6 Hz, 1H), 6.66 (s, 1H), 4.45-4.36 (brs, 1H), 3.82-3.77 (m, 1H), 3.34 (s, 3H), 2.01-1.88 (m, 2H), 1.80-1.77 (m, 2H), 1.62-1.60 (m, 3H), 1.30-1.14 (m, 3H) ppm; ^{13}C NMR (100 MHz, $CDCl_3$) δ 174.1, 169.1, 168.9, 157.1, 149.6, 145.3, 132.6, 129.0, 122.4, 119.1, 73.9, 51.2, 29.8, 25.8, 24.9 ppm; ESI HRMS: calcd. for $C_{18}H_{19}N_3O_4+Na^+$ 364.1268, found 364.1275.

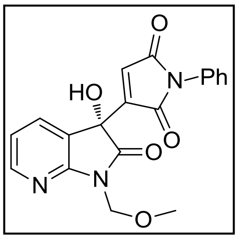

3j: 92% yield, 33.6 mg, light yellow solid, $[\alpha]_D^{20}$ = –86.3 (*c* = 4.0 in $CHCl_3$), 91% ee, determined by HPLC analysis [Daicel Chiralpak AD, (*n*-hexane/*i*-PrOH = 60/40, 1 mL/min, UV 254 nm, t_{major} = 6.45 min, t_{minor} = 7.32 min]; ^{1}H NMR (400 MHz, $CDCl_3$) δ 8.33 (d, *J* = 4.8 Hz, 1H), 7.68 (d, *J* = 7.2 Hz, 1H), 7.44-7.40 (m, 2H), 7.35-7.32 (m, 1H), 7.27-7.25 (m, 2H), 7.08 (dd, *J* = 5.2 Hz, *J* = 1.6 Hz, 1H), 7.00 (s, 1H), 5.27 (s, 2H), 4.48 (brs, 1H), 3.47 (s, 3H) ppm; ^{13}C NMR (100 MHz, $CDCl_3$) δ 174.5, 167.8, 167.5, 156.3, 150.1, 146.0, 133.1, 130.6, 129.6, 129.1, 128.1, 125.8, 121.6, 119.7, 73.9, 70.7, 57.5 ppm; ESI HRMS: calcd. for $C_{19}H_{15}N_3O_5+Na^+$ 388.0904, found 388.0898.

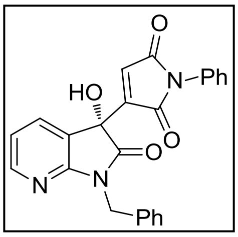

3k: 93% yield, 38.0 mg, light yellow solid, $[\alpha]_D^{20}$ = –99.5 (c = 5.6 in $CHCl_3$), 87% ee, determined by HPLC analysis [Daicel Chiralpak ID, (n-hexane/i-PrOH = 70/30, 1 mL/min, UV 254 nm, t_{major} = 9.66 min, t_{minor} = 10.71 min]; ^{1}H NMR (400 MHz, $CDCl_3$) δ 8.26 (d, J = 4.8 Hz, 1H), 7.65 (d, J = 7.2 Hz, 1H), 7.46 (d, J = 7.2 Hz, 2H), 7.42-7.39 (m, 2H), 7.34-7.28 (m, 3H), 7.26-7.24 (m, 3H), 7.01-6.98 (m, 1H), 6.88 (s, 1H), 5.02 (s, 2H) , 4.57-4.56 (brs, 1H) ppm; ^{13}C NMR (100 MHz, $CDCl_3$) δ 173.7, 167.8, 167.5, 156.7, 149.8, 145.7, 135.5, 132.9, 130.7, 129.4, 129.1, 128.6, 128.3, 128.1, 127.8, 125.8, 122.9, 119.3, 74.0, 43.3 ppm; ESI HRMS: calcd. for $C_{24}H_{17}N_3O_4$+K^+ 450.0851, found 450.0862.

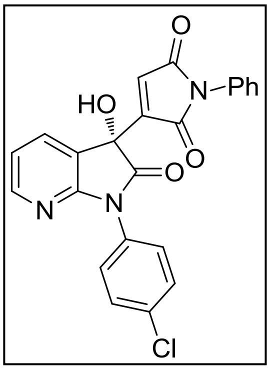

3l: 37% yield, 16.0 mg, light yellow solid, $[\alpha]_D^{20}$ = –58.1 (c = 5.0 in $CHCl_3$), 71% ee, determined by HPLC analysis [Daicel Chiralpak AD, (n-hexane/i-PrOH = 60/40, 1 mL/min, UV 254 nm, t_{major} = 9.10 min, t_{minor} = 15.10 min]; ^{1}H NMR (400 MHz, $CDCl_3$) δ 8.29 (d, J = 4.8Hz, 1H), 7.71 (d, J = 7.6 Hz, 1H), 7.55-7.49 (m, 4H), 7.45-7.41 (m, 2H), 7.36-7.33 (m, 1H), 7.27-7.26 (m, 2H), 7.13-7.10 (m, 1H), 7.05 (s, 1H), 4.10 (brs, 1H) ppm; ^{13}C NMR (100 MHz, $CDCl_3$) δ 173.2, 167.7, 167.5, 156.9, 145.0, 146.6, 134.5, 133.2, 130.8, 130.6, 129.6, 129.4, 129.1, 128.2, 127.9, 125.8, 121.5, 120.0, 74.0 ppm; ESI HRMS: calcd. for $C_{23}H_{14}ClN_3O_4$+Na^+ 454.0565, found 454.0571.

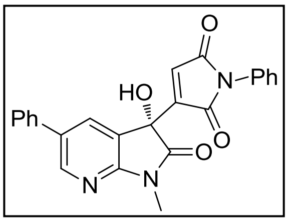

3m: 87.8% yield, 36.0 mg, yellowish solid, $[\alpha]_D^{20}$ = +53.9 (c = 7.5 in $CHCl_3$), 92% ee, determined by HPLC analysis [Daicel Chiralpak AS, (n-hexane/i-PrOH = 60/40, 1 mL/min, UV 254 nm, t_{minor} = 6.00 min, t_{major} = 8.88 min]; ^{1}H NMR (400 MHz, $CDCl_3$) δ 8.49 (d, J = 2.0

Hz, 1H), 7.86 (d, J = 2.0 Hz, 1H), 7.52 (d, J = 7.6 Hz, 2H), 7.47-7.42 (m, 3H), 7.41-7.37 (m, 3H), 7.32 (t, J = 7.2 Hz, 1H), 7.27-7.25 (m, 2H), 7.00 (s, 1H), 4.42 (brs, 1H), 3.39 (s, 3H) ppm; ^{13}C NMR (100 MHz, $CDCl_3$) δ 174.1, 167.6, 167.5, 156.2, 148.0, 146.1, 137.0, 133.1, 131.6, 130.6, 129.4, 129.2, 129.1, 128.1, 128.1, 126.8, 125.8, 122.2, 74.1, 26.0 ppm; ESI HRMS: calcd. for $C_{24}H_{17}N_3O_4$+Na^+ 434.1111, found 434.1108.

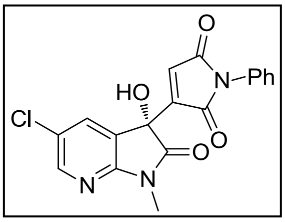

3n: 81% yield, 30.0 mg, white solid, $[\alpha]_D^{20}$ = –18.9 (c = 10.0 in $CHCl_3$), 85% ee, determined by HPLC analysis [Daicel Chiralpak IE, (n-hexane/i-PrOH = 60/40, 1 mL/min, UV 254 nm, t_{major} = 6.61 min, t_{minor} = 7.91 min]; ^{1}H NMR (400 MHz, $CDCl_3$) δ 8.24 (d, J = 2.4 Hz, 1H), 7.63 (d, J = 1.6. Hz, 1H), 7.44-7.40 (m, 2H), 7.36-7.32 (m, 1H), 7.27-7.25 (m, 2H), 7.00 (s, 1H), 4.74 (brs, 1H), 3.32 (s, 3H) ppm; ^{13}C NMR (100 MHz, $CDCl_3$) δ 173.9, 167.6, 167.4, 155.4, 148.2, 145.6, 133.0, 130.5, 129.7, 129.1, 128.2, 127.1, 125.8, 123.1, 73.9, 26.0 ppm; ESI HRMS: calcd. for $C_{18}H_{12}ClN_3O_4$+Na^+ 392.0409, found 392.0412.

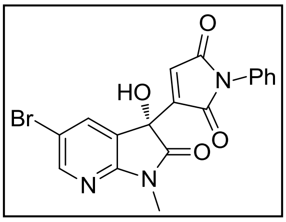

3o: 82% yield, 34.0 mg, yellow solid, $[\alpha]_D^{20}$ = +4.86 (c = 7.0 in $CHCl_3$), 88% ee, determined by HPLC analysis [Daicel Chiralpak ID, (n-hexane/i-PrOH = 60/40, 1 mL/min, UV 254 nm, t_{major} = 5.36 min, t_{minor} = 6.45 min]; ^{1}H NMR (400 MHz, $CDCl_3$) δ 8.34 (d, J = 2.0 Hz, 1H), 7.74 (d, J = 2.4 Hz, 1H), 7.43-7.40 (m, 2H), 7.35-7.32 (m, 1H), 7.27-7.25 (m, 2H), 7.00 (s, 1H), 4.70 (brs, 1H), 3.31 (s, 3H) ppm; ^{13}C NMR (150 MHz, $CDCl_3$) δ 173.8, 167.6, 167.4, 155.7, 150.4, 145.6, 135.4, 130.6, 129.8, 129.1, 128.1, 125.8, 123.6, 114.6, 73.9, 26.0 ppm; ESI HRMS: calcd. for $C_{18}H_{12}BrN_3O_4$+Na^+ 435.9903, found 435.9910.

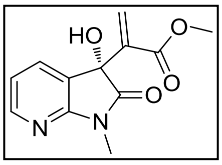

3p: 93% yield, 23.0 mg, white solid, $[\alpha]_D^{20}$ = –76.8 (*c* = 7.5 in $CHCl_3$), 92% ee, determined by HPLC analysis [Daicel Chiralpak AS, (*n*-hexane/*i*-PrOH = 60/40, 1 mL/min, UV 254 nm, tminor = 4.65 min, tmajor = 6.67 min]; ^{1}H NMR (400 MHz, $CDCl_3$) δ 8.20 (d, *J* = 5.2 Hz, 1H), 7.42 (dd, *J* = 7.2 Hz, J = 6.0 Hz, 1H), 6.94 (dd, *J* = 7.2 Hz, *J* = 2.0 Hz, 1H), 6.60 (s, 1H), 6.47 (s, 1H), 4.38 (brs , 1H), 3.63 (s, 3H), 3.31(s, 3H) ppm; ^{13}C NMR (100 MHz, $CDCl_3$) δ 176.2, 164.8, 157.8, 148.6, 138.4, 131.2, 128.4, 124.3, 118.5, 75.7, 52.2, 25.5 ppm; ESI HRMS: calcd. for $C_{12}H_{12}N_2O_4+Na^+$ 271.0689, found 271.0697.

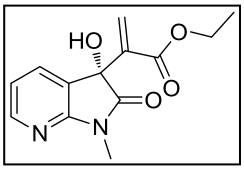

3q: 98% yield, 26.0 mg, white solid, $[\alpha]_D^{20}$ = –95.4 (*c* = 5.0 in $CHCl_3$), 91% ee, determined by HPLC analysis [Daicel Chiralpak AS, (*n*-hexane/*i*-PrOH = 60/40, 1 mL/min, UV 254 nm, tminor = 4.49 min, tmajor = 6.13 min]; ^{1}H NMR (400 MHz, $CDCl_3$) δ 8.19 (d, *J* = 5.2 Hz, 1H), 7.41 (d, *J* = 7.2 Hz, 1H), 6.93 (dd, *J* = 6.8 Hz, *J* = 1.2 Hz, 1H), 6.61 (s, 1H), 6.44 (s, 1H), 4.46-4.40 (brs , 1H), 4.06-4.02 (m, 2H), 3.29 (s, 3H), 1.14-1.10 (m, 3H) ppm; ^{13}C NMR (100 MHz, $CDCl_3$) δ 176.3, 164.3, 157.8, 148.5, 138.6, 131.2, 128.5, 124.5, 118.5, 75.6, 61.1, 25.5, 13.8 ppm; ESI HRMS: calcd. for $C_{13}H_{14}N_2O_4+Na^+$ 285.0846, found 285.0852.

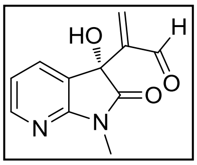

3r: 96% yield, 20.9 mg, white solid, $[\alpha]_D^{20}$ = –171.4 (*c* = 7.0 in $CHCl_3$), 94% ee, determined by HPLC analysis [Daicel Chiralpak AS, (*n*-hexane/*i*-PrOH = 60/40, 1 mL/min, UV 254 nm, tminor = 5.62 min, tmajor = 7.13 min]; ^{1}H NMR (400 MHz, $CDCl_3$) δ 9.48 (m, 1H), 8.21 (d, *J* = 4.4 Hz, 1H), 7.39 (d, *J* = 6.8 Hz, 1H), 7.00 (s, 1H), 6.95-6.92 (m, 1H), 6.44 (s, 1H), 4.19-4.14 (brs, 1H), 3.32 (s, 3H) ppm; ^{13}C NMR (100 MHz, $CDCl_3$) δ 191.7, 175.6, 157.6, 148.8,

148.0, 136.5, 131.3, 123.6, 118.6, 75.0, 25.6 ppm; ESI HRMS: calcd. for $C_{11}H_{10}N_2O_3+Na^+$ 241.0584, found 241.0584.

8. References

1. Nakamoto, Y.; Urabe, F.; Takahashi, K.; Ishihara, J.; Hatakeyama, S. *Chem. – Eur. J.* **2013**, *19*, 12653–12656. doi: 10.1002/chem.201302665

2. Waldmann, H.; Khedkar, V.; Dückert, H.; Schürmann, M.; Oppel, I. M.; Kumar, K. *Angew. Chem. Int. Ed.* **2008**, *47*, 6869–6872. doi: 10.1002/anie.200802413

3. Kannaboina, P.; Anilkumar, K.; Aravinda, S.; Vishwakarma, R. A.; Das, P. *Org. Lett.* **2013**, *15*, 5718–5721. doi: 10.1021/ol4027478

4. Sriram, R.; Kumar, C. N. S. S. P.; Raghunandan, N.; Ramesh, V.; Sarangapani, M.; Rao. V. J. *Synth. Commun.* **2012**, *42*, 3419–3428. doi: 10.1080/00397911.2011.584008

5. Wu, P.-W.; Hsieh, W.-T.; Cheng, Y.-M.; Wei, C.-Y.; Chou, P.-T. *J. Am. Chem. Soc.* **2006**, *128*, 14426–14427. doi: 10.1021/ja064310e

6. Hammond, M.; Washburn, D.-J. et al *Bioorg. Med. Chem. Lett.* **2009**, *19*, 4441–4445. doi: 10.1016/j.bmcl.2009.05.051

7. Tatsugi, J.; Tong, Z. W.; Tsuchiya, Y.; Takayoshi, I. *Arkivoc* **2009**, *2*, 132–137. doi: 10.3998/ark.5550190.0010.214

8. Yan, W.-J.; Wang, D.; Feng, J.-C.; Li, P.; Zhao, D.-P.; Wang, R. *Org. Lett.* **2012**, *14*, 2512–2515. doi: 10.1021/ol3007953

9. Gaidamaviciute. G.; Tauraite, D.; Gagilas, J.; Lagunavicius, A. *Biochimica et Biophysica Act.* **2010**, *1804*, 1385–1393. doi: 10.1016/j.bbapap.2010.01.024

10. Akalay, D.; Dürner, G.; Bats, J. W.; Göbel, M. W. *Beilstein J. Org. Chem.* **2008**, *4*, No. 28. doi:10.3762/bjoc.4.28

11. Samgina, T. Y.; Gorshkov, V. A.; Vorontsov, E. A.; Bagrov, V. V.; Nifant’ev, I. E.; Lebedev, A. T. *Anal. Chem.* **2010**, *65*, 1320–1327. doi: 10.1134/S1061934810130034

12. Hota, S. K.; Chatterjee, A.; Bhattacharya, P. K.; Chattopadhyay. P. *Green Chem.* **2009**, *11*, 169–176. doi: 10.1039/b812290c

13. Rajalakshmi, R.; Chezhian, S.; Arulmani, R. *Elixir Appl. Chem.* **2014**, *72*, 25375–25378.

9 NMR spectra and HPLC chromatograms

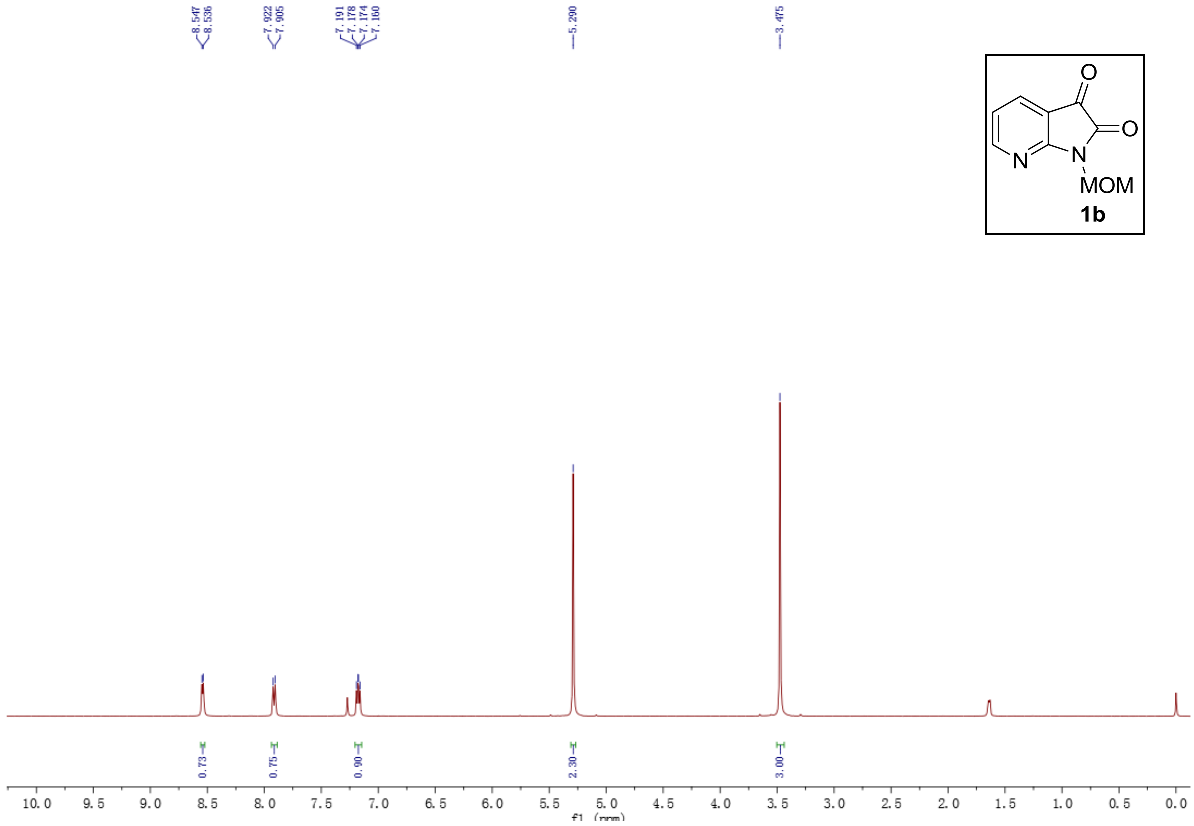

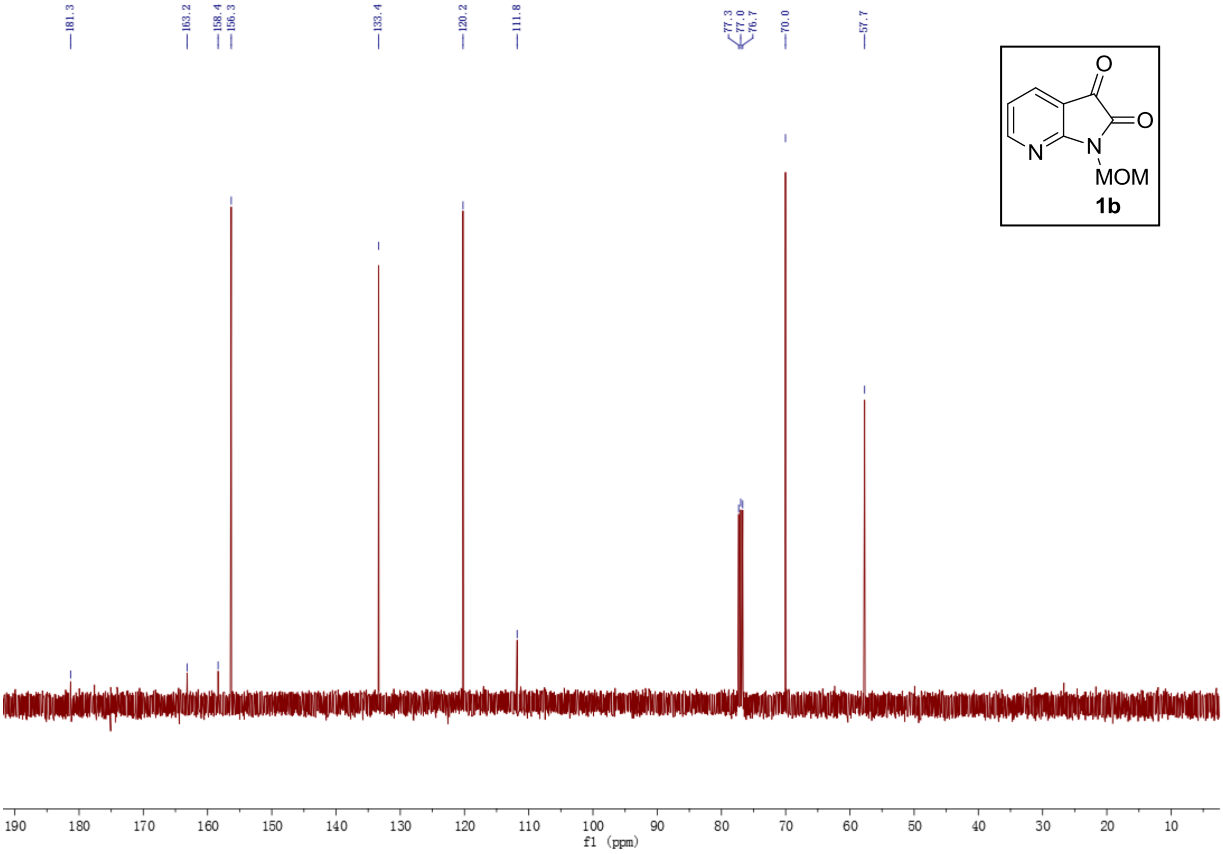

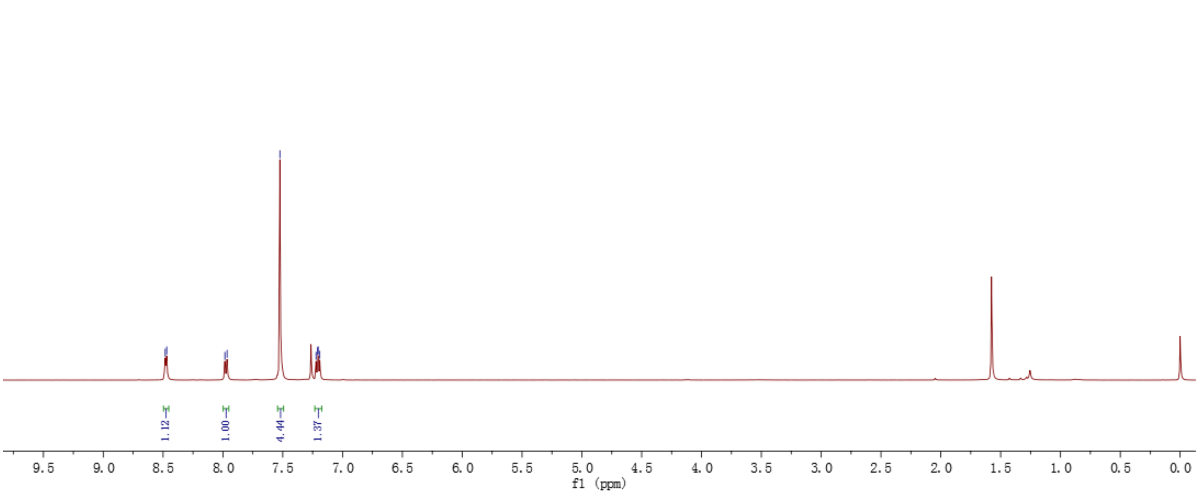
8.48
8.47
7.98
7.97
7.52
7.22
7.21
7.20
7.19
1.12
1.00
4.44
1.37
f1 (ppm)

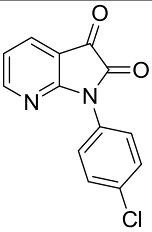

1d

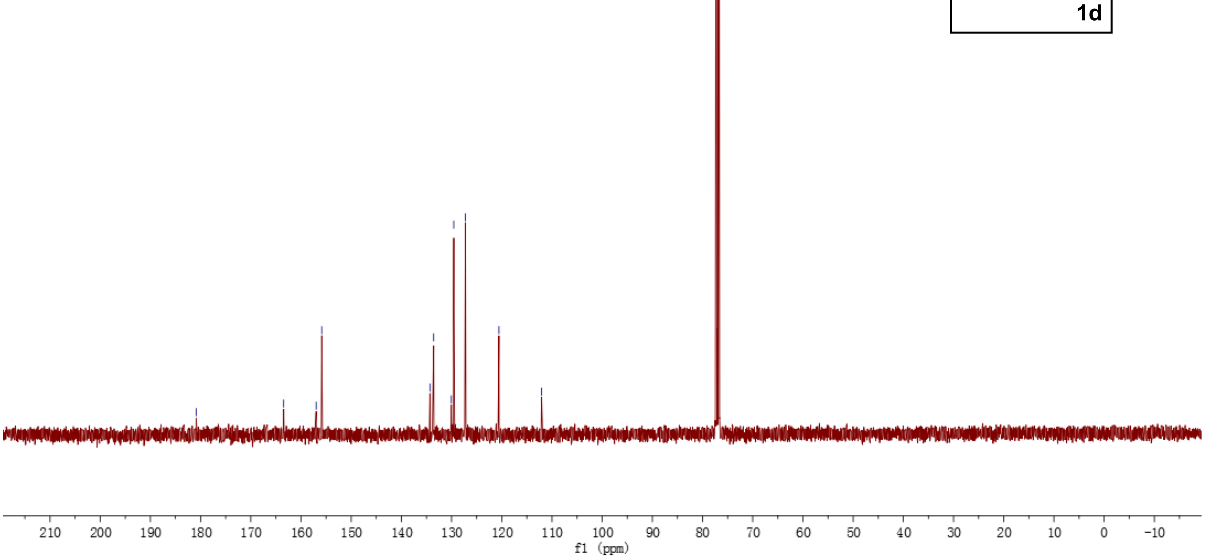
180.9
163.4
157.0
155.9
134.3
133.6
130.0
129.6
127.2
120.6
112.1
77.3
76.7
f1 (ppm)

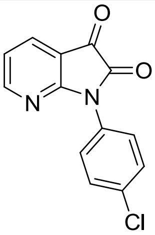

1d

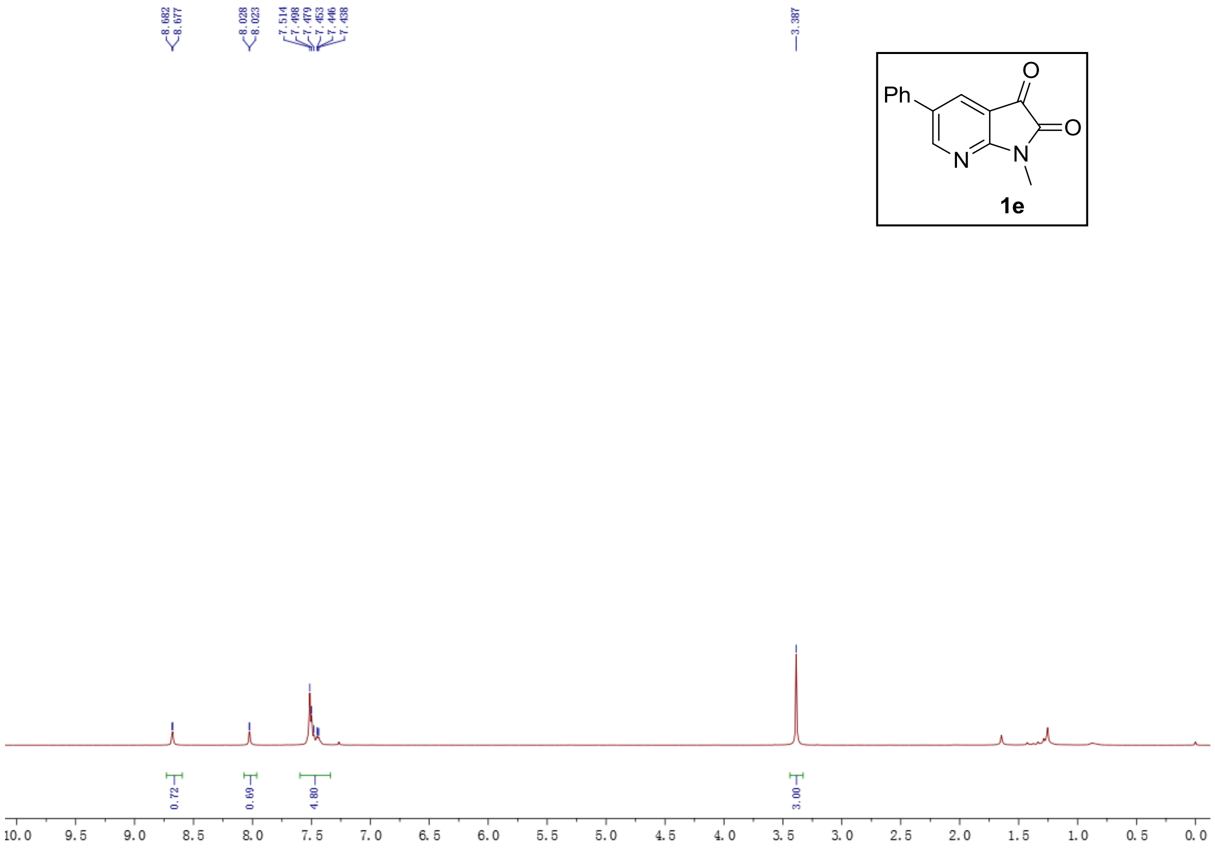
8.682
8.677
8.028
8.023
7.514
7.498
7.479
7.453
7.446
7.438
3.387
Ph
O
O
N
N
1e
0.72
0.69
4.80
3.00
10.0 9.5 9.0 8.5 8.0 7.5 7.0 6.5 6.0 5.5 5.0 4.5 4.0 3.5 3.0 2.5 2.0 1.5 1.0 0.5 0.0

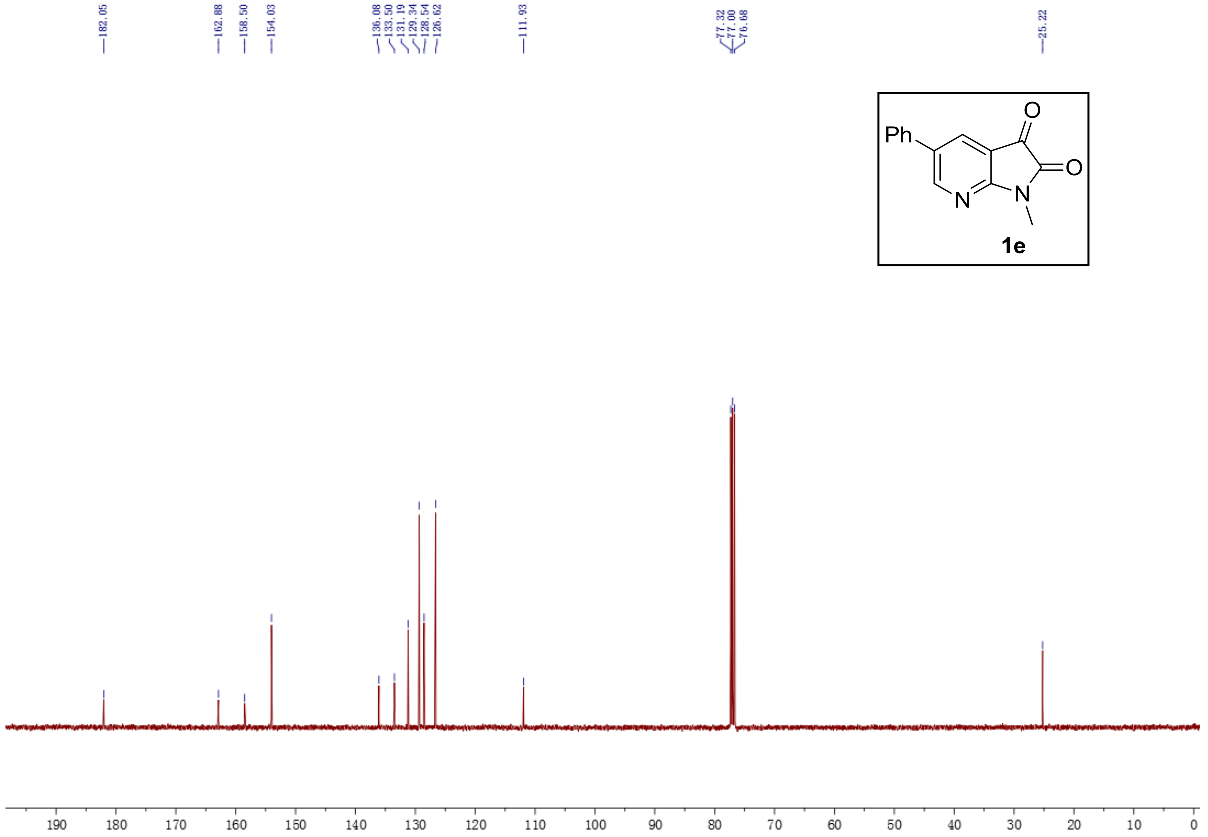
182.05
162.88
158.50
154.03
136.08
133.50
131.19
129.34
128.54
126.62
111.93
77.32
77.00
76.68
25.22
Ph
O
O
N
N
1e
190 180 170 160 150 140 130 120 110 100 90 80 70 60 50 40 30 20 10 0

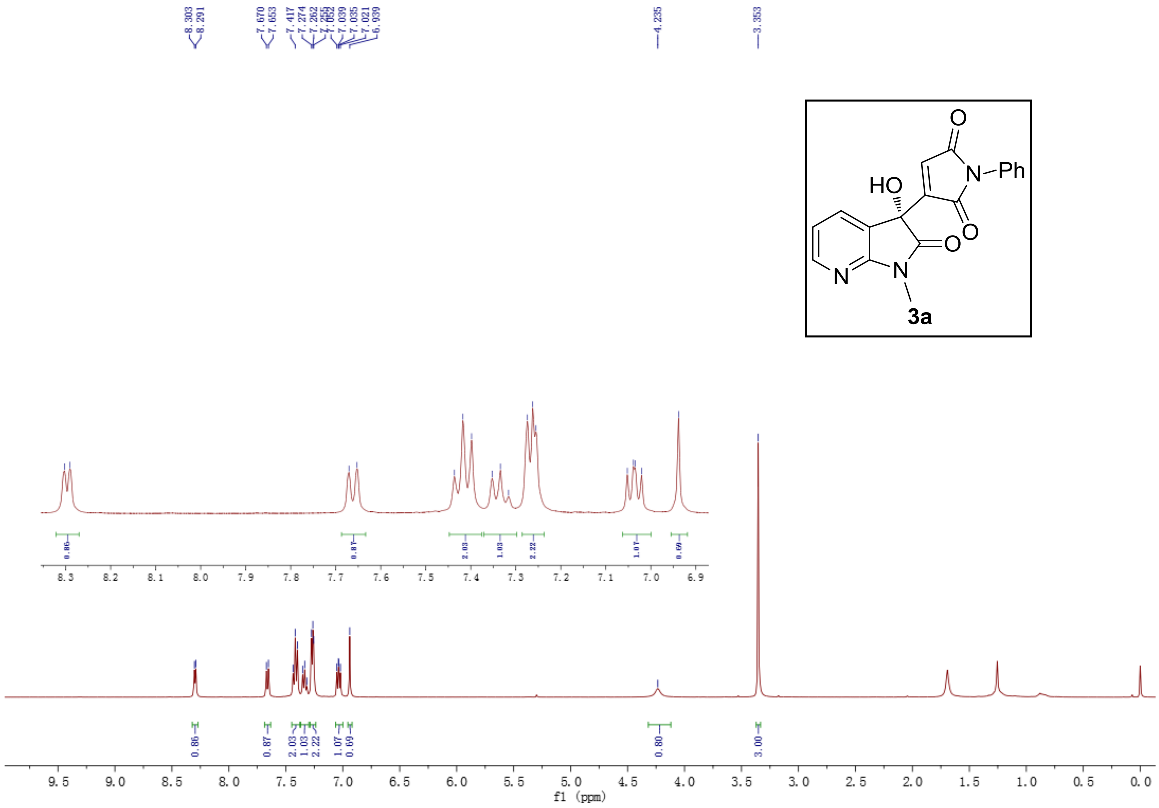
8.303
8.291
7.670
7.653
7.417
7.274
7.262
4.235
3.353
HO
N–Ph
N
3a
f1 (ppm)

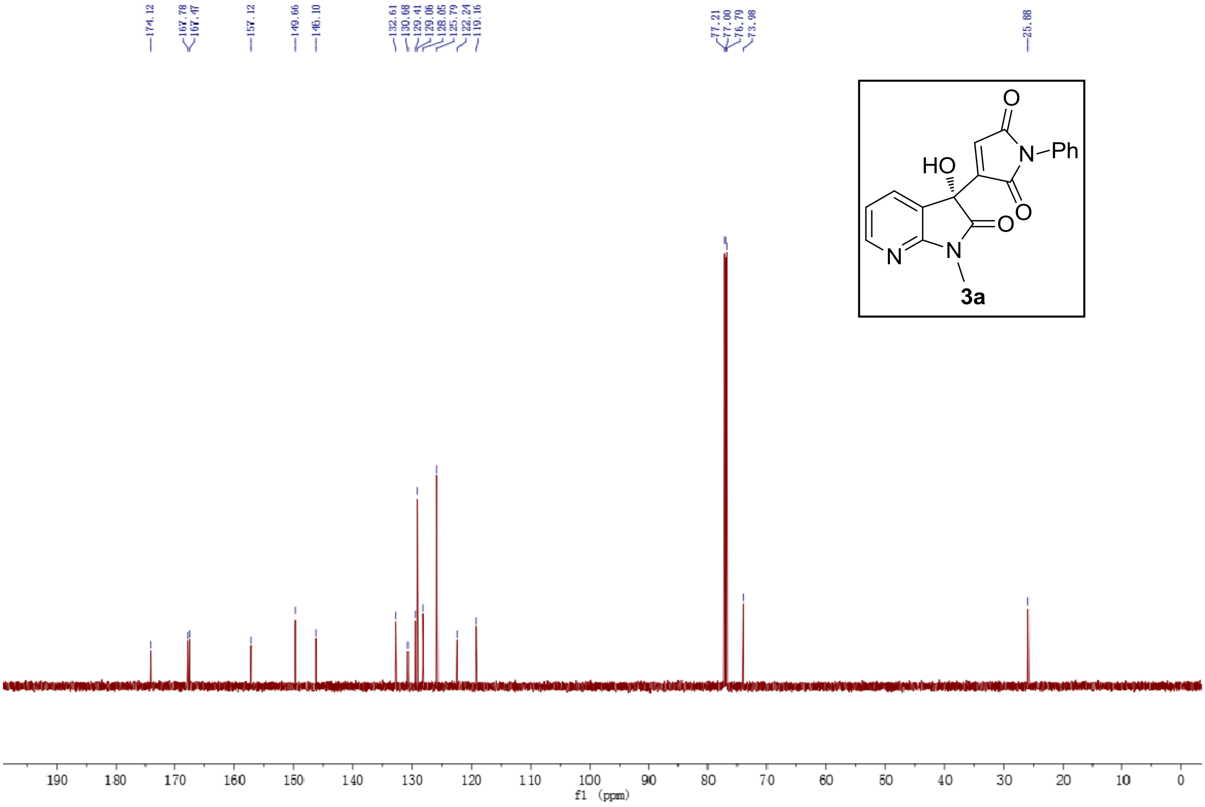
174.12
167.78
167.47
157.12
149.66
146.10
132.61
130.68
129.41
129.06
128.05
125.79
122.24
119.16
77.21
77.00
76.79
73.98
25.88
HO
N–Ph
N
3a
f1 (ppm)

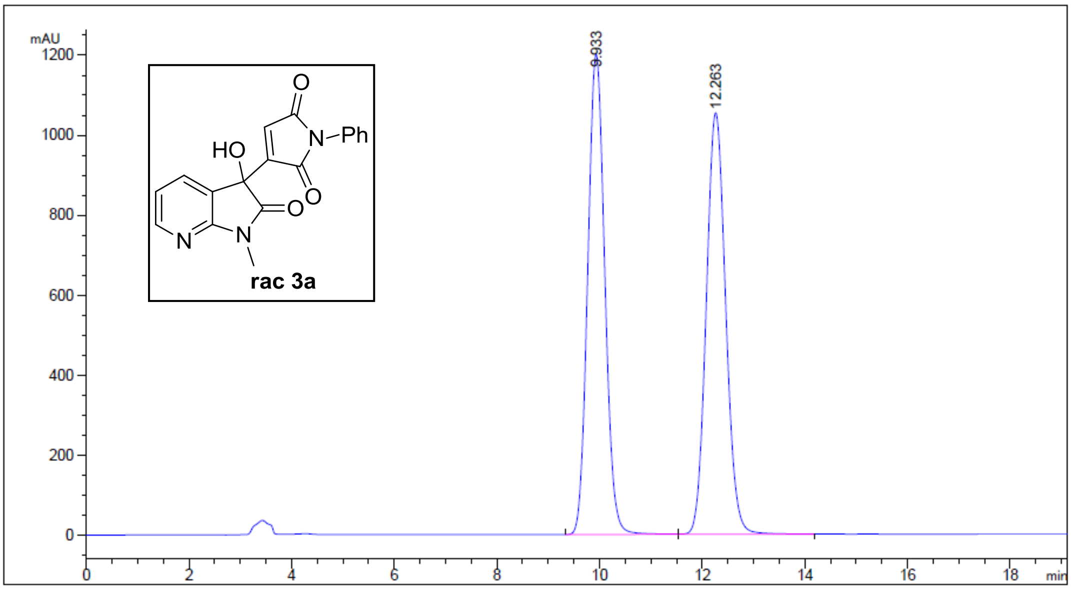

Peak #	RetTime [min]	Type	Width [min]	Area mAU *s	Height [mAU]	Area %
1	9.933	BV	0.3564	2.76765e4	1200.24585	49.9758
2	12.263	VB	0.4115	2.77033e4	1053.84790	50.0242

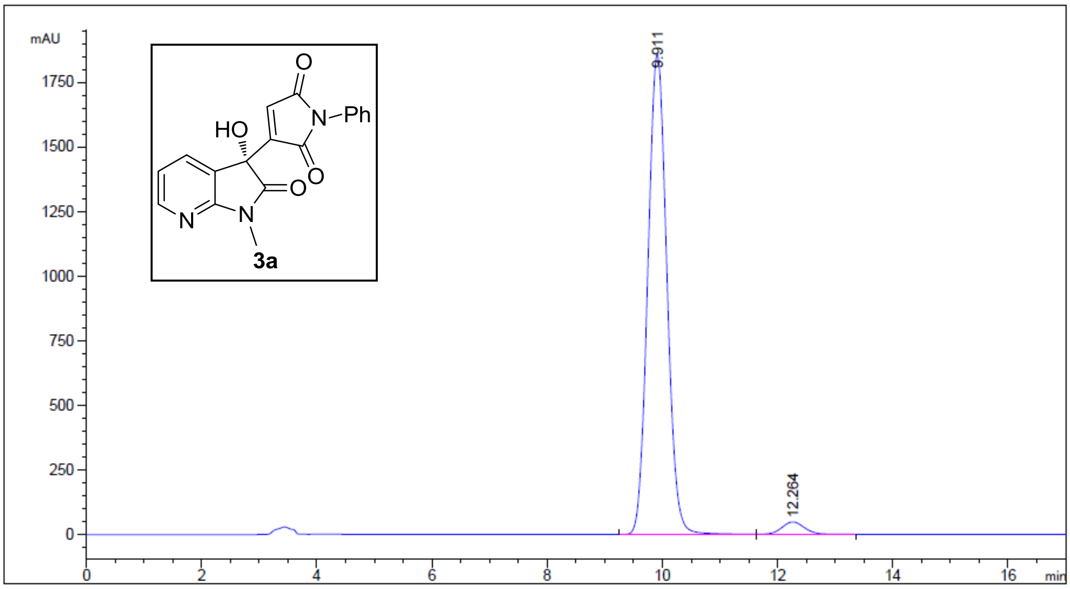

Peak #	RetTime [min]	Type	Width [min]	Area mAU *s	Height [mAU]	Area %
1	9.911	BV	0.3543	4.24612e4	1855.76794	96.9691
2	12.264	VB	0.4279	1327.19275	47.92737	3.0309

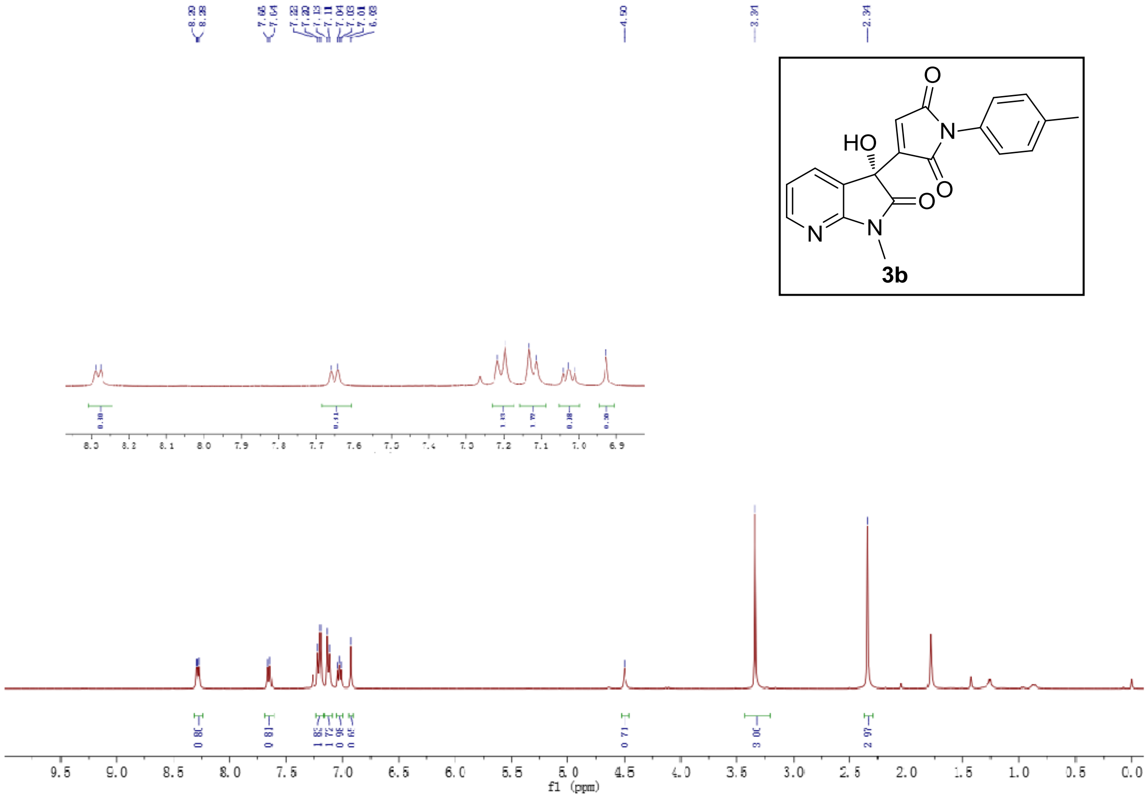
3b
f1 (ppm)

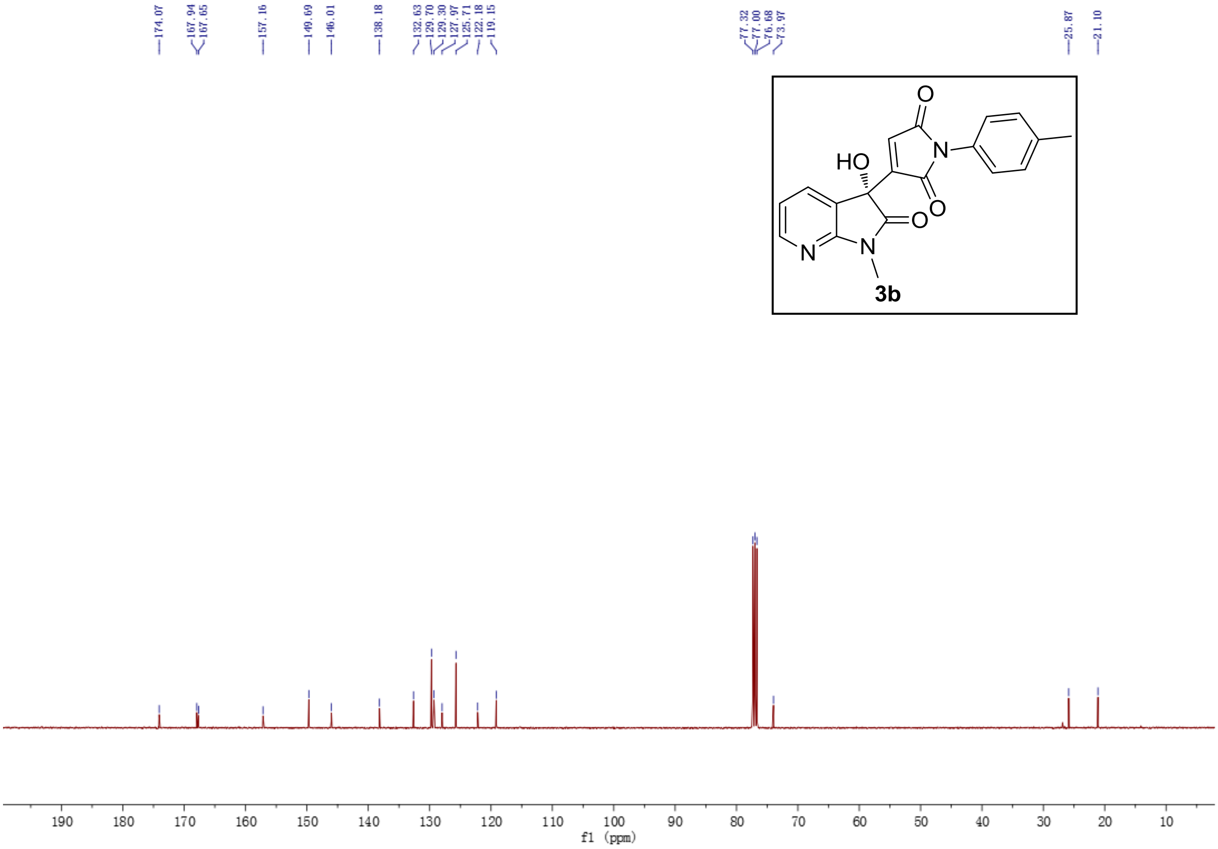
3b
f1 (ppm)

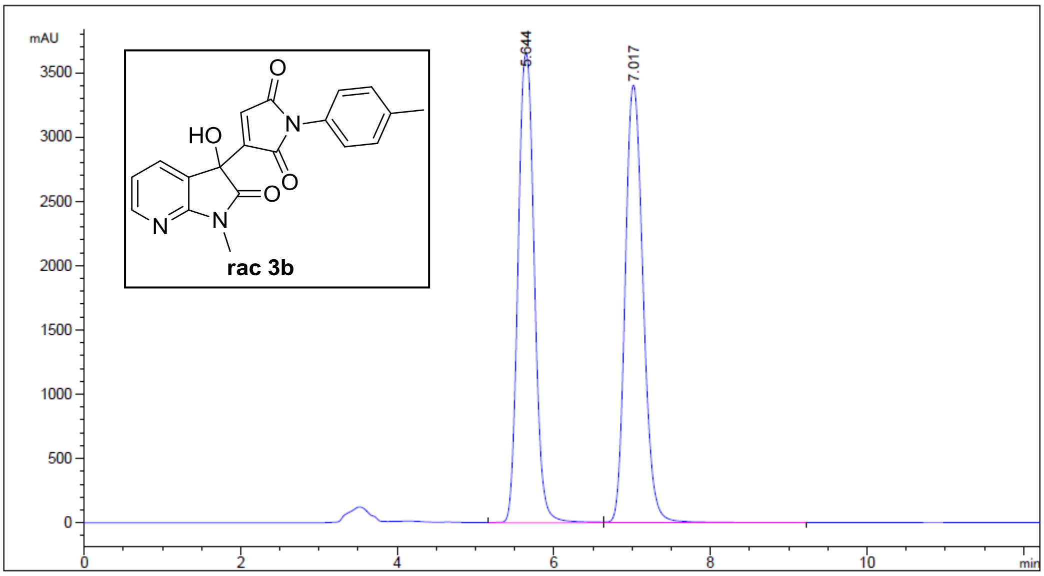

Peak #	RetTime [min]	Type	Width [min]	Area mAU *s	Height [mAU]	Area %
1	5.644	VV	0.2267	5.26513e4	3646.11646	49.3181
2	7.017	VB	0.2497	5.41073e4	3400.50415	50.6819

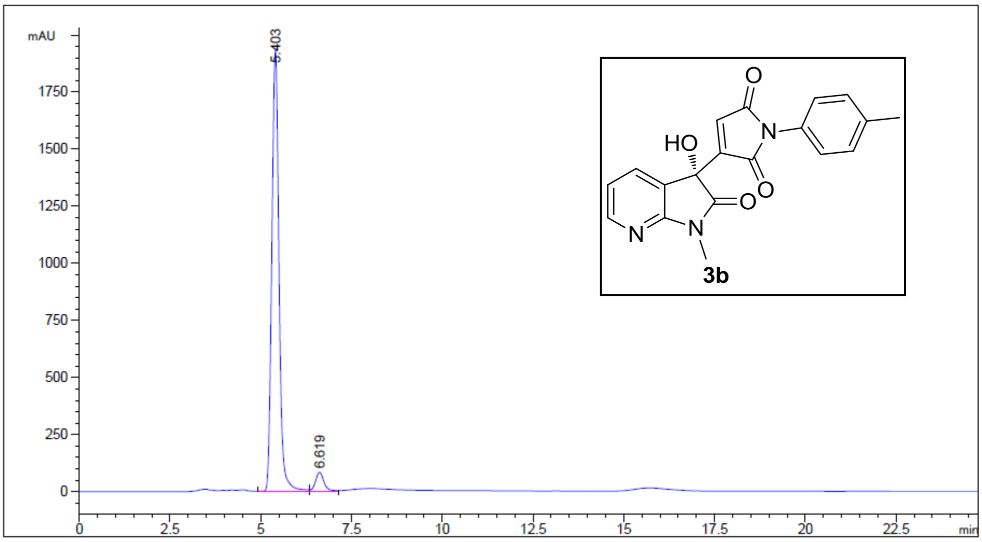

Peak #	RetTime [min]	Type	Width [min]	Area mAU *s	Height [mAU]	Area %
1	5.403	VV	0.2128	2.64968e4	1927.74768	95.0458
2	6.619	VV	0.2509	1381.13953	83.61592	4.9542

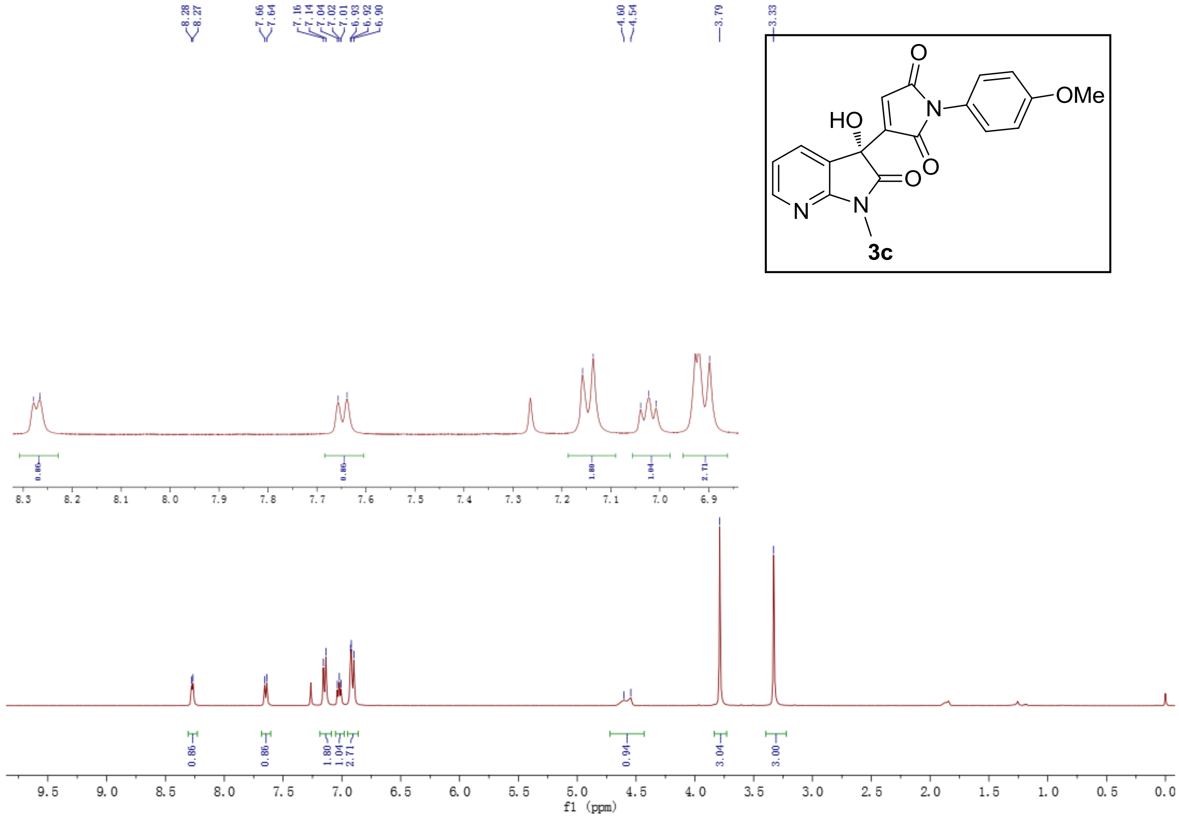
HO
OMe
3c
f1 (ppm)

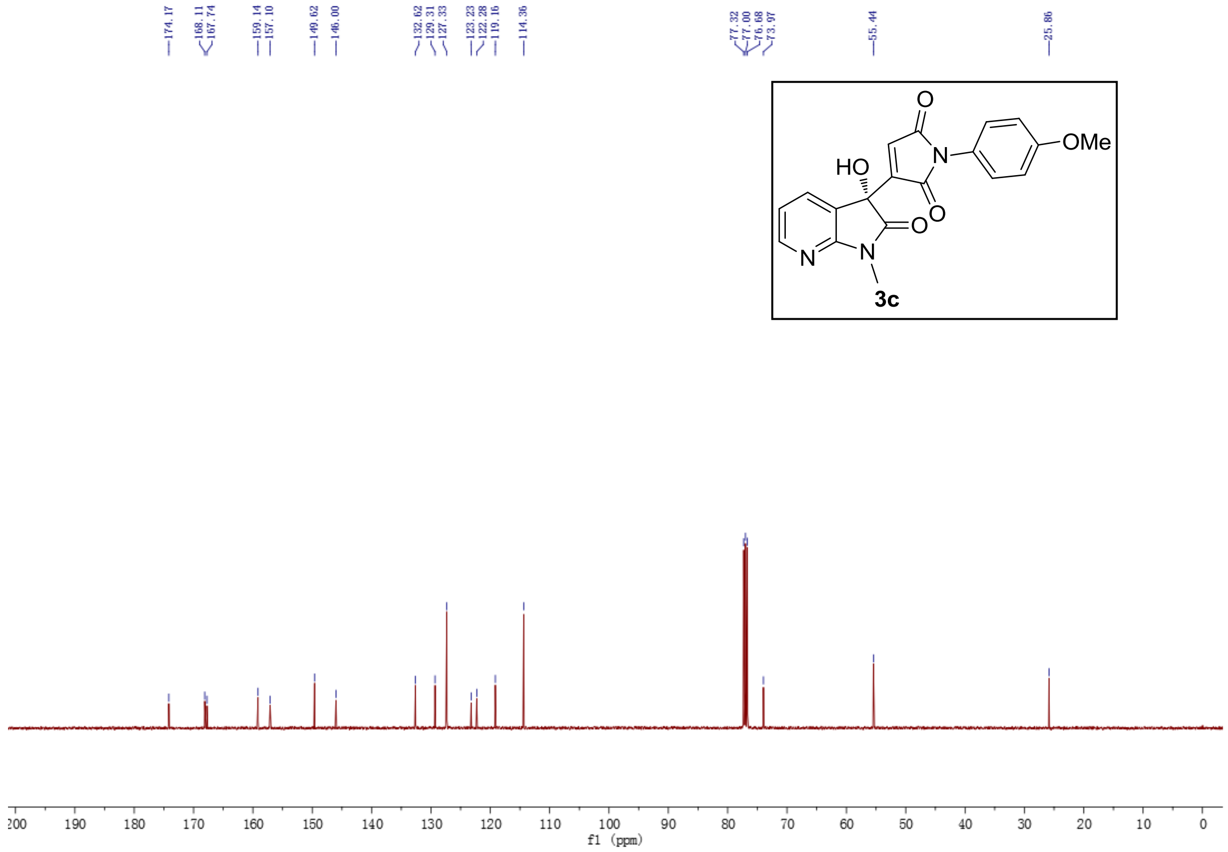
HO
OMe
3c
f1 (ppm)

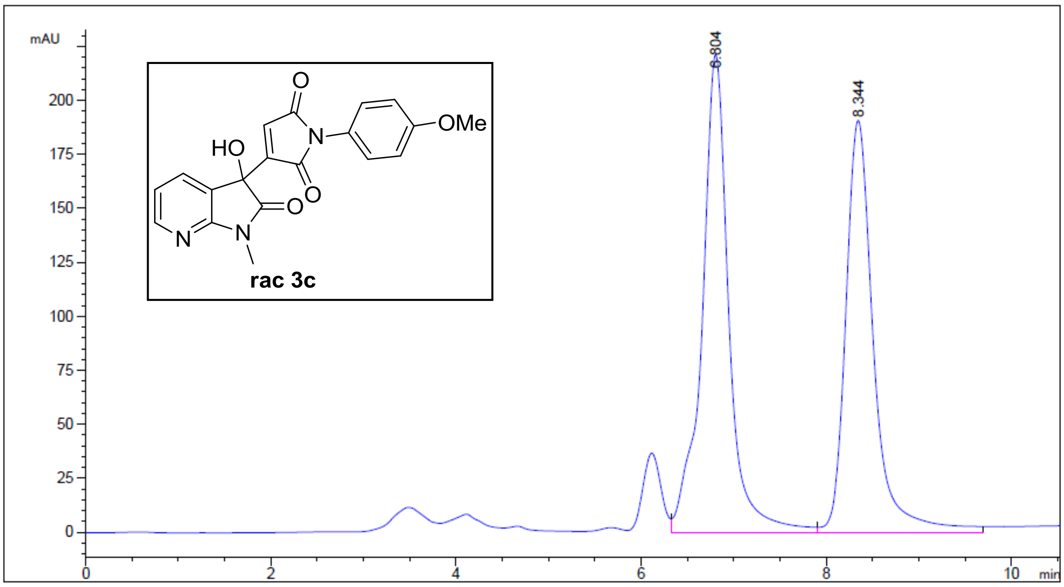

Peak #	RetTime [min]	Type	Width [min]	Area mAU *s	Height [mAU]	Area %
1	6.804	VV	0.2999	4470.23682	221.12601	52.6916
2	8.344	VB	0.3154	4013.54565	190.71477	47.3084

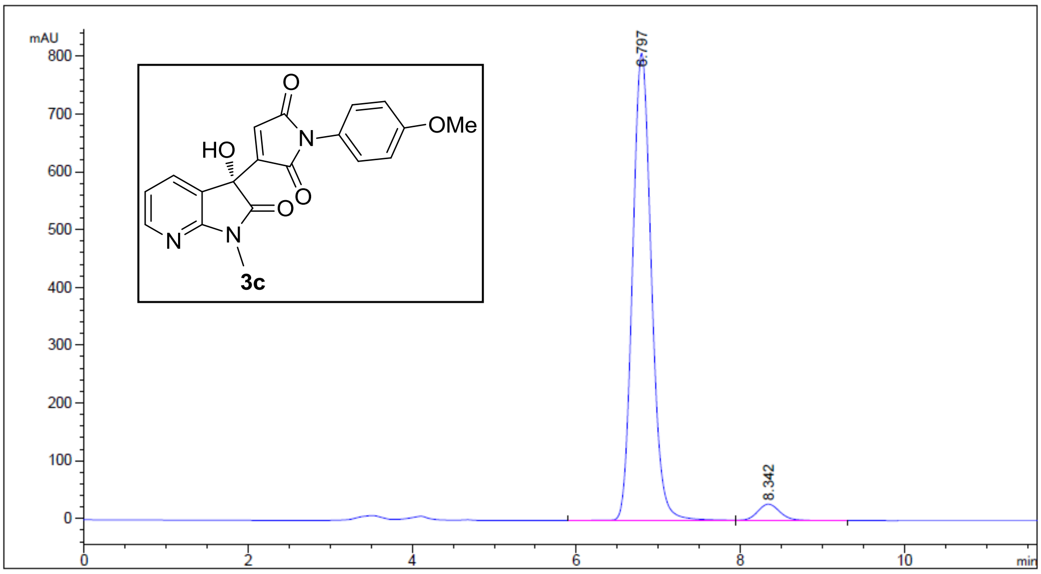

Peak #	RetTime [min]	Type	Width [min]	Area mAU *s	Height [mAU]	Area %
1	6.797	VV	0.2504	1.28962e4	807.57745	95.8827
2	8.342	VB	0.2957	553.77148	28.60598	4.1173

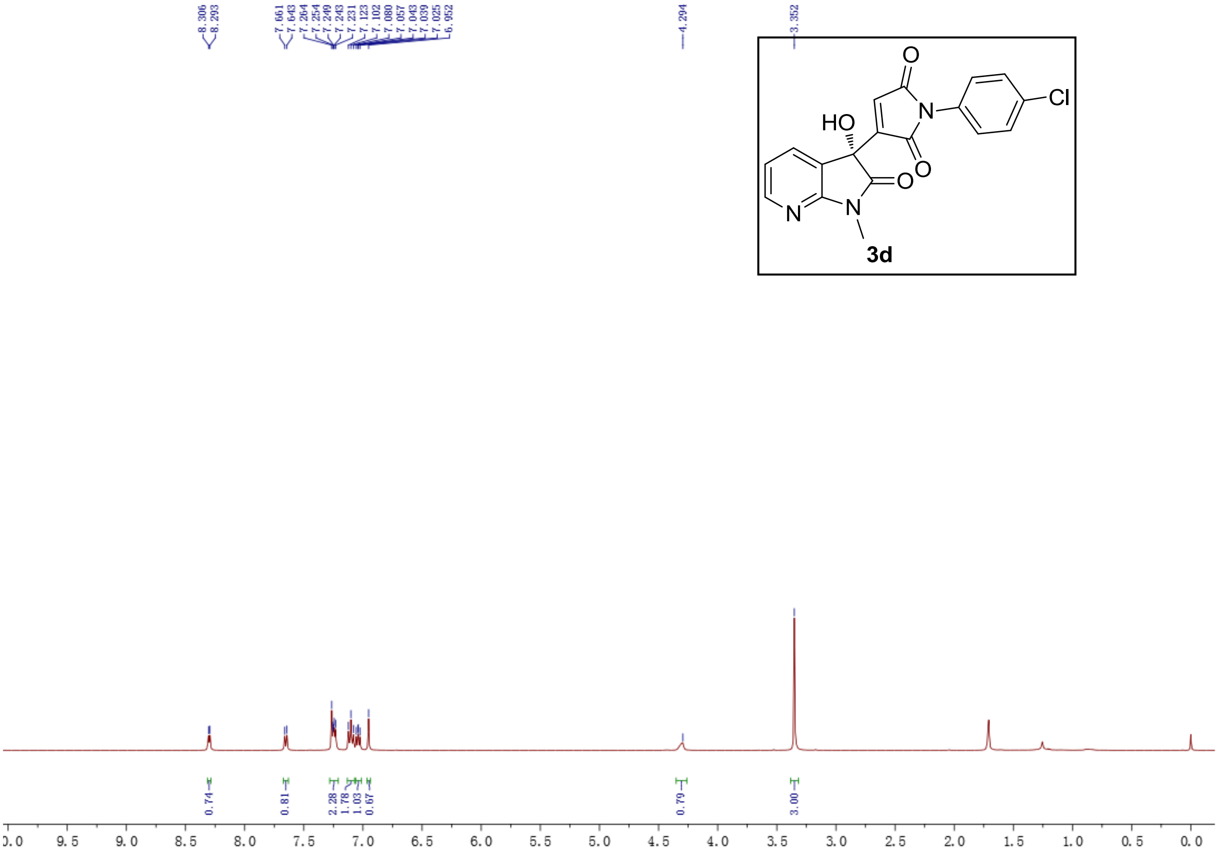
8.306
8.293
7.661
7.643
7.264
7.254
7.249
7.243
7.231
7.123
7.102
7.080
7.057
7.043
7.039
7.025
6.952
4.294
3.352
HO
O
N
Cl
N
N
3d
0.74
0.81
2.28
1.78
1.03
0.67
0.79
3.00

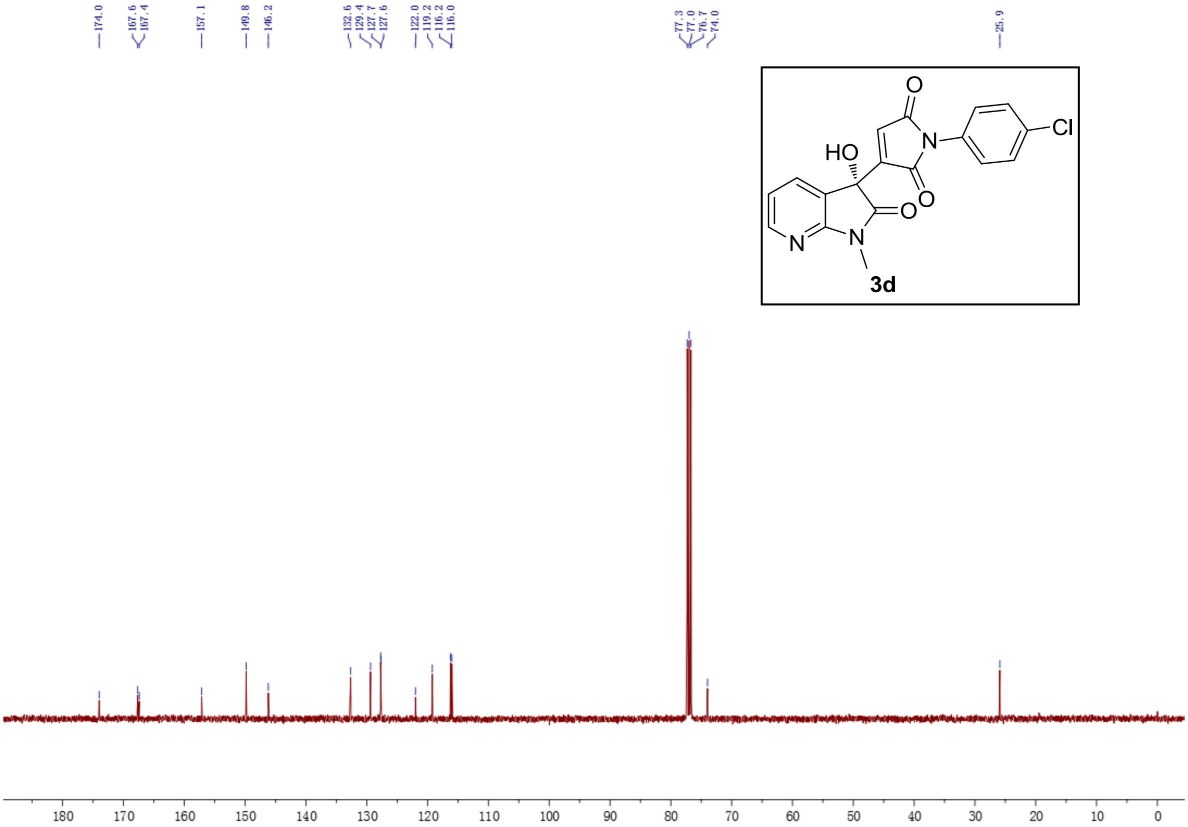
174.0
167.6
167.4
157.1
149.8
146.2
132.6
129.4
127.7
127.6
122.0
119.2
116.2
116.0
77.3
77.0
76.7
74.0
25.9
HO
O
N
Cl
N
N
3d

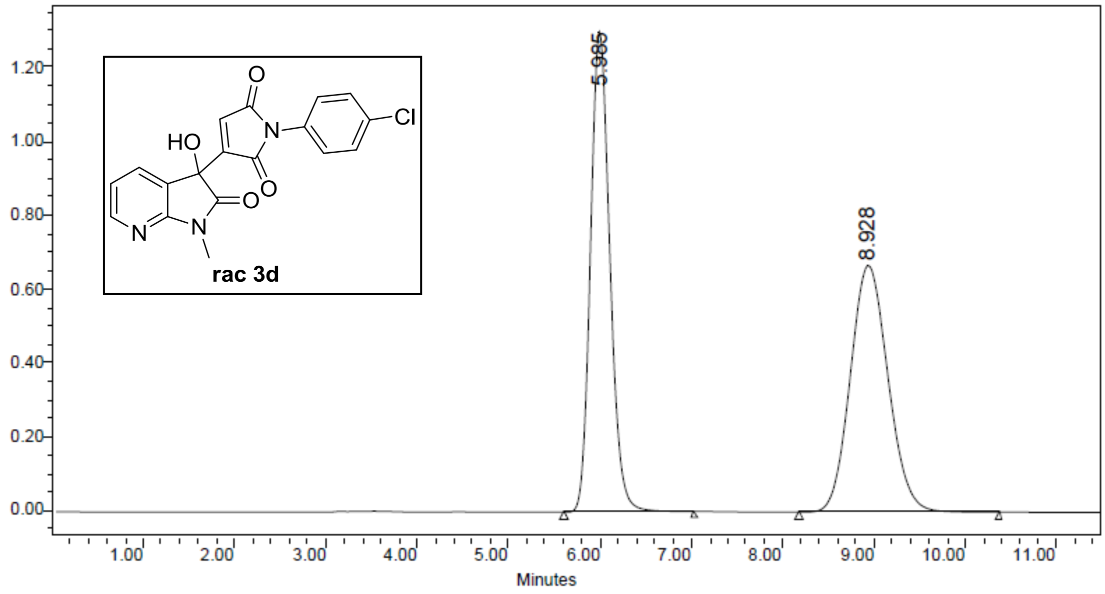

	RT (min)	Area (*sec)	% Area	Height ( )	% Height
1	5.985	19128519	49.91	1299253	66.06
2	8.928	19197621	50.09	667649	33.94

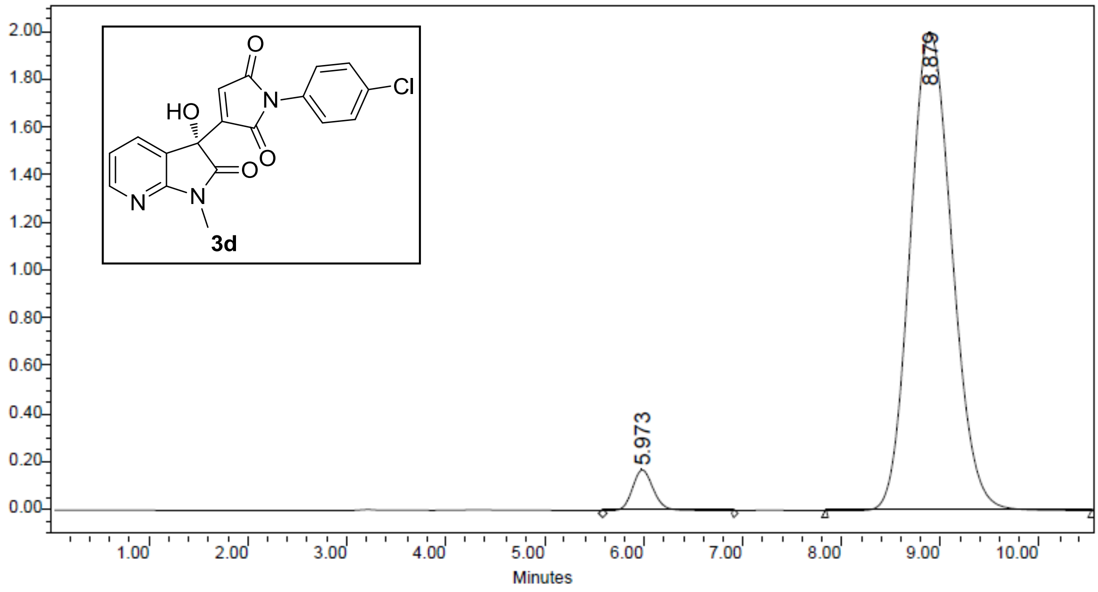

	RT (min)	Area (*sec)	% Area	Height ( )	% Height
1	5.973	2497291	4.06	170177	7.83
2	8.879	58975772	95.94	2002944	92.17

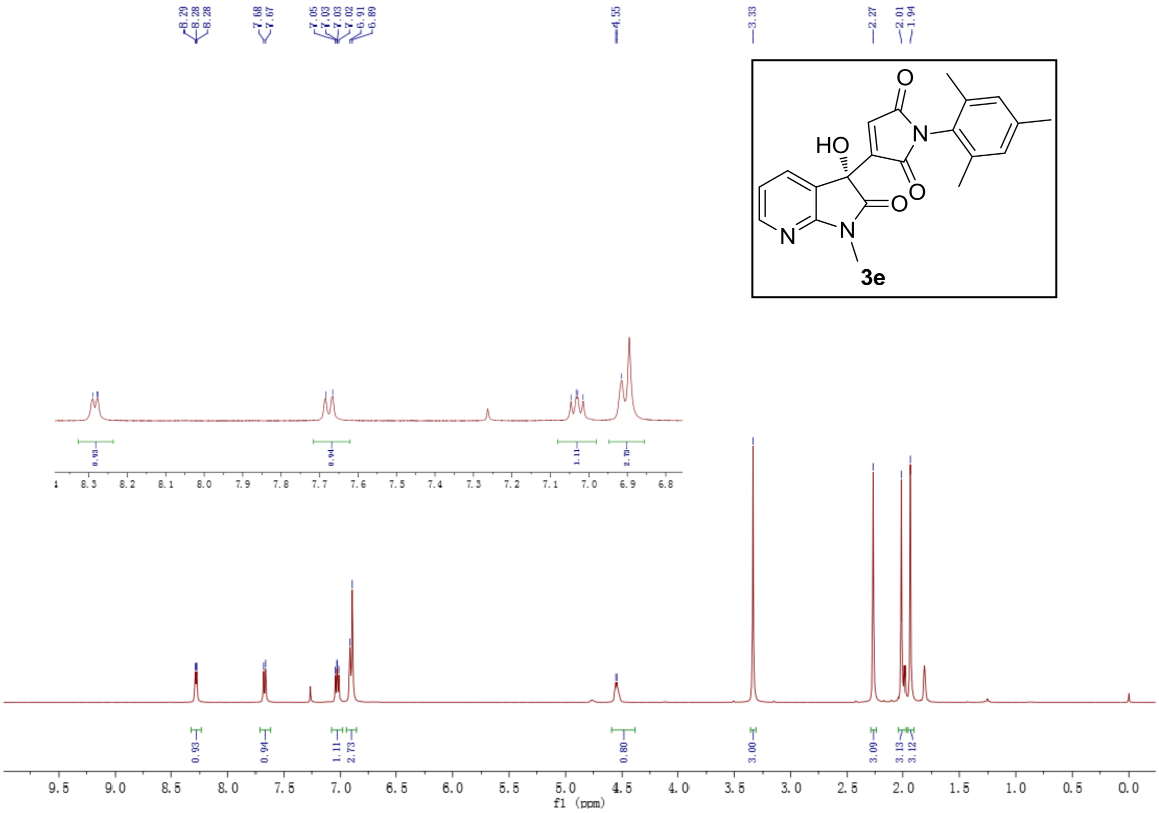
8.29
8.28
8.28
7.68
7.67
7.05
7.03
7.03
7.02
6.91
6.89
4.55
3.33
2.27
2.01
1.94
HO
O
N
O
N
N
O
3e
0.93
0.94
1.11
2.73
0.80
3.00
3.09
3.13
3.12
f1 (ppm)

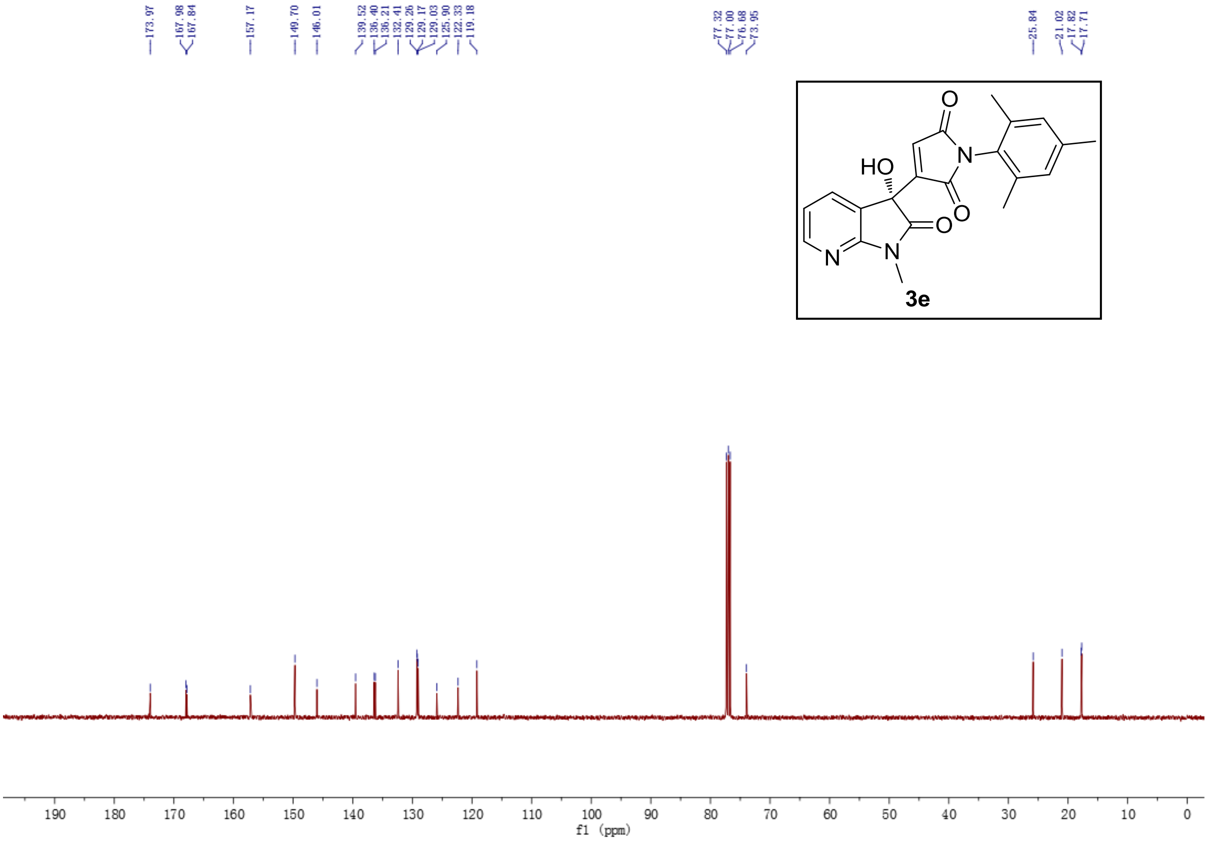
173.97
167.98
167.84
157.17
149.70
146.01
139.52
136.40
136.21
132.41
129.26
129.17
129.03
125.90
122.33
119.18
77.32
77.00
76.68
73.95
25.84
21.02
17.82
17.71
HO
O
N
O
N
N
O
3e
f1 (ppm)

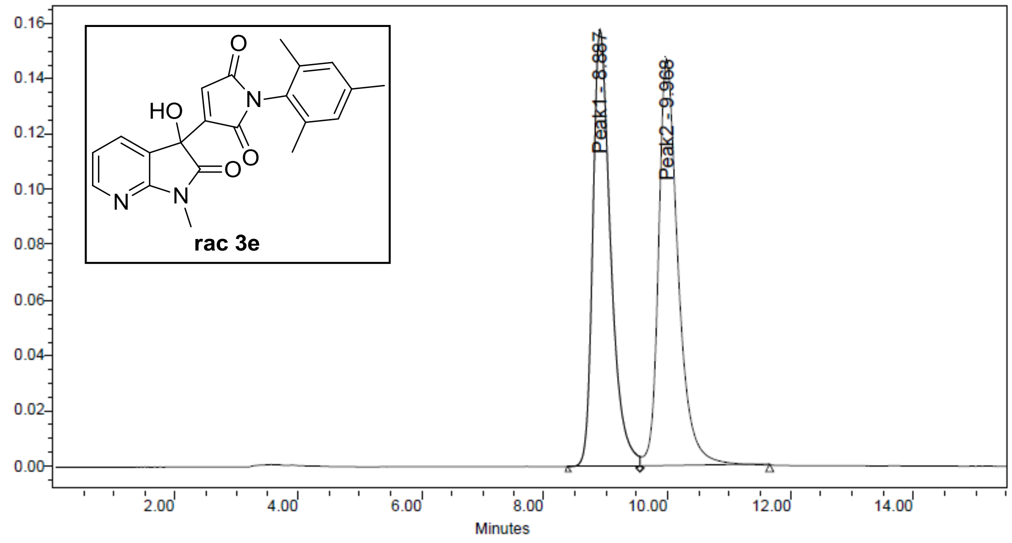

	Peak Name	RT (min)	Area (*sec)	% Area	Height ( )	% Height
1	Peak1	8.887	3160968	49.24	157631	51.76
2	Peak2	9.968	3258195	50.76	146938	48.24

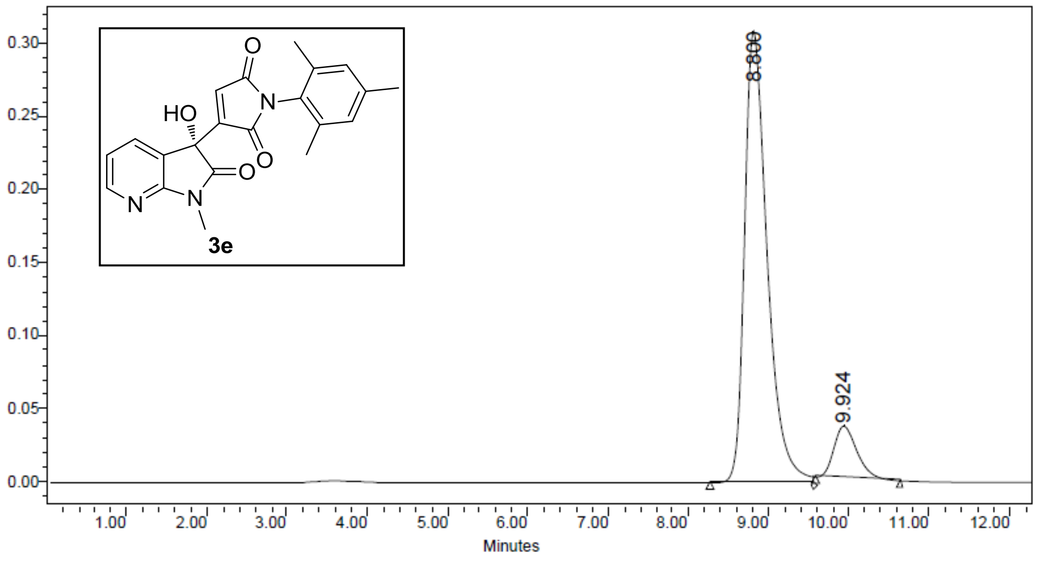

	RT (min)	Area (*sec)	% Area	Height ( )	% Height
1	8.800	6060208	89.34	309007	89.65
2	9.924	722961	10.66	35679	10.35

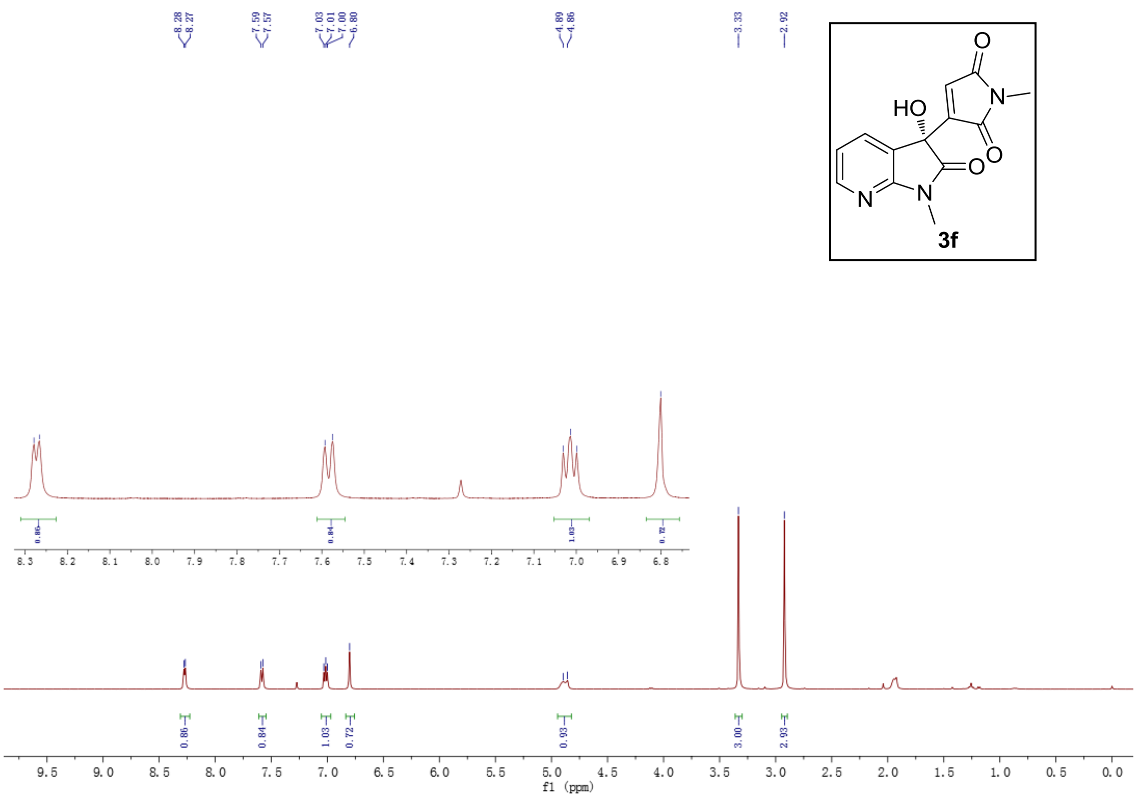
8.28
8.27
7.59
7.57
7.03
7.01
7.00
6.80
4.89
4.86
3.33
2.92
HO
N
O
3f
0.86
0.84
1.03
0.72
0.93
3.00
2.93
f1 (ppm)

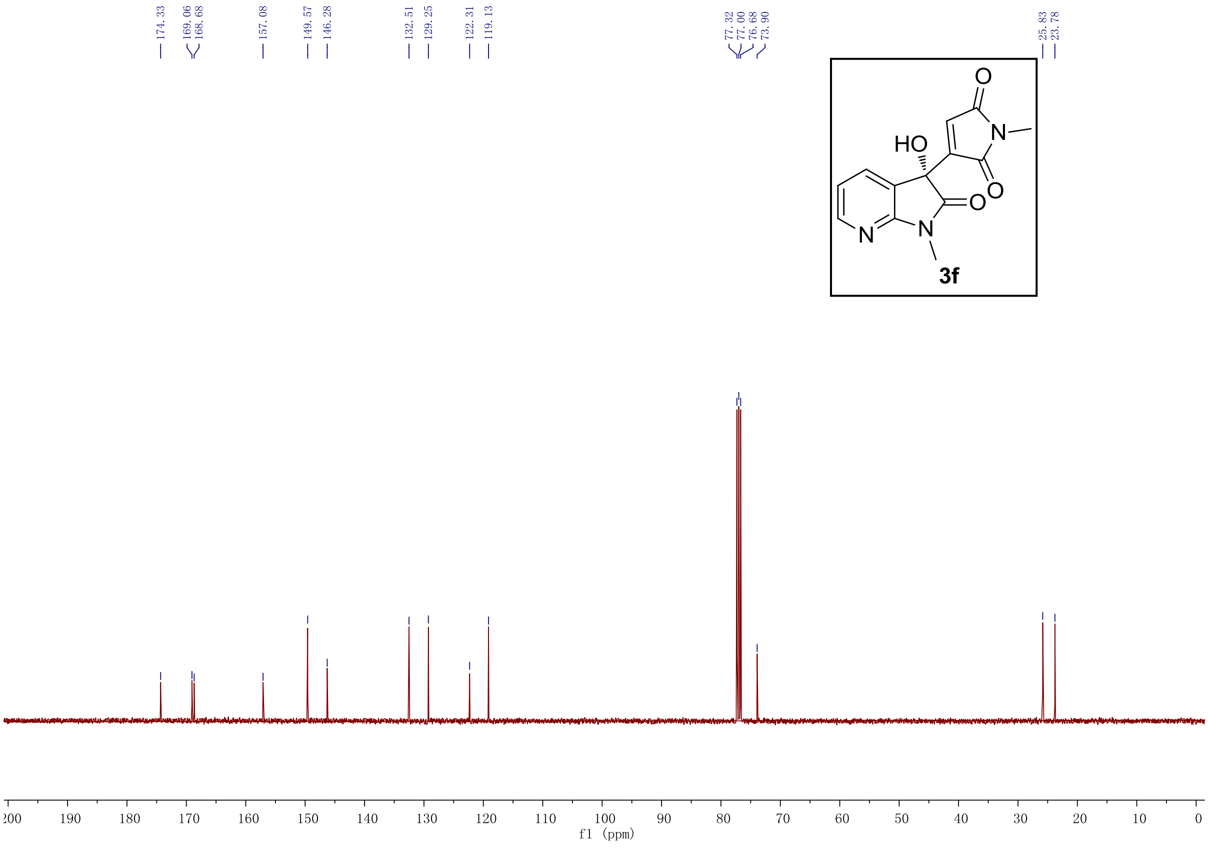
174.33
169.06
168.68
157.08
149.57
146.28
132.51
129.25
122.31
119.13
77.32
77.00
76.68
73.90
25.83
23.78
HO
N
O
3f
f1 (ppm)

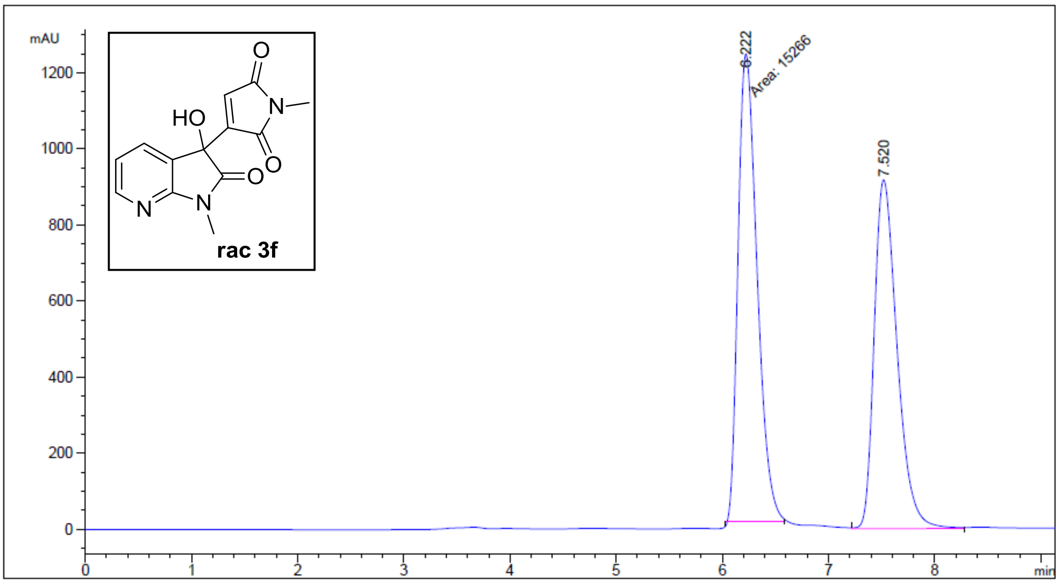

Peak #	RetTime [min]	Type	Width [min]	Area mAU *s	Height [mAU]	Area %
1	6.222	MM	0.2066	1.52660e4	1231.45105	52.6886
2	7.520	VV	0.2271	1.37080e4	915.30383	47.3114

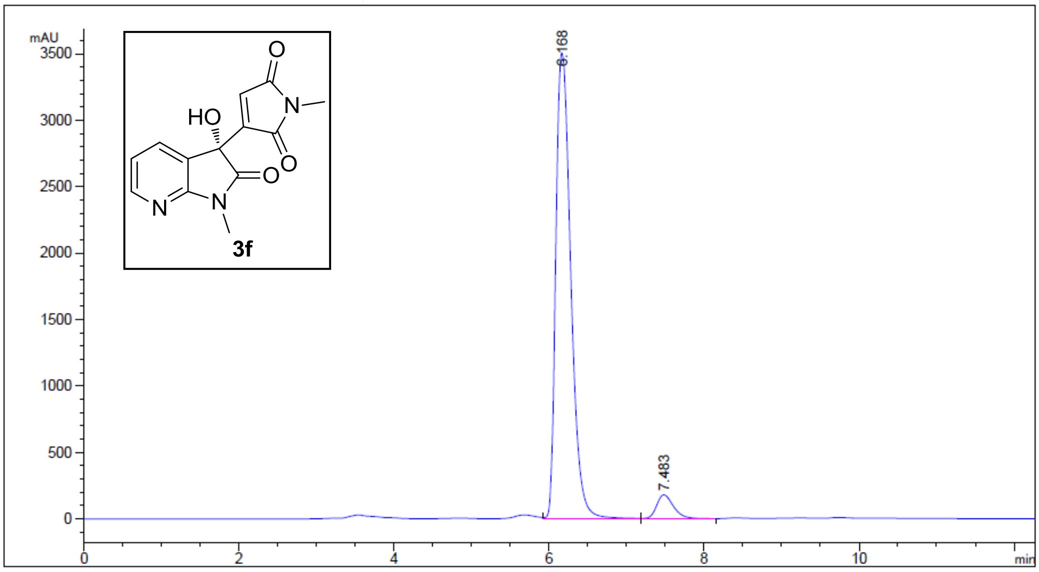

Peak #	RetTime [min]	Type	Width [min]	Area mAU *s	Height [mAU]	Area %
1	6.168	VV	0.2150	4.71425e4	3508.30396	94.3077
2	7.483	VV	0.2356	2845.48120	181.20529	5.6923

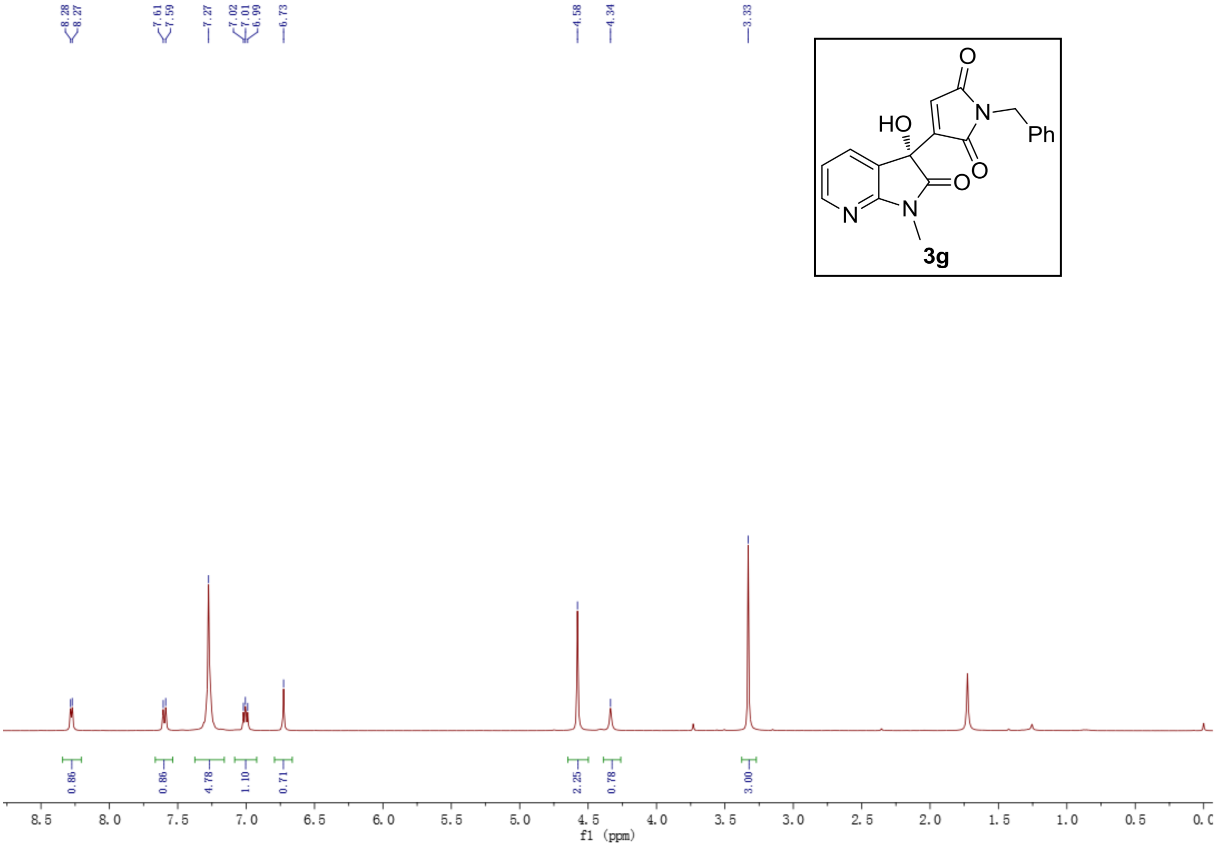
8.28
8.27
7.61
7.59
7.27
7.02
7.01
6.99
6.73
4.58
4.34
3.33
0.86
0.86
4.78
1.10
0.71
2.25
0.78
3.00
f1 (ppm)
HO
O
N
Ph
3g

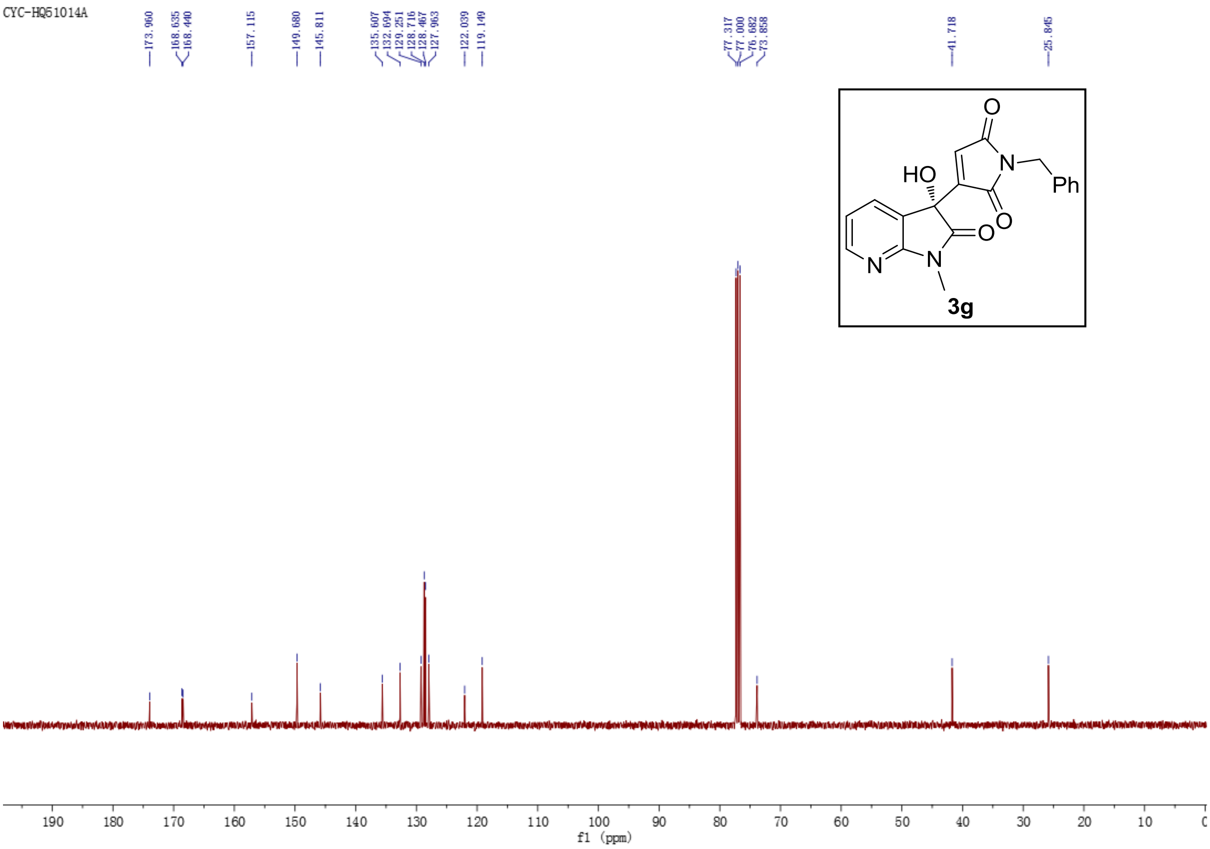
CYC-HQ51014A
173.960
168.635
168.440
157.115
149.680
145.811
135.607
132.694
129.251
128.716
128.467
127.963
122.039
119.149
77.317
77.000
76.682
73.858
41.718
25.845
f1 (ppm)
HO
O
N
Ph
3g

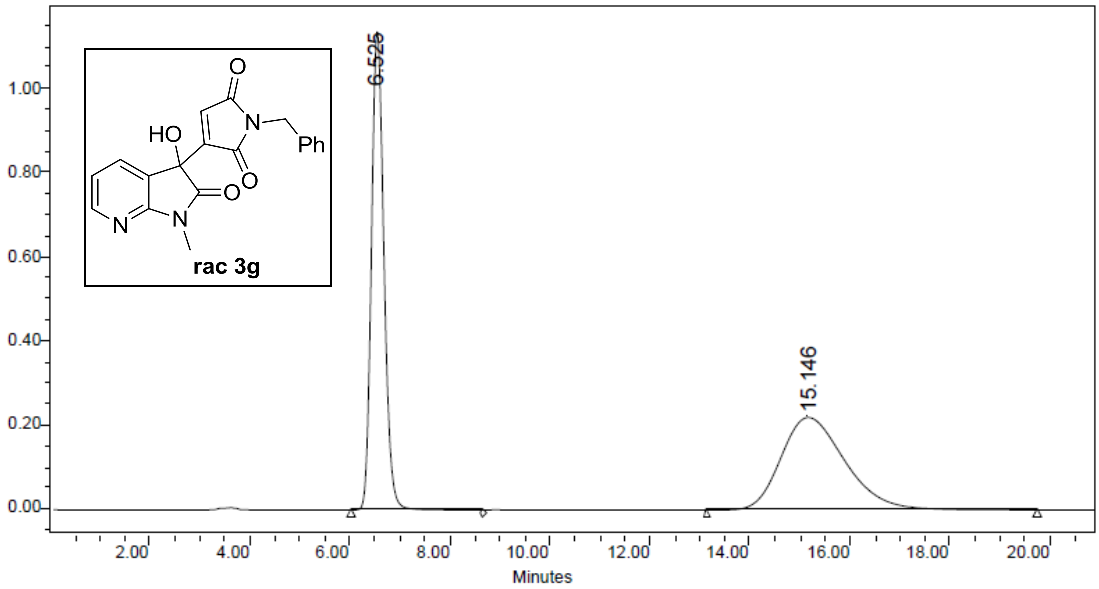

	RT (min)	Area (*sec)	% Area	Height ( )	% Height
1	6.525	19754004	50.10	1138906	83.79
2	15.146	19676964	49.90	220265	16.21

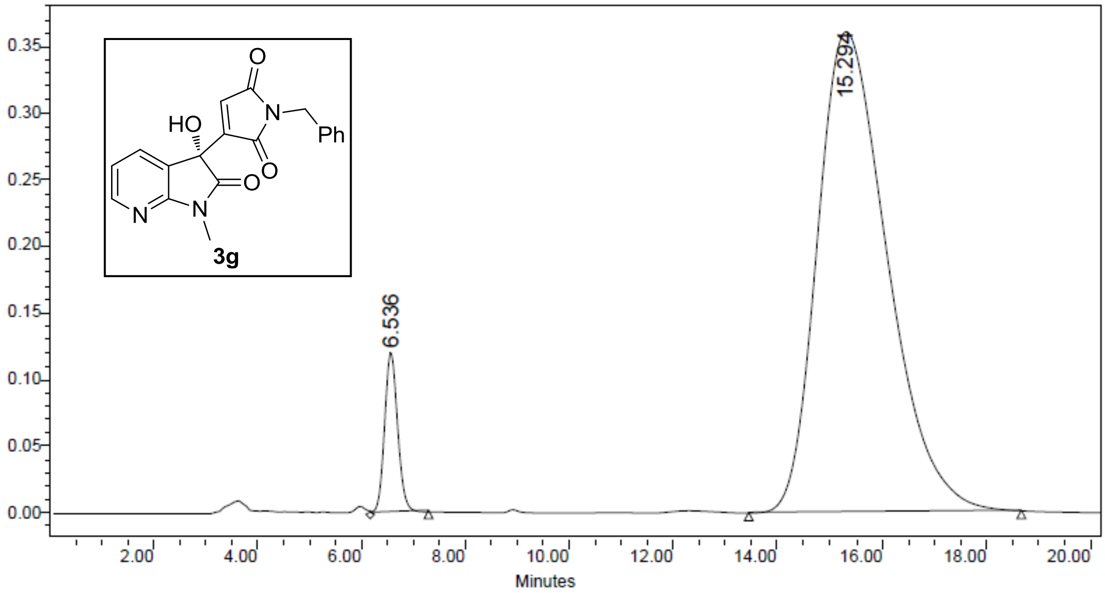

	RT (min)	Area (*sec)	% Area	Height ( )	% Height
1	6.536	2097836	5.86	119643	24.90
2	15.294	33686572	94.14	360829	75.10

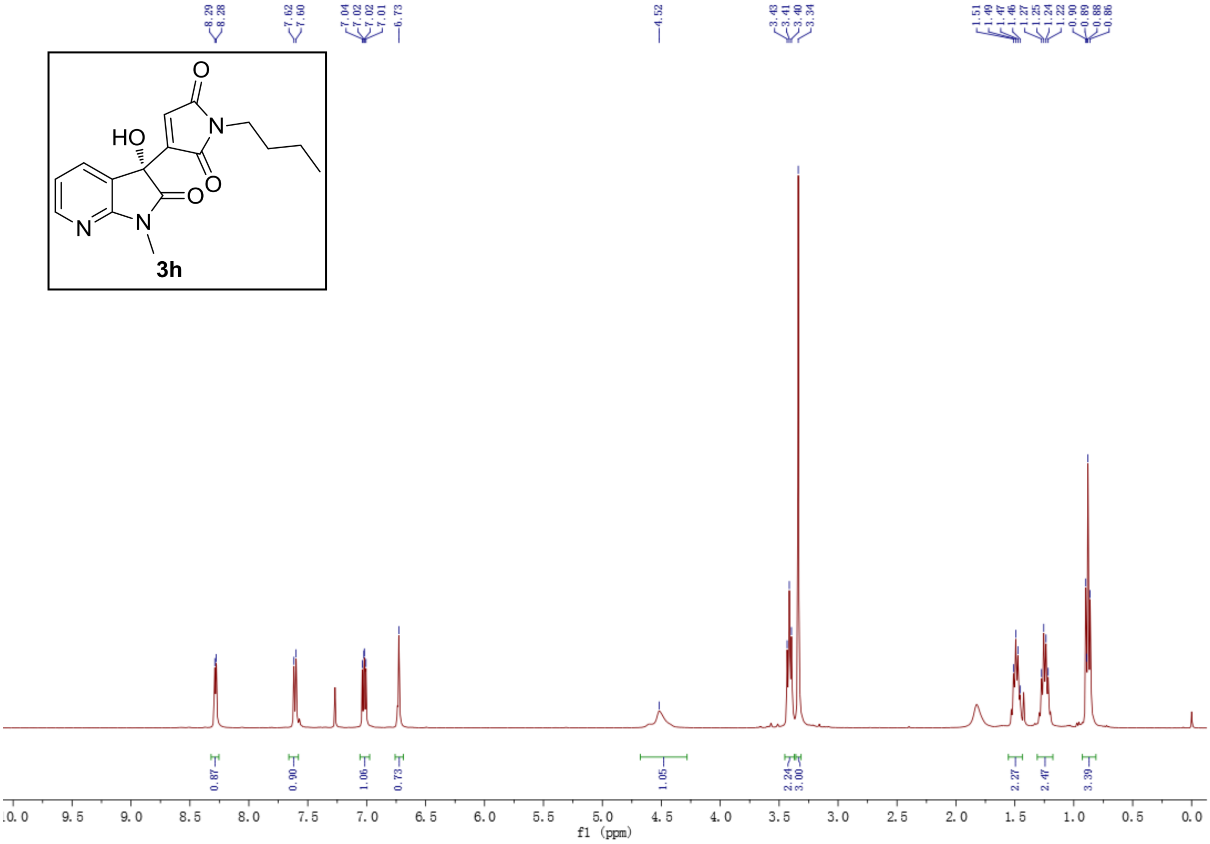

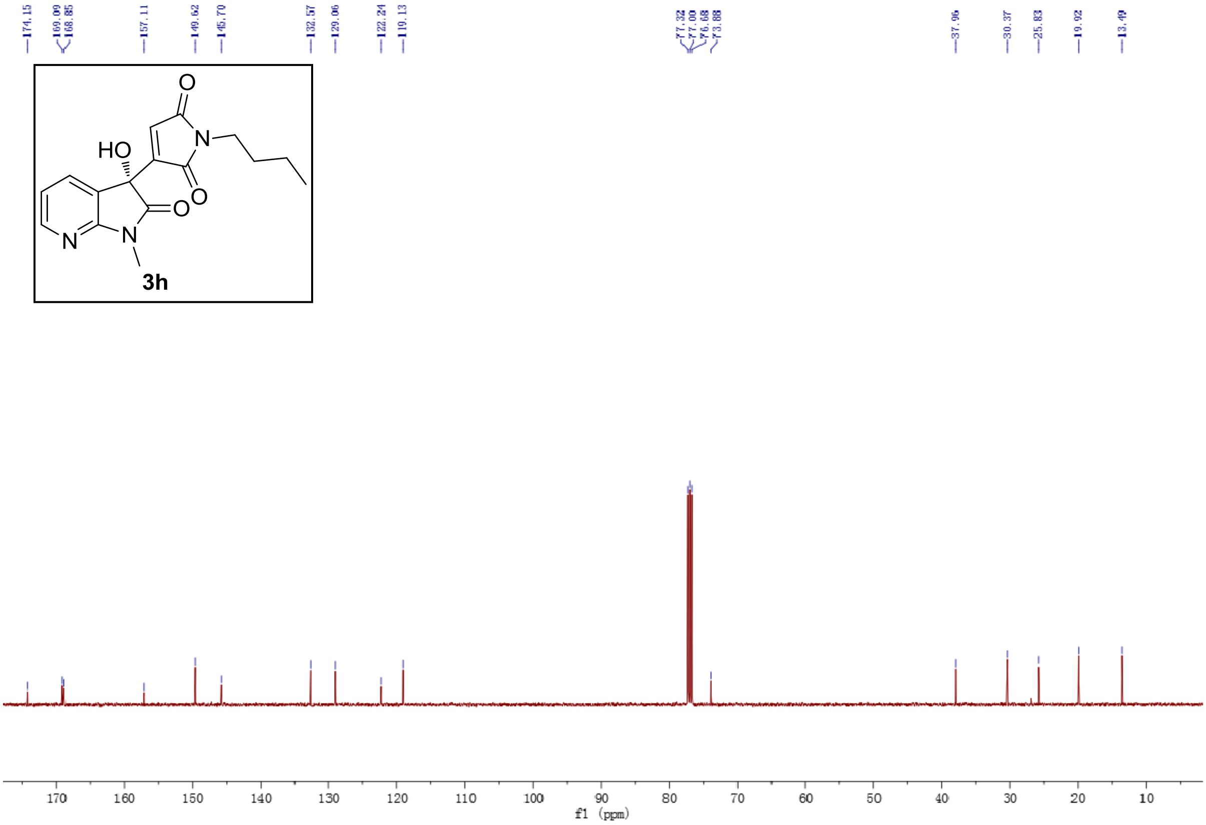

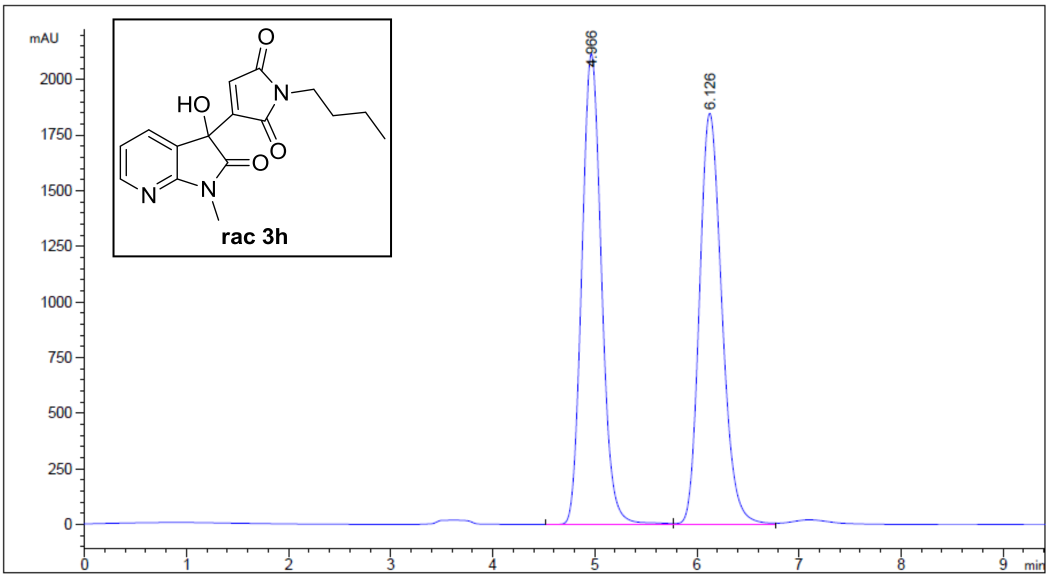

Peak #	RetTime [min]	Type	Width [min]	Area mAU *s	Height [mAU]	Area %
1	4.966	VV	0.2090	2.83408e4	2112.02271	50.0482
2	6.126	VV	0.2370	2.82862e4	1845.99268	49.9518

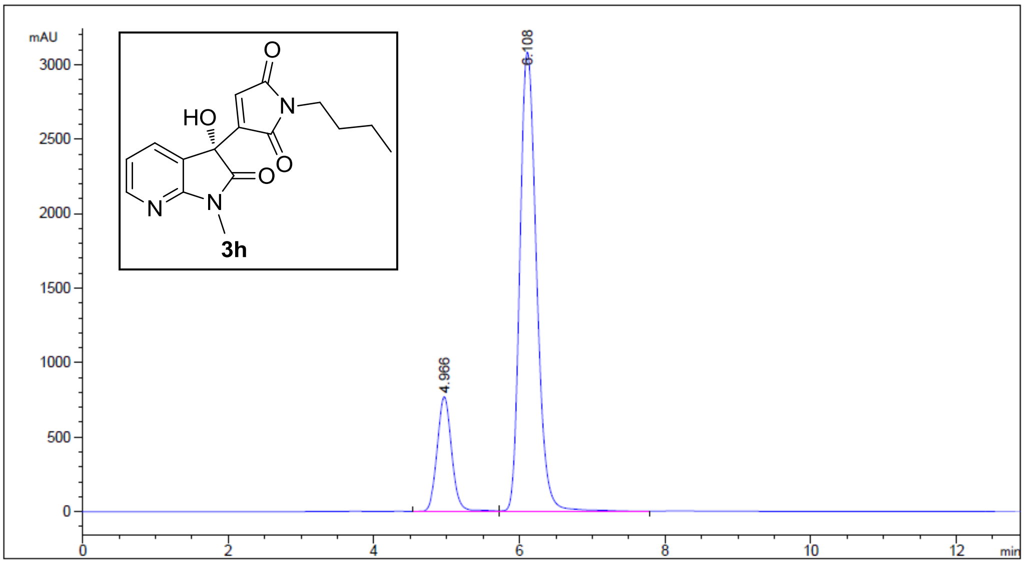

Peak #	RetTime [min]	Type	Width [min]	Area mAU *s	Height [mAU]	Area %
1	4.966	VV	0.2167	1.08344e4	769.50647	18.0121
2	6.108	VV	0.2450	4.93160e4	3080.80444	81.9879

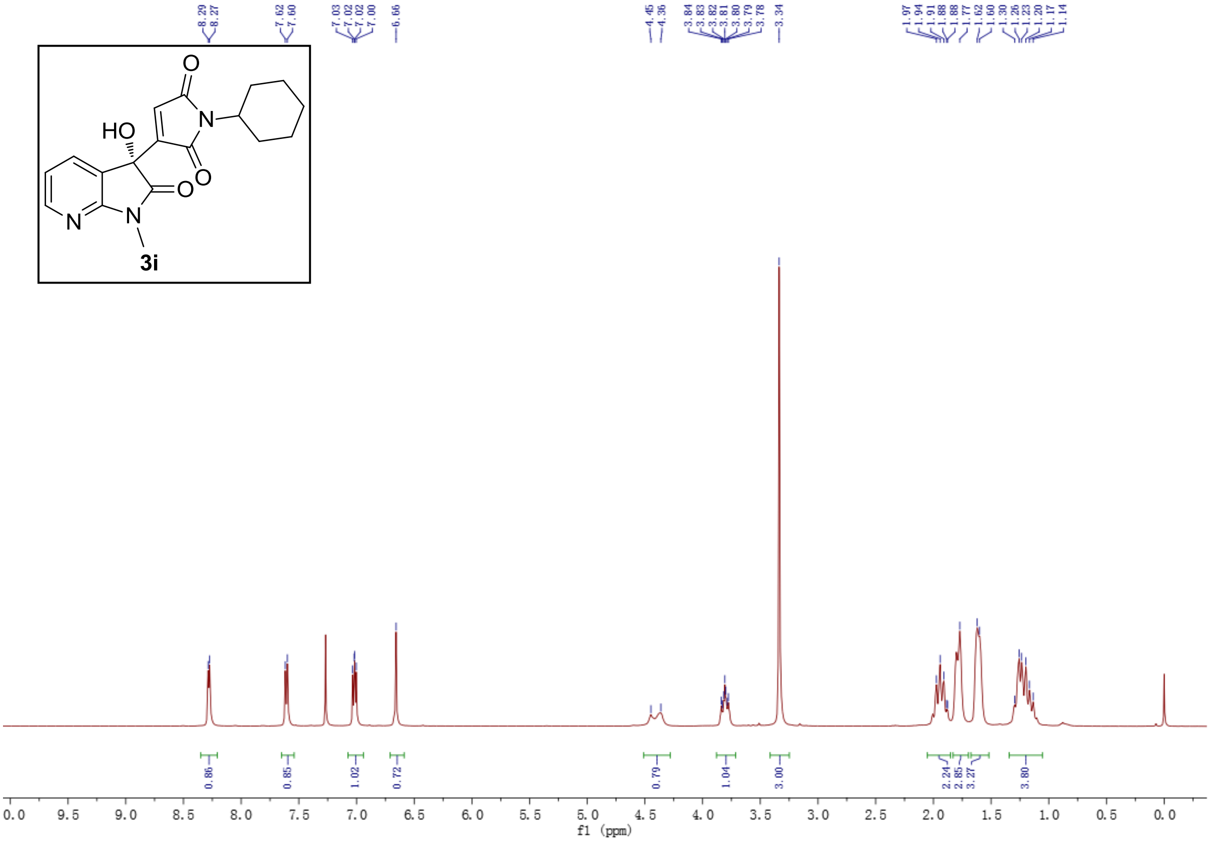
3i
HO
N
O
f1 (ppm)

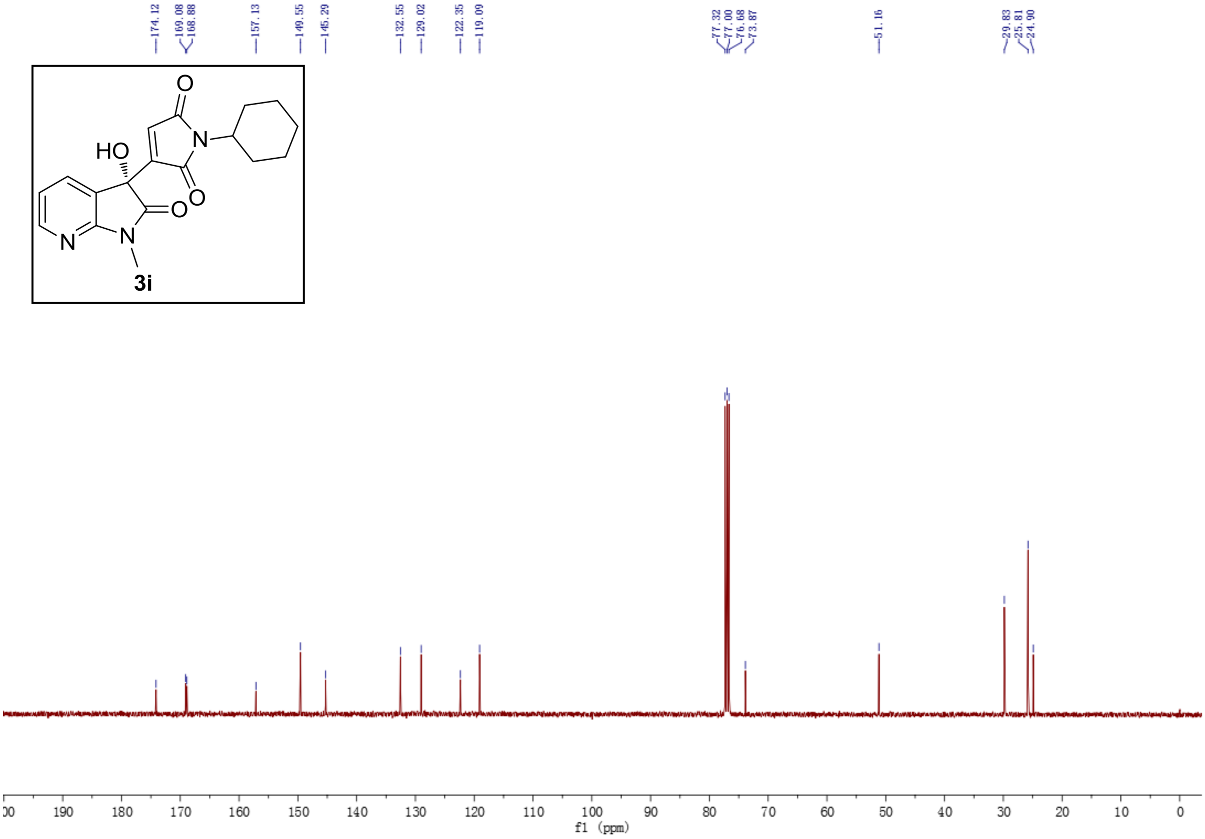
3i
HO
N
O
f1 (ppm)

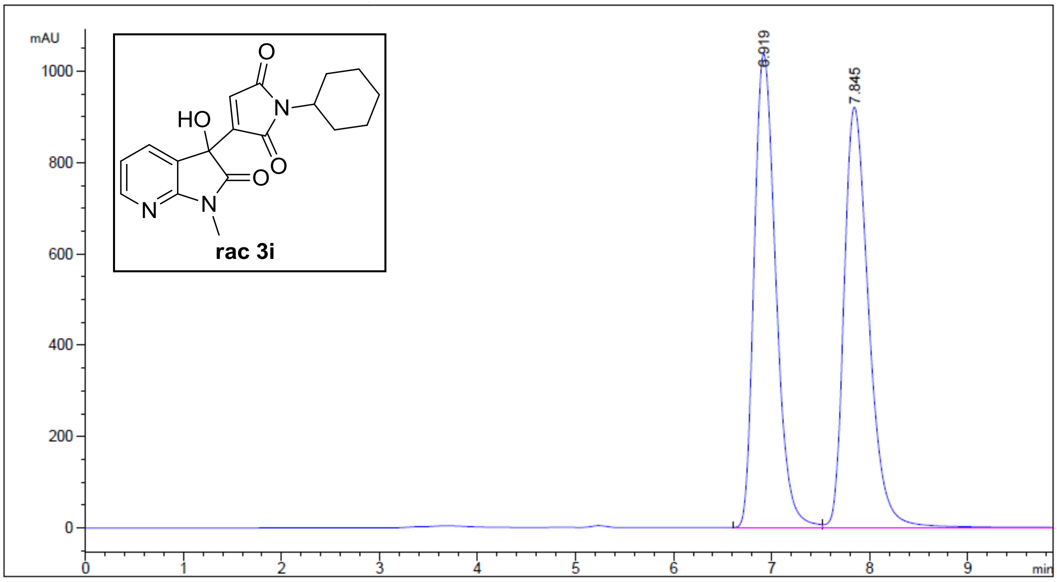

Peak #	RetTime [min]	Type	Width [min]	Area mAU *s	Height [mAU]	Area %
1	6.919	BV	0.2430	1.59147e4	1037.87170	49.5678
2	7.845	VBA	0.2693	1.61922e4	920.42444	50.4322

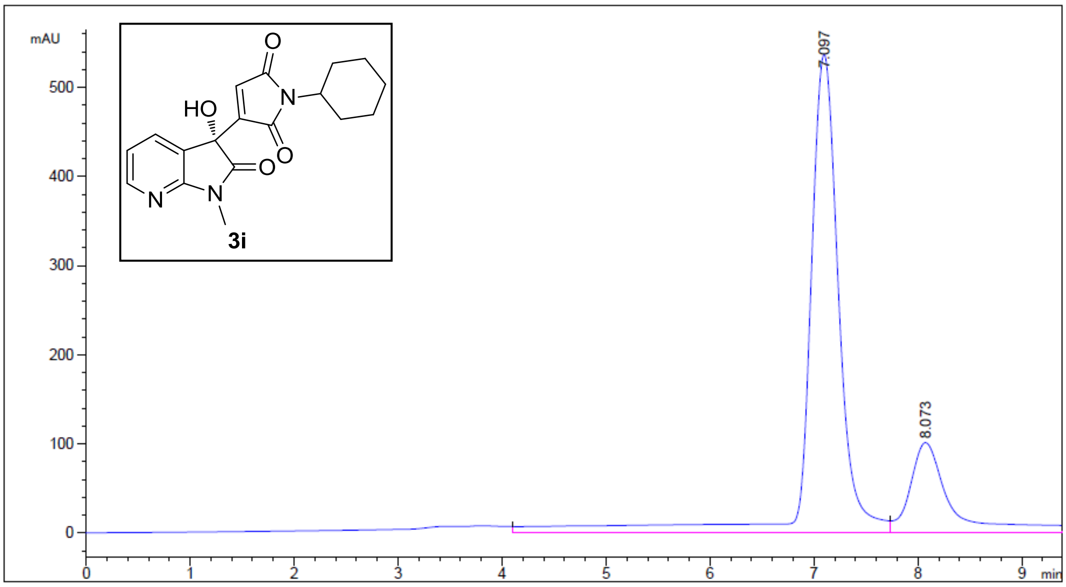

Peak #	RetTime [min]	Type	Width [min]	Area mAU *s	Height [mAU]	Area %
1	7.097	VV	0.3082	1.09523e4	536.19336	80.1268
2	8.073	VBA	0.3895	2716.41528	101.00495	19.8732

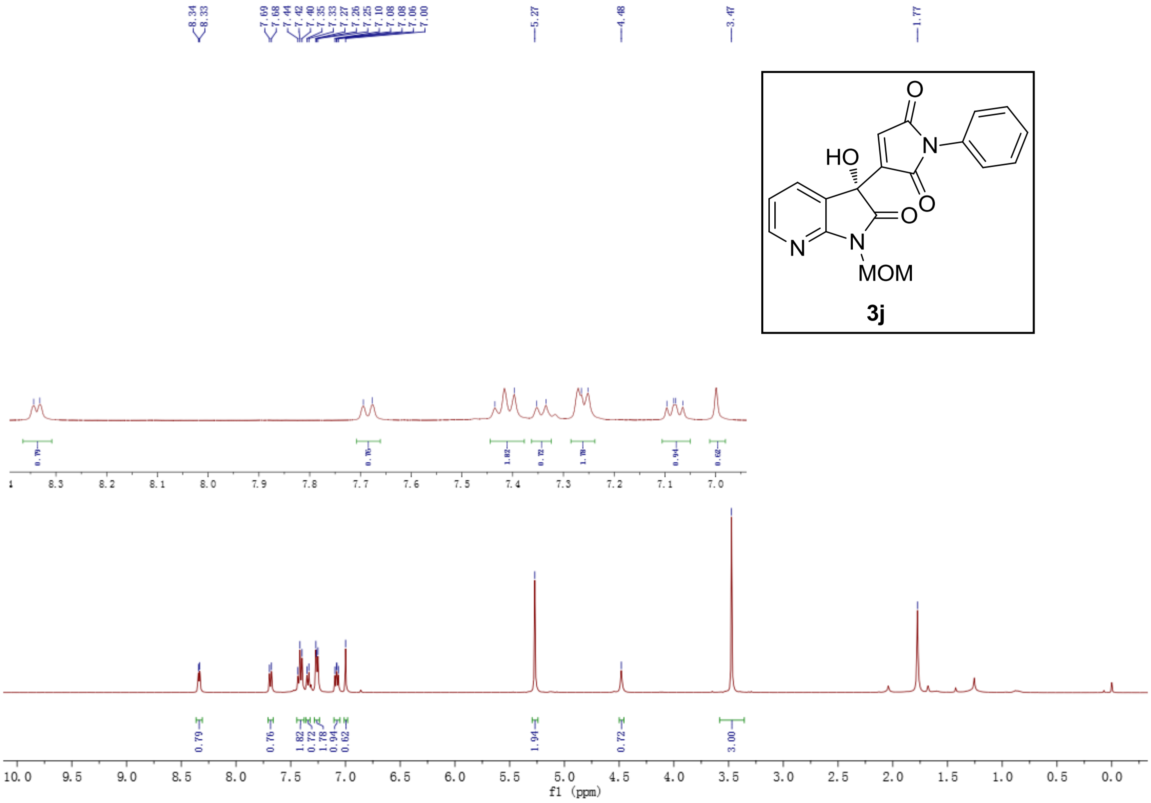
8.34
8.33
7.69
7.68
7.44
7.42
7.40
7.35
7.33
7.27
7.26
7.25
7.10
7.08
7.06
7.00
5.27
4.48
3.47
1.77
HO
O
N
MOM
3j
0.79
0.76
1.82
0.72
1.78
0.94
0.62
1.94
0.72
3.00
f1 (ppm)

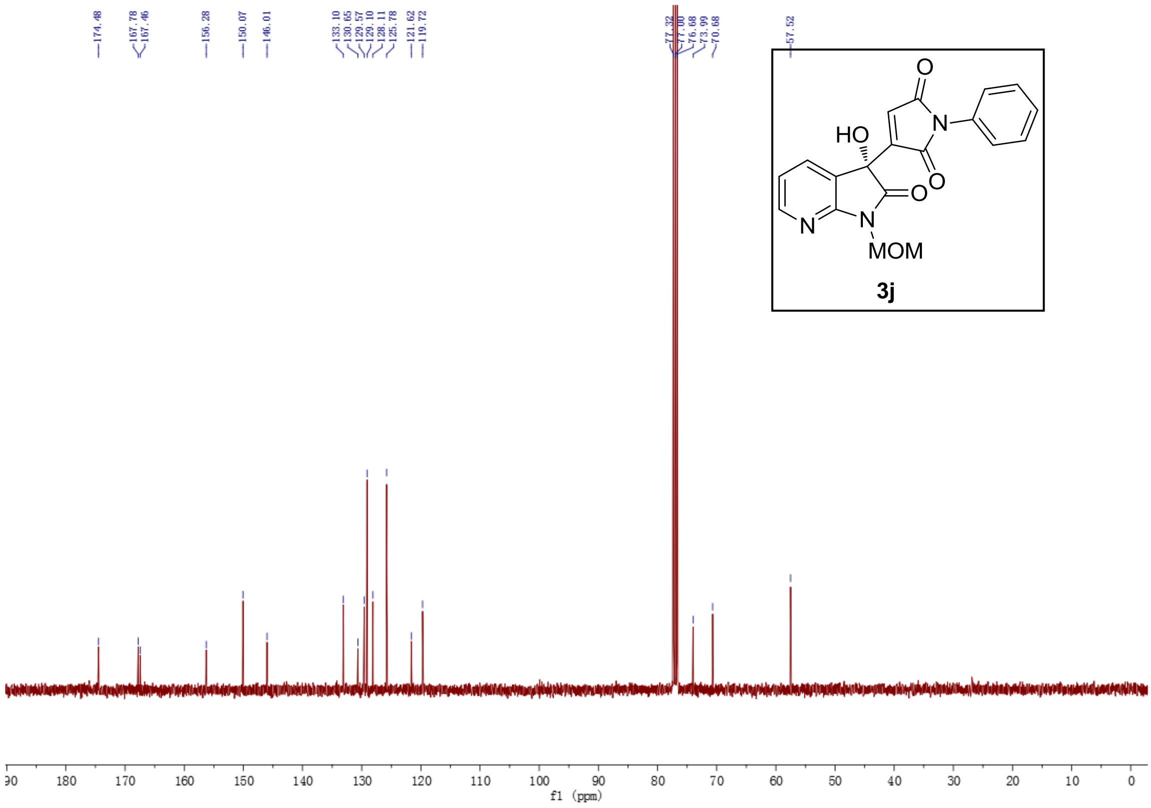
174.48
167.78
167.46
156.28
150.07
146.01
133.10
130.65
129.57
129.10
128.11
125.78
121.62
119.72
77.32
77.00
76.68
73.99
70.68
57.52
HO
O
N
MOM
3j
f1 (ppm)

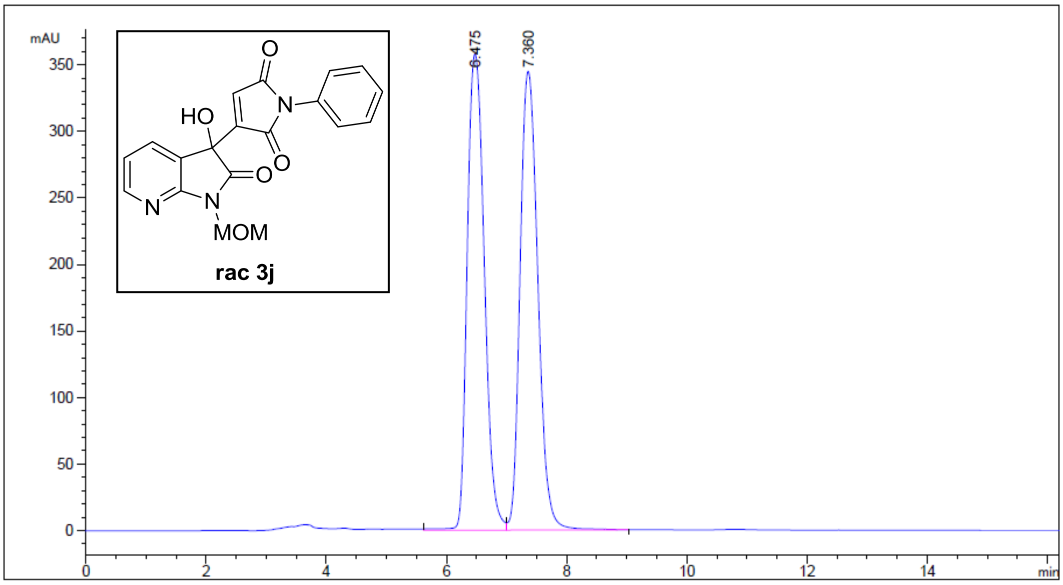

Peak #	RetTime [min]	Type	Width [min]	Area mAU *s	Height [mAU]	Area %
1	6.475	BV	0.3180	7070.14600	357.59406	49.9557
2	7.360	VB	0.3273	7082.67432	344.38330	50.0443

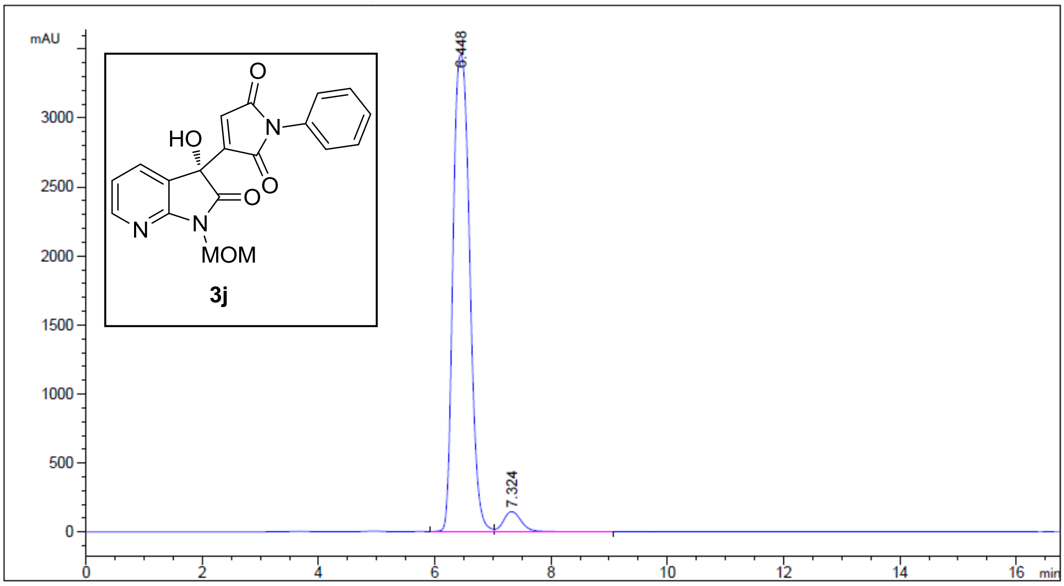

Peak #	RetTime [min]	Type	Width [min]	Area mAU *s	Height [mAU]	Area %
1	6.448	VV	0.3188	6.86286e4	3460.13452	95.4869
2	7.324	VB	0.3463	3243.63623	146.11922	4.5131

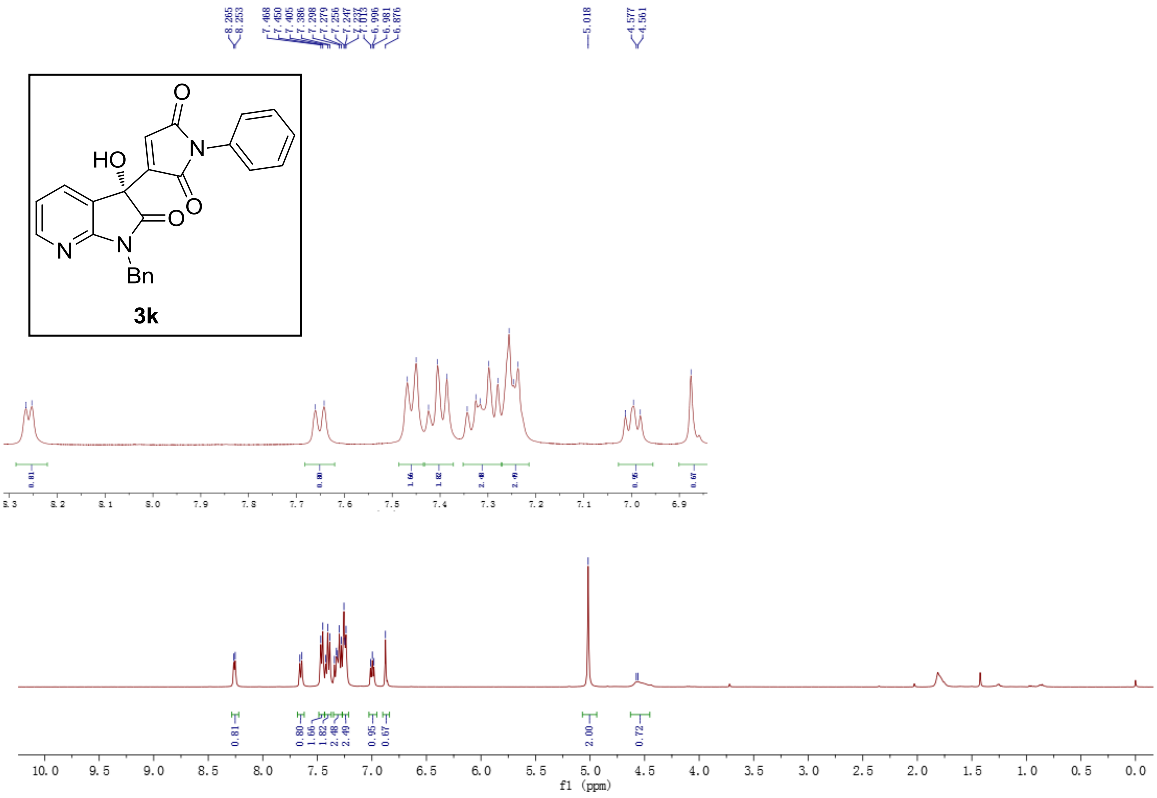
8.265
8.253
7.468
7.450
7.405
7.386
7.298
7.279
7.256
7.247
7.237
7.013
6.996
6.981
6.876
5.018
4.577
4.561
HO
O
N
O
O
N
N
Bn
3k
0.81
0.80
1.66
1.82
2.48
2.49
0.95
0.67
2.00
0.72
f1 (ppm)

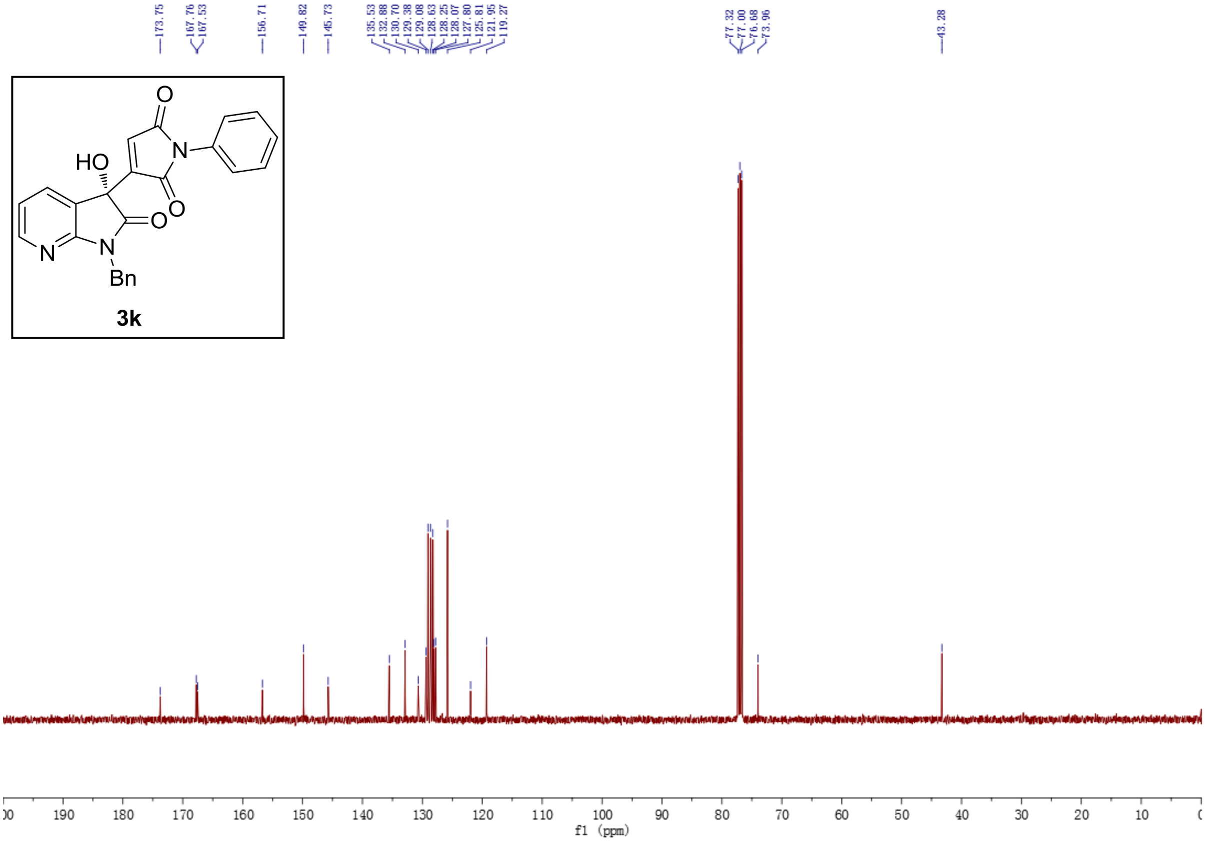
173.75
167.76
167.53
156.71
149.82
145.73
135.53
132.88
130.70
129.38
129.08
128.63
128.25
128.07
127.80
125.81
121.95
119.27
77.32
77.00
76.68
73.96
43.28
HO
O
N
O
O
N
N
Bn
3k
f1 (ppm)

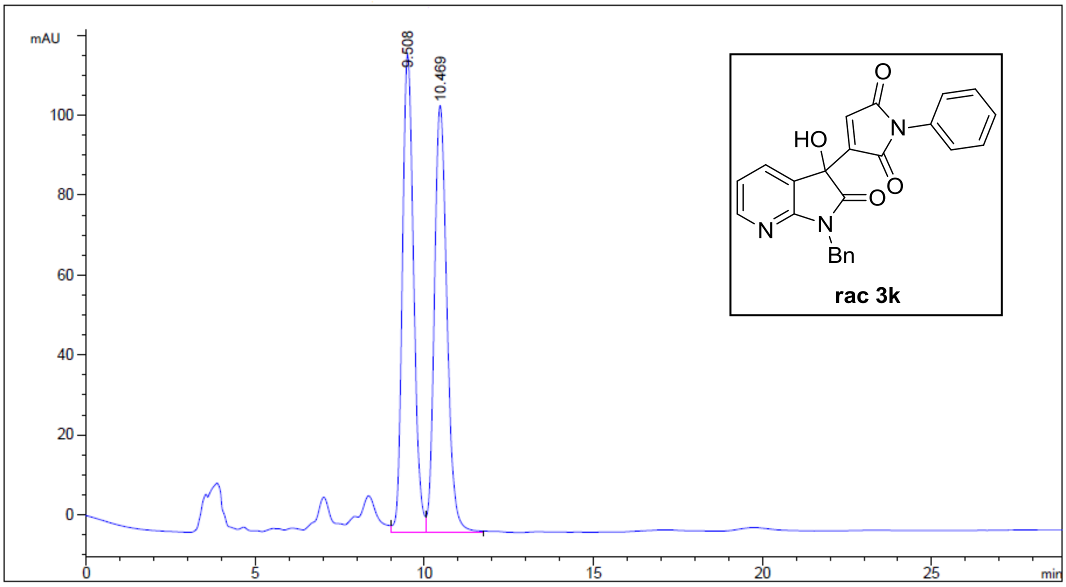

Peak #	RetTime [min]	Type	Width [min]	Area mAU *s	Height [mAU]	Area %
1	9.508	VV	0.3626	2759.91113	119.53596	50.1232
2	10.469	VB	0.3993	2746.34204	106.69016	49.8768

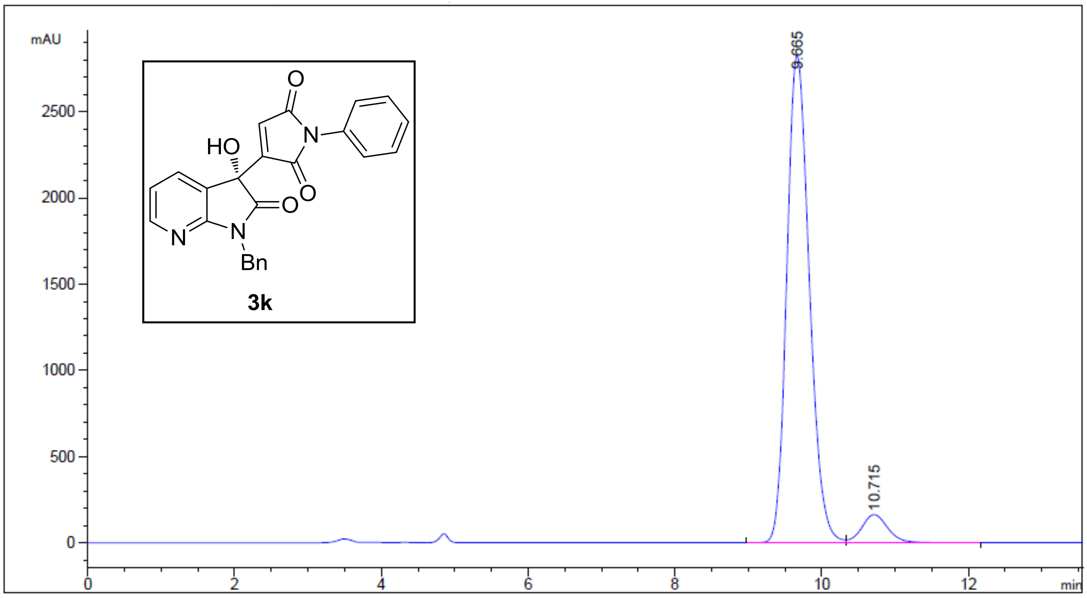

Peak #	RetTime [min]	Type	Width [min]	Area mAU *s	Height [mAU]	Area %
1	9.665	VV	0.3299	6.01519e4	2825.41870	93.7241
2	10.715	VB	0.3824	4027.87354	162.50554	6.2759

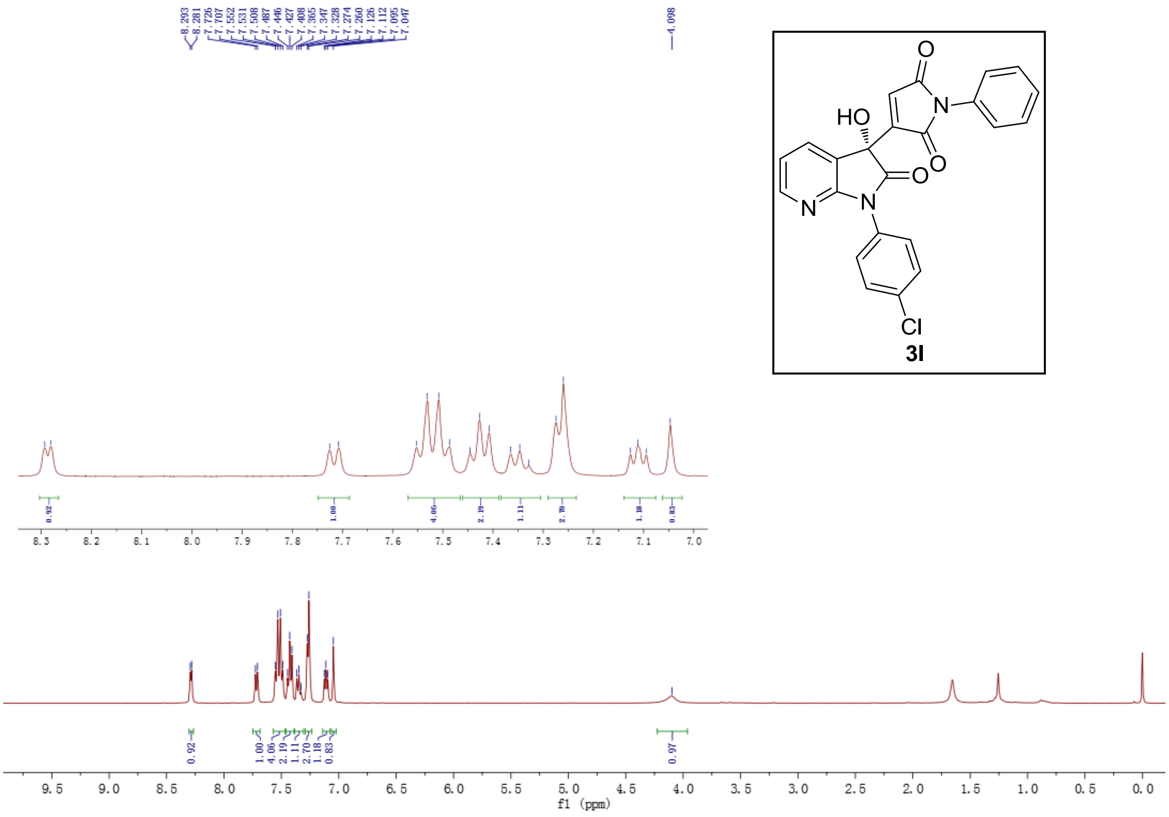
8.293
8.281
7.726
7.707
7.552
7.531
7.508
7.487
7.446
7.427
7.408
7.365
7.347
7.328
7.274
7.260
7.126
7.112
7.095
7.047
4.098
0.92
1.00
4.06
2.19
1.11
2.70
1.18
0.83
0.97
f1 (ppm)
O
HO
N
O
O
N
N
Cl

3l

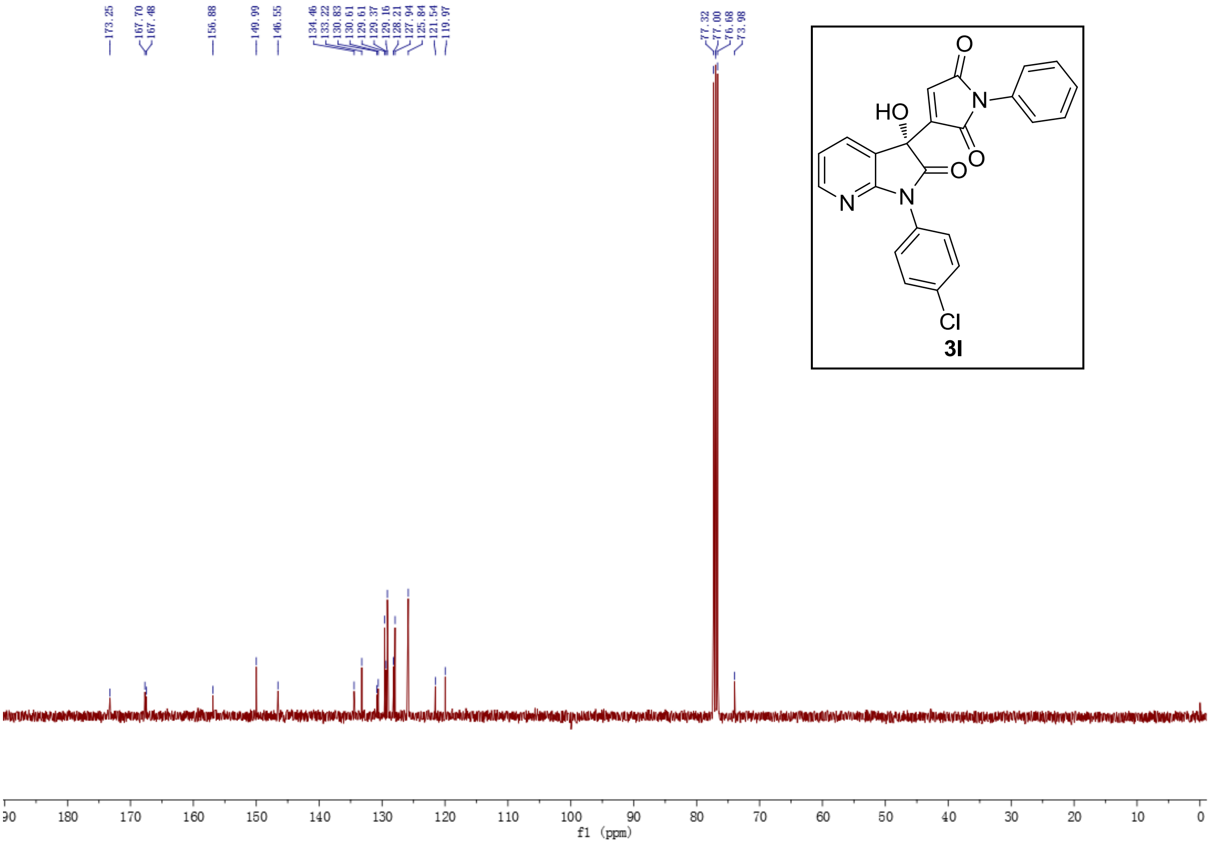
173.25
167.70
167.48
156.88
149.99
146.55
134.46
133.22
130.83
130.61
129.61
129.37
129.16
128.21
127.94
125.84
121.54
119.97
77.32
77.00
76.68
73.98
f1 (ppm)
O
HO
N
O
O
N
N
Cl

3l

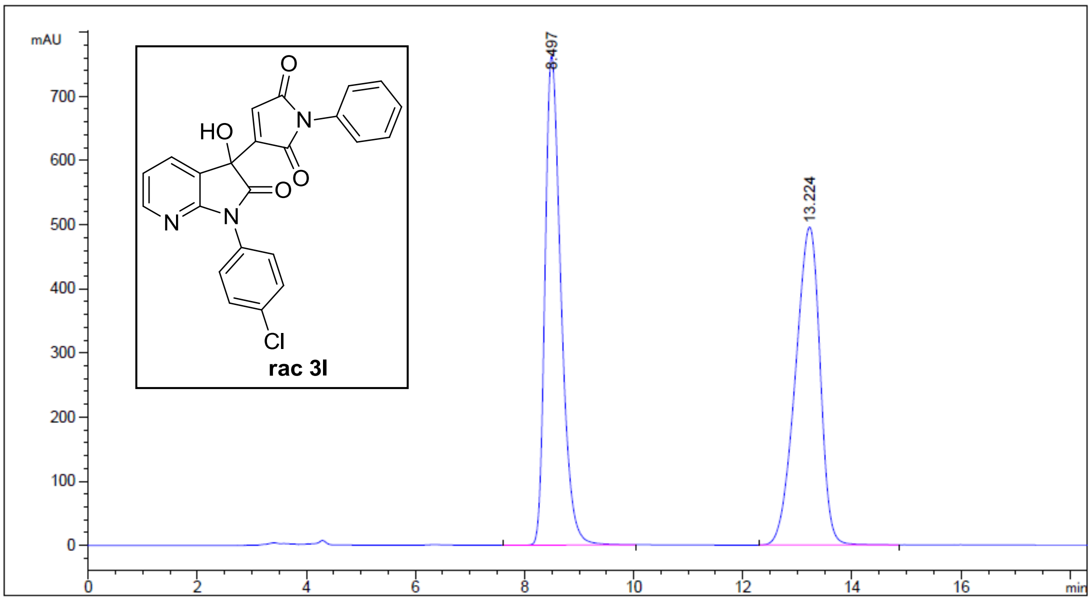

Peak #	RetTime [min]	Type	Width [min]	Area mAU *s	Height [mAU]	Area %
1	8.497	BB	0.3072	1.55036e4	762.28271	49.9124
2	13.224	BB	0.4888	1.55580e4	495.27643	50.0876

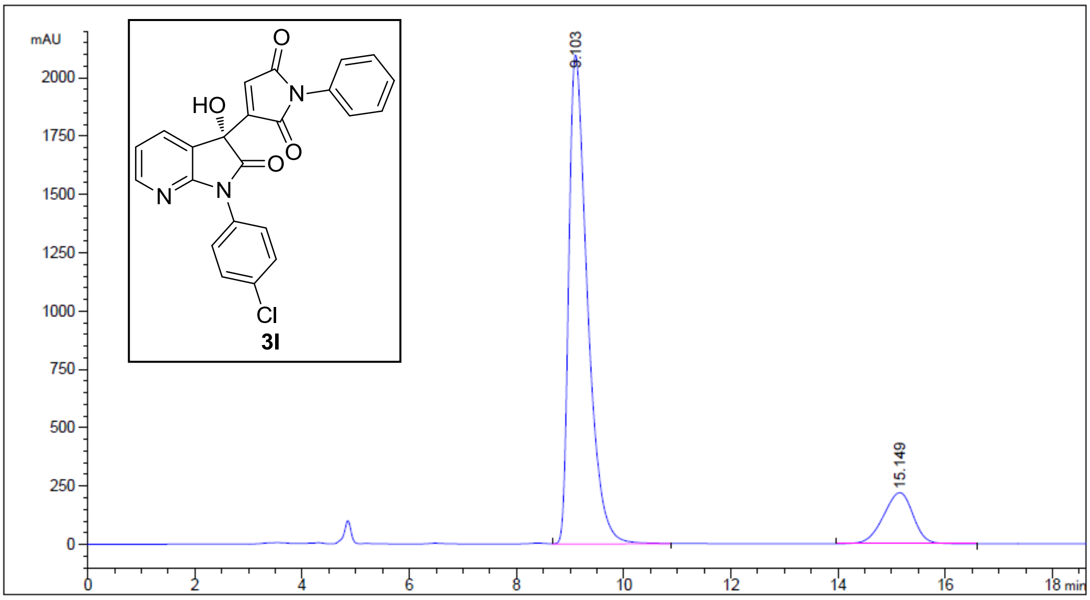

Peak #	RetTime [min]	Type	Width [min]	Area mAU *s	Height [mAU]	Area %
1	9.103	VV	0.3634	5.05777e4	2093.53223	85.6904
2	15.149	BB	0.6009	8446.07617	219.22653	14.3096

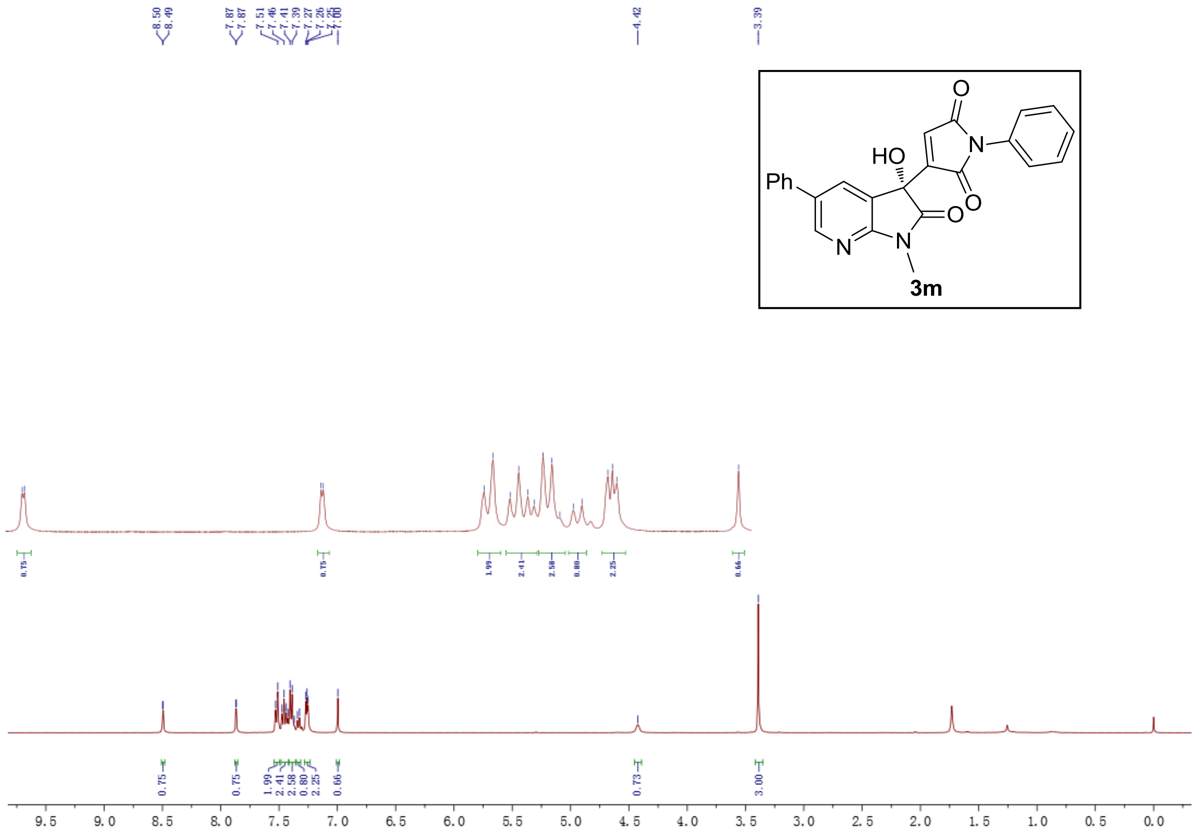
8.50
8.49
7.87
7.87
7.51
7.46
7.41
7.39
7.27
7.26
4.42
3.39
Ph
HO
N
O
3m

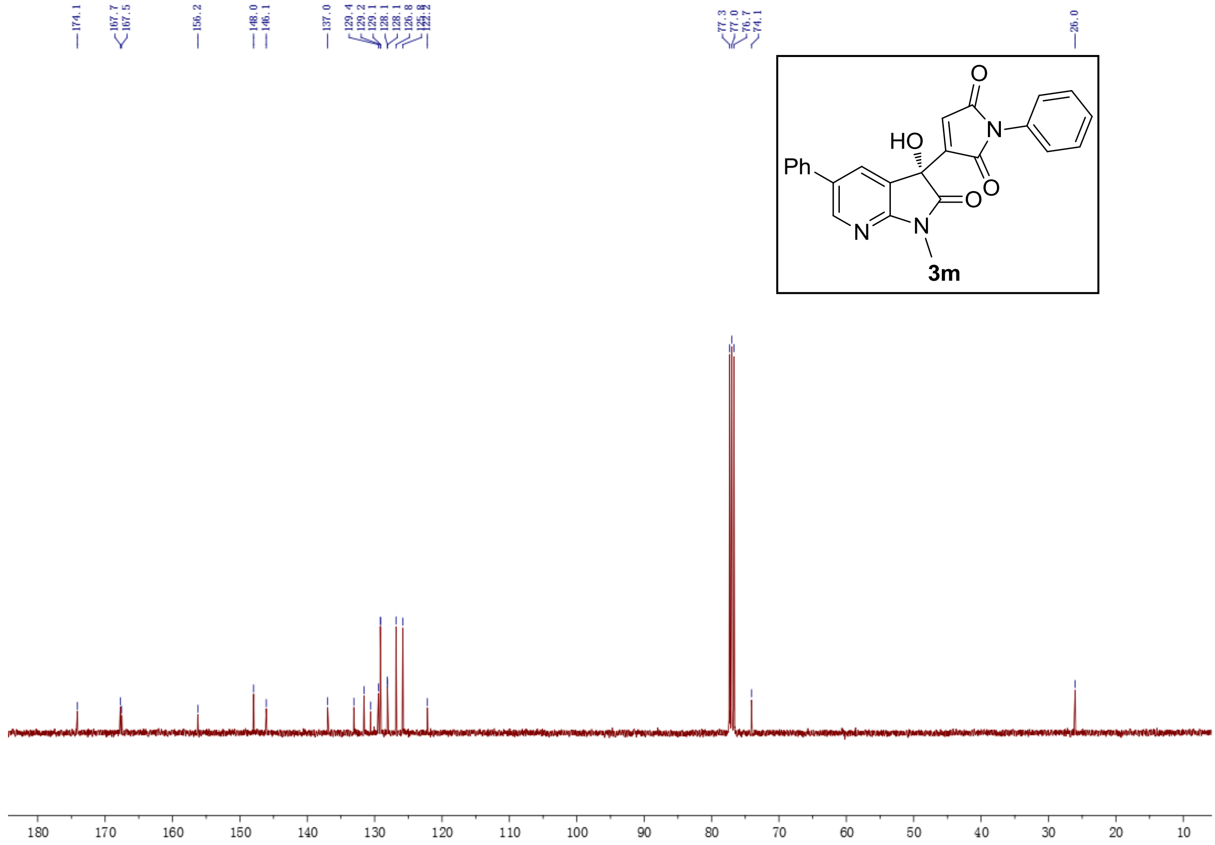
174.1
167.7
167.5
156.2
148.0
146.1
137.0
77.3
77.0
76.7
74.1
26.0
Ph
HO
N
O
3m

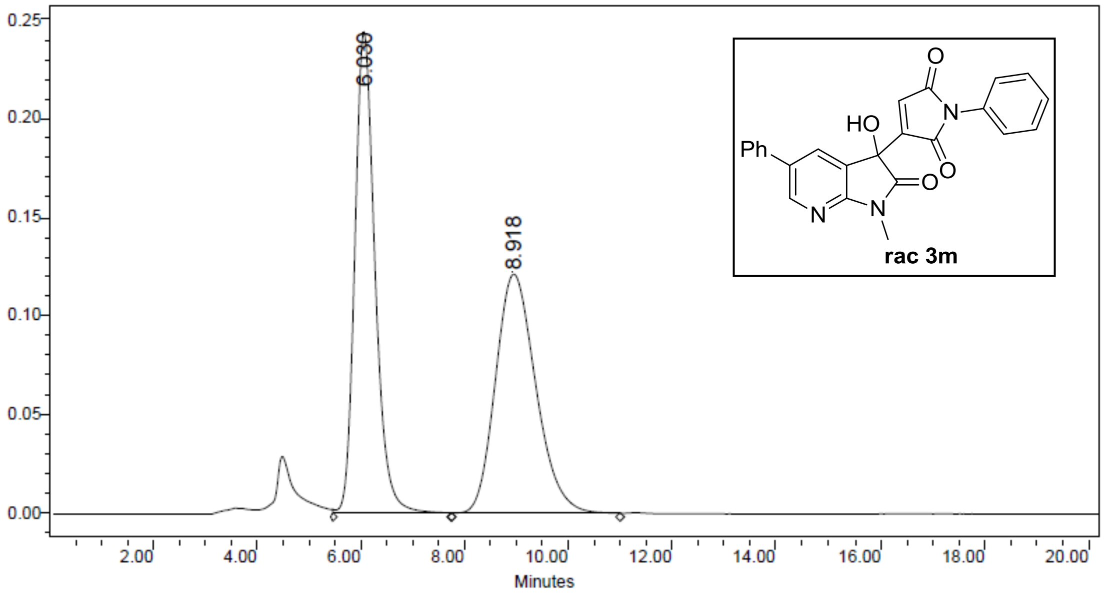

	RT (min)	Area (*sec)	% Area	Height ( )	% Height
1	6.030	6658183	50.17	244390	66.78
2	8.918	6613900	49.83	121564	33.22

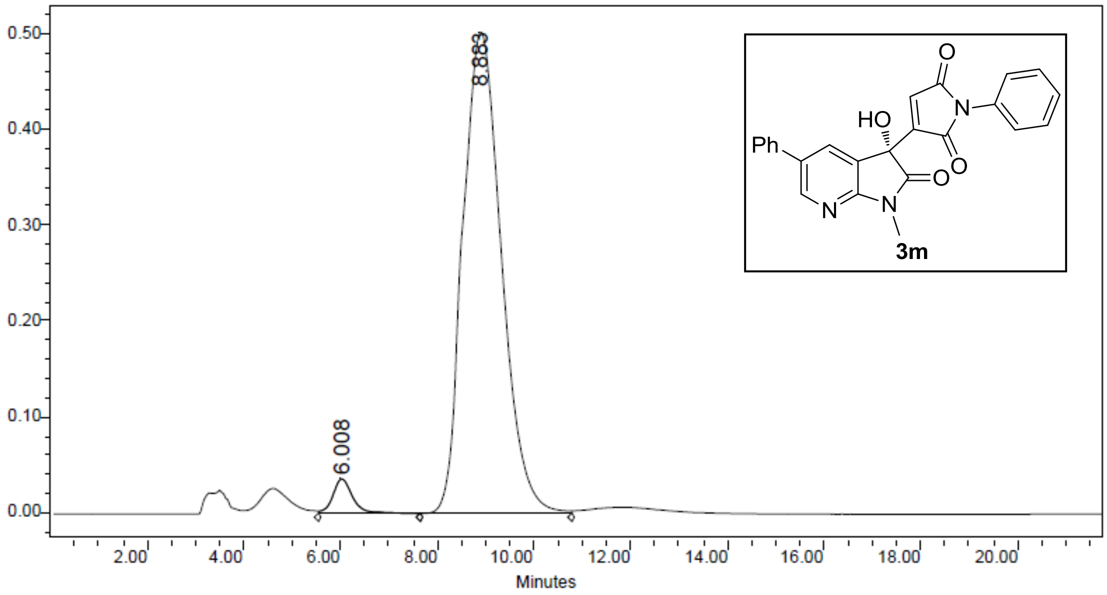

	RT (min)	Area (*sec)	% Area	Height ( )	% Height
1	6.008	1123134	3.86	36691	6.80
2	8.883	27946419	96.14	502897	93.20

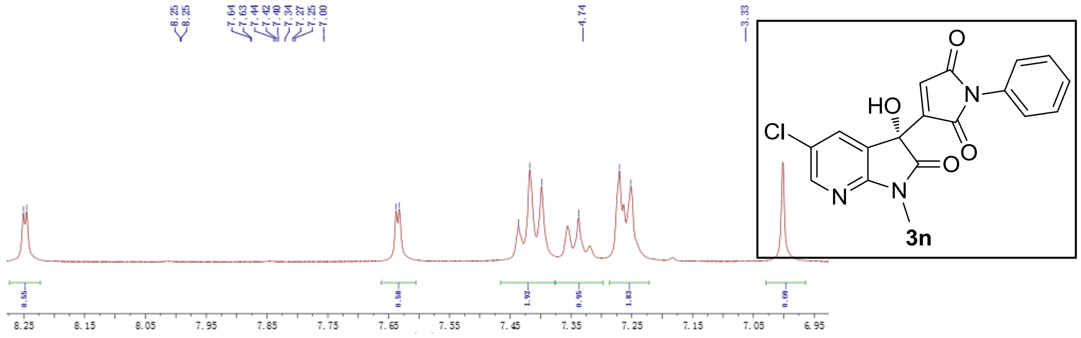
8.25
8.25
7.64
7.63
7.44
7.42
7.40
7.34
7.27
7.25
7.00
4.74
3.33
HO
Cl
O
N
O
O
N
N
3n
0.55
0.58
1.92
0.95
1.83
0.69

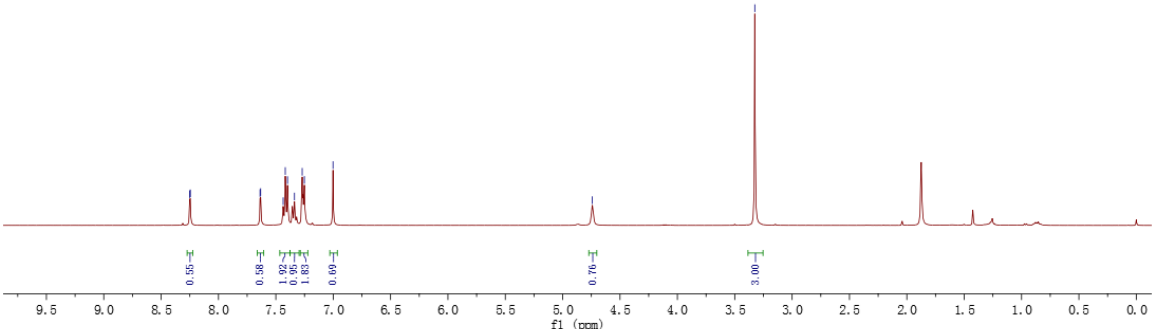
0.55
0.58
1.92
0.95
1.83
0.69
0.76
3.00
f1 (ppm)

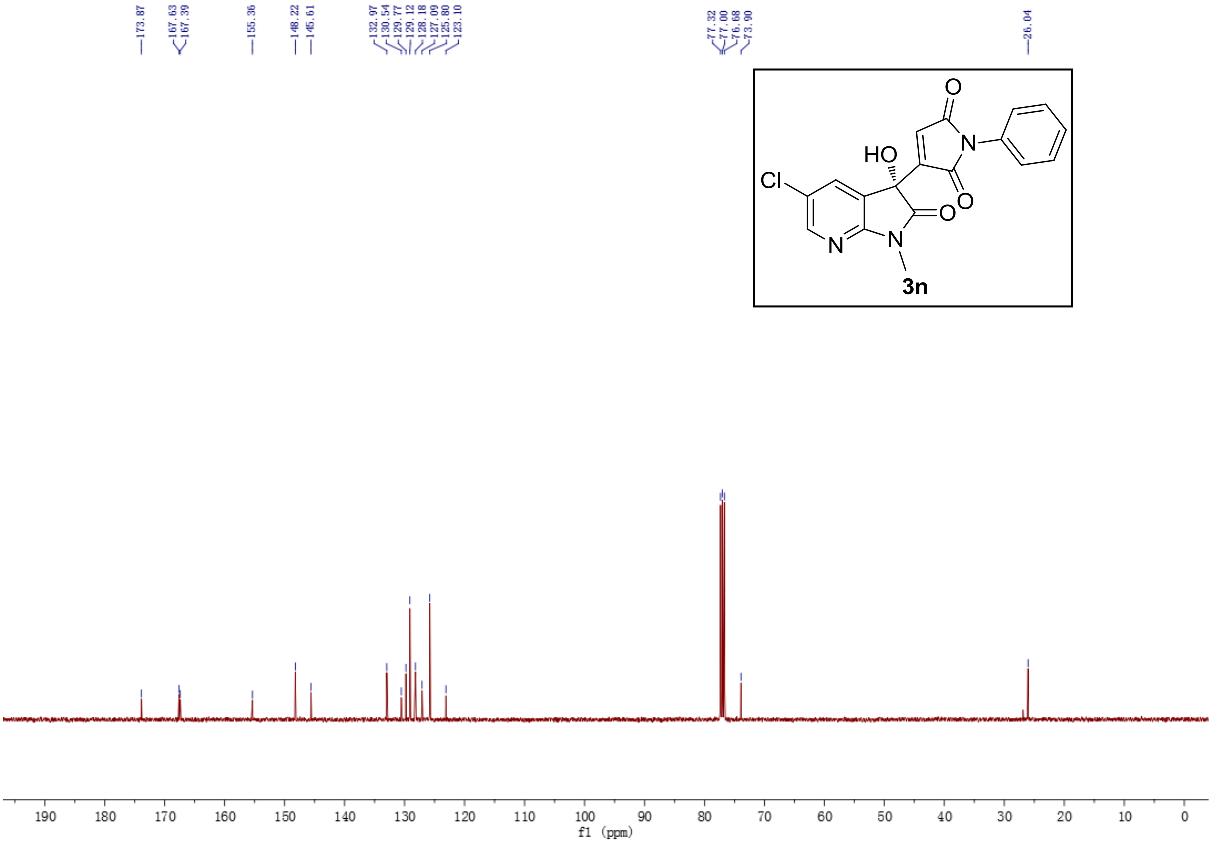
173.87
167.63
167.39
155.36
148.22
145.61
132.97
130.54
129.77
129.12
128.18
127.09
125.80
123.10
77.32
77.00
76.68
73.90
26.04
HO
Cl
O
N
O
O
N
N
3n
f1 (ppm)

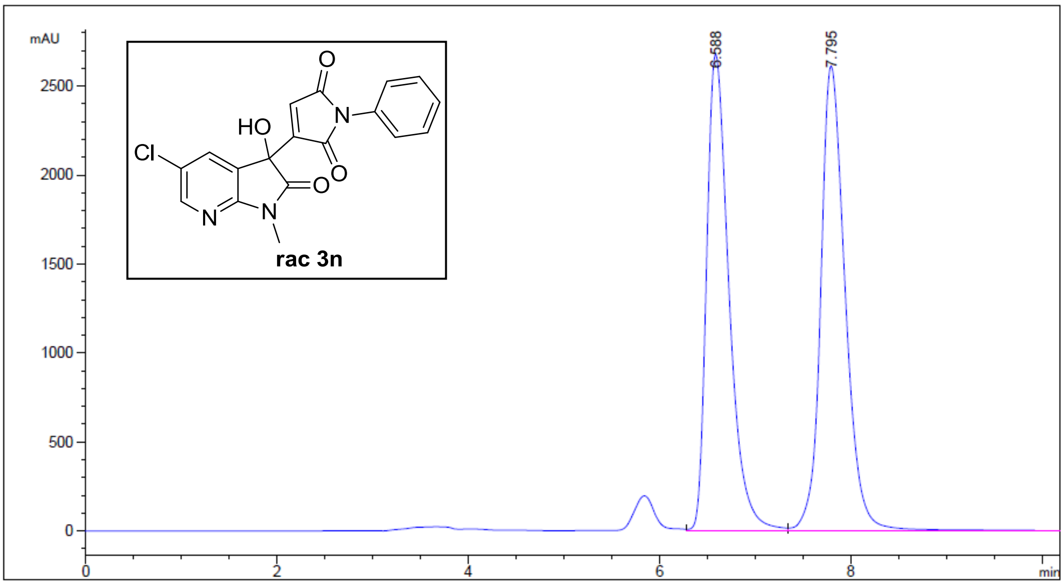

Peak #	RetTime [min]	Type	Width [min]	Area mAU *s	Height [mAU]	Area %
1	6.588	VV	0.2542	4.35827e4	2674.33325	48.2507
2	7.795	VBA	0.2730	4.67428e4	2609.88525	51.7493

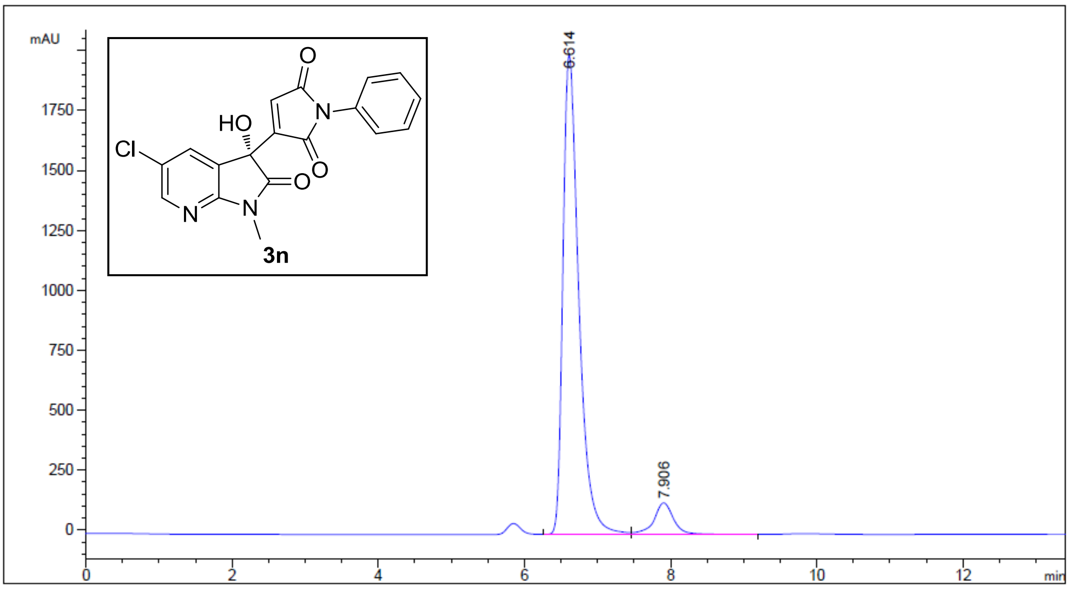

Peak #	RetTime [min]	Type	Width [min]	Area mAU *s	Height [mAU]	Area %
1	6.614	VV	0.2343	3.01889e4	2000.41687	92.3205
2	7.906	VV	0.2861	2511.18506	131.96326	7.6795

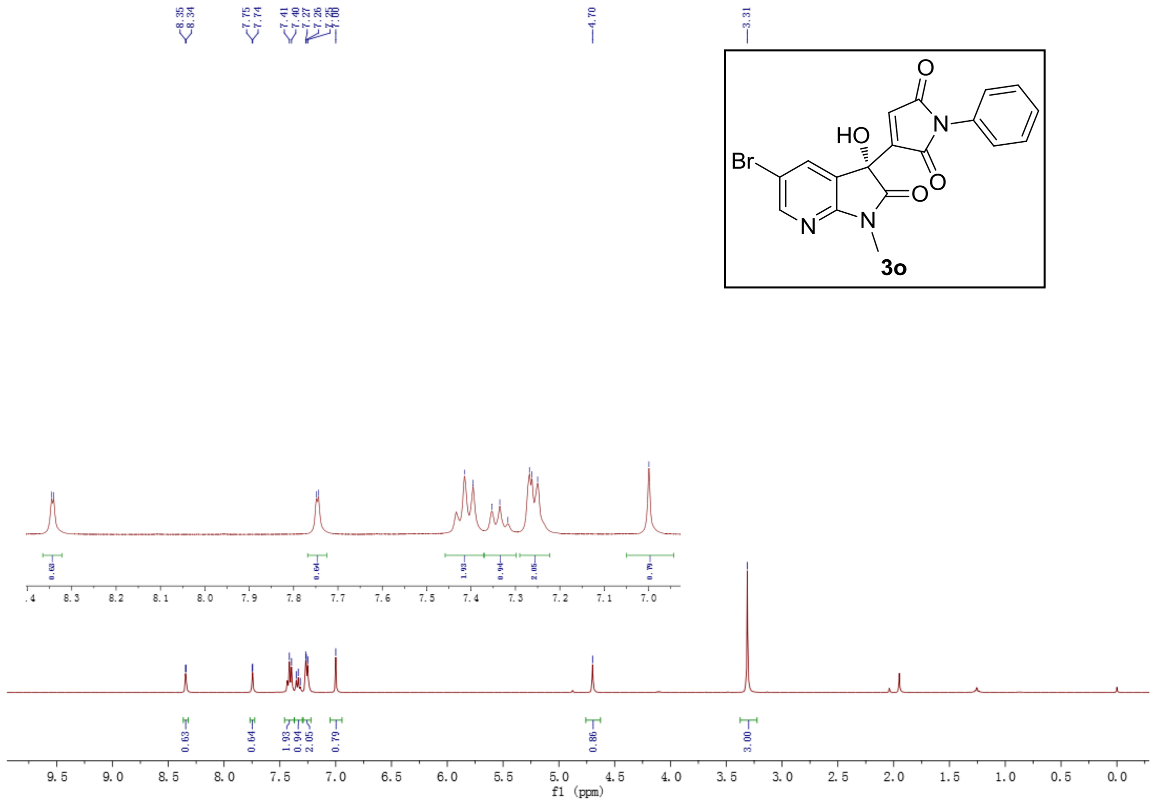
8.35
8.34
7.75
7.74
7.41
7.40
7.27
7.26
7.00
4.70
3.31
Br
HO
N
O
0.63
0.64
1.93
0.94
2.05
0.79
0.86
3.00
f1 (ppm)

3o

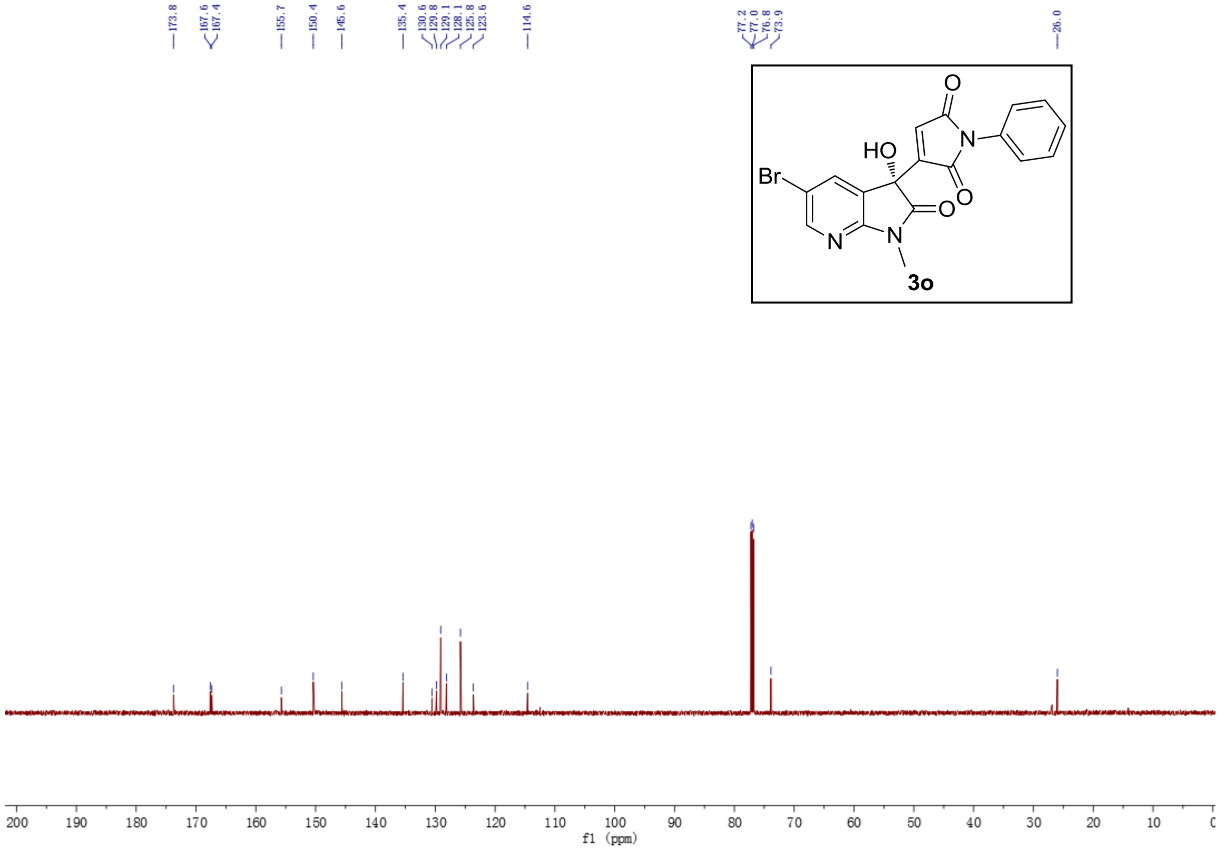
173.8
167.6
167.4
155.7
150.4
145.6
135.4
130.6
129.8
129.1
128.1
125.8
123.6
114.6
77.2
77.0
76.8
73.9
26.0
Br
HO
N
O
f1 (ppm)

3o

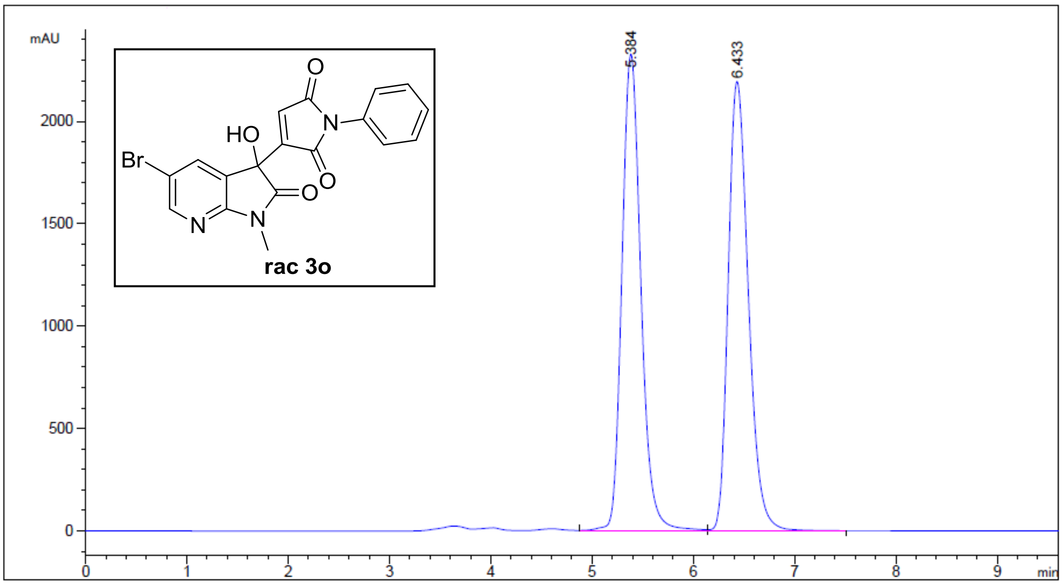

Peak #	RetTime [min]	Type	Width [min]	Area mAU *s	Height [mAU]	Area %
1	5.384	VV	0.2051	3.04184e4	2324.98242	50.0584
2	6.433	VV	0.2139	3.03474e4	2192.14185	49.9416

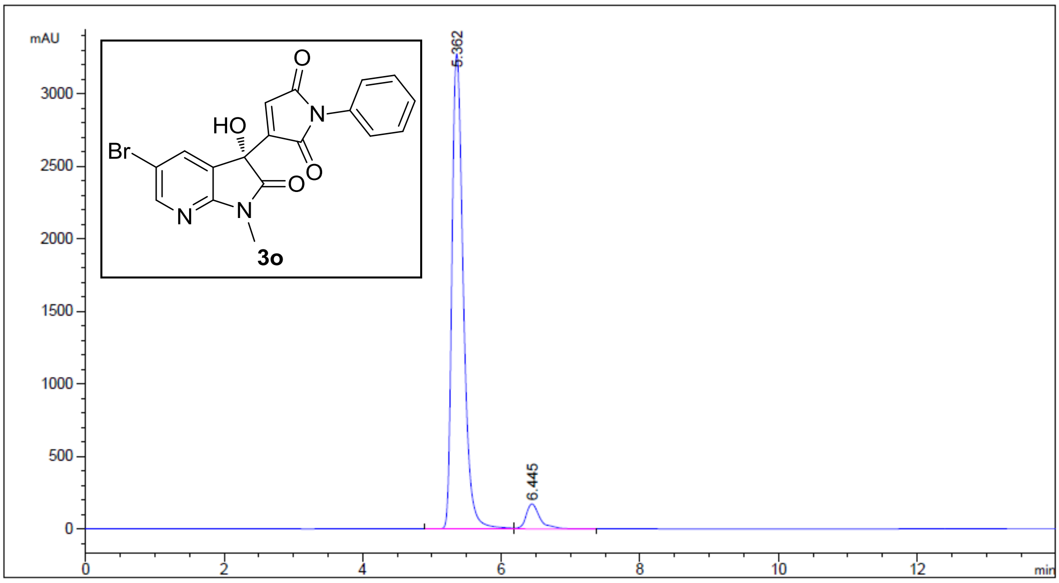

Peak #	RetTime [min]	Type	Width [min]	Area mAU *s	Height [mAU]	Area %
1	5.362	VV	0.1804	3.73162e4	3253.93433	93.9258
2	6.445	VB	0.2106	2413.22998	171.63893	6.0742

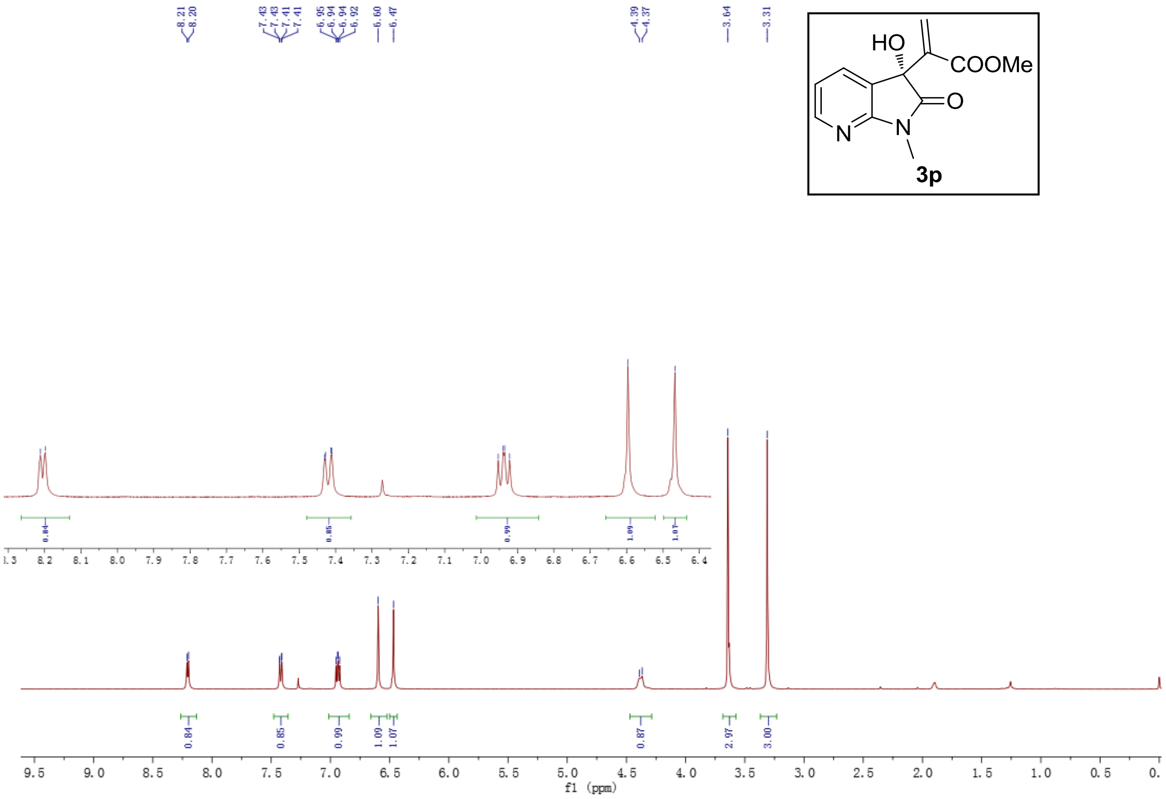
8.21
8.20
7.43
7.43
7.41
7.41
6.95
6.94
6.94
6.92
6.60
6.47
4.39
4.37
3.64
3.31
HO
COOMe
O
N
N
3p
0.84
0.85
0.99
1.09
1.07
0.87
2.97
3.00
f1 (ppm)

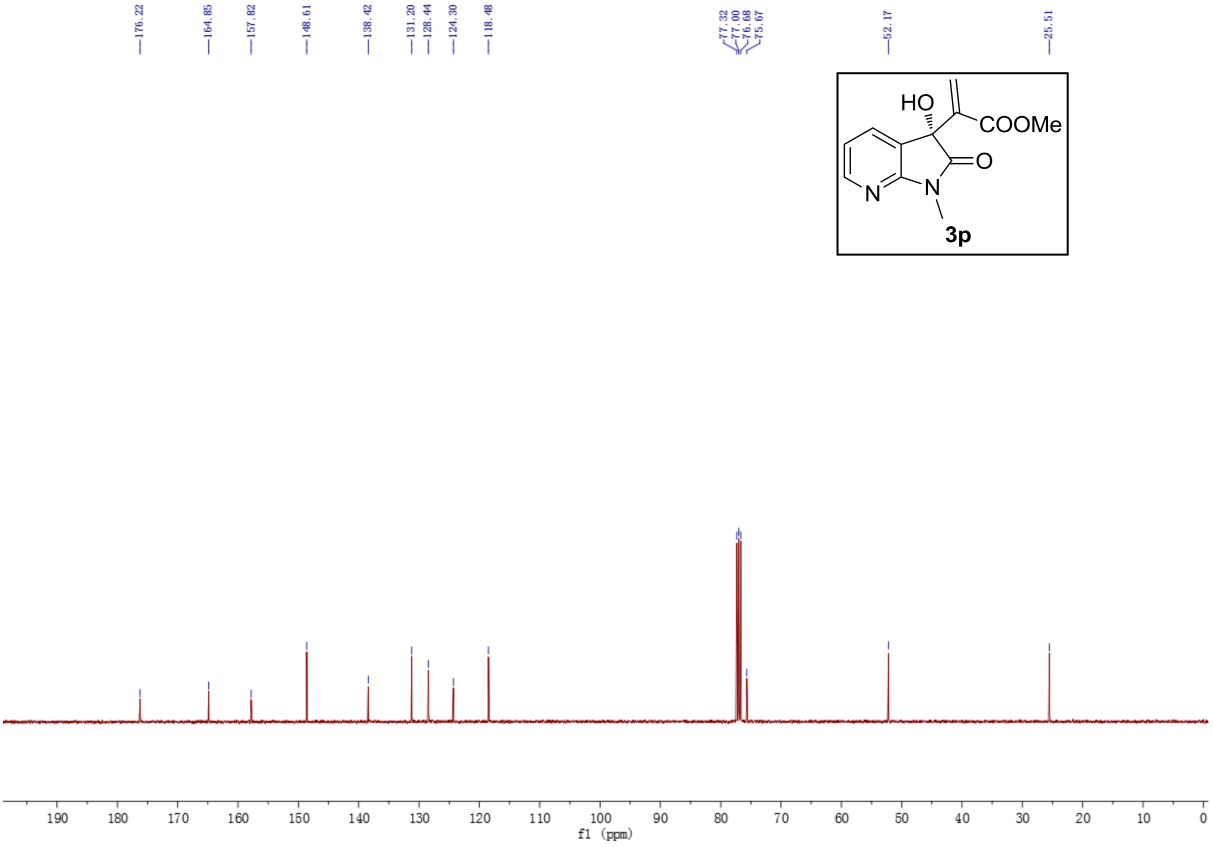
176.22
164.85
157.82
148.61
138.42
131.20
128.44
124.30
118.48
77.32
77.00
76.68
75.67
52.17
25.51
HO
COOMe
O
N
N
3p
f1 (ppm)

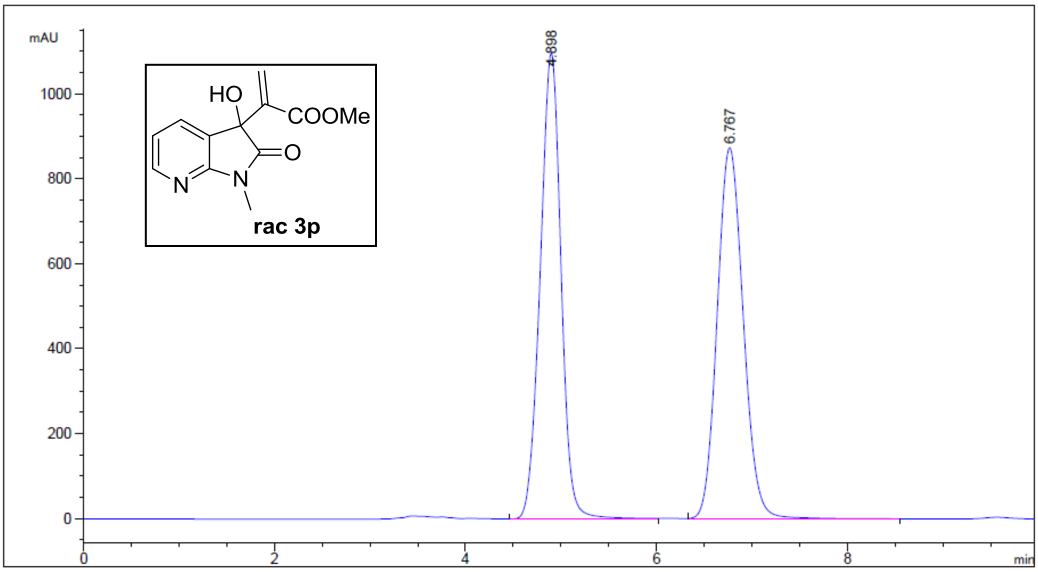

Peak #	RetTime [min]	Type	Width [min]	Area mAU *s	Height [mAU]	Area %
1	4.898	VB	0.2354	1.66453e4	1096.21497	49.9500
2	6.767	BB	0.2984	1.66786e4	873.77759	50.0500

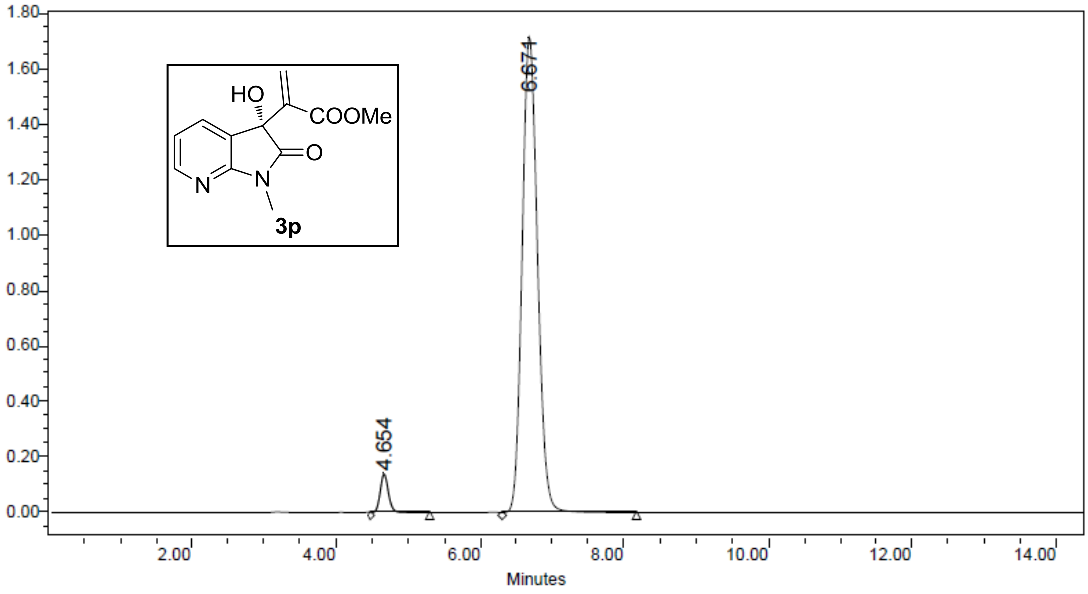

	RT (min)	Area (*sec)	% Area	Height ( )	% Height
1	4.654	1114311	4.19	139172	7.49
2	6.671	25475615	95.81	1719491	92.51

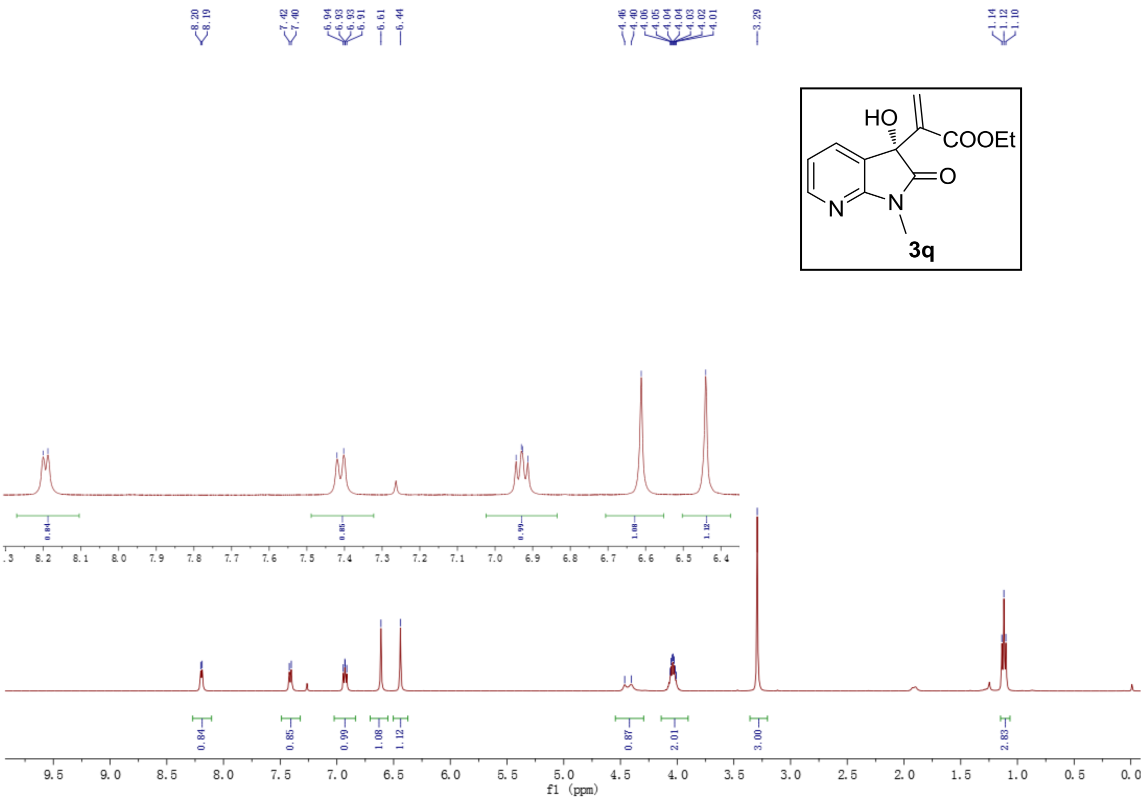
8.20
8.19
7.42
7.40
6.94
6.93
6.93
6.91
6.61
6.44
4.46
4.40
4.06
4.05
4.04
4.04
4.03
4.02
4.01
3.29
1.14
1.12
1.10
HO
COOEt
O
N
N
3q
0.84
0.85
0.99
1.08
1.12
0.87
2.01
3.00
2.83
f1 (ppm)

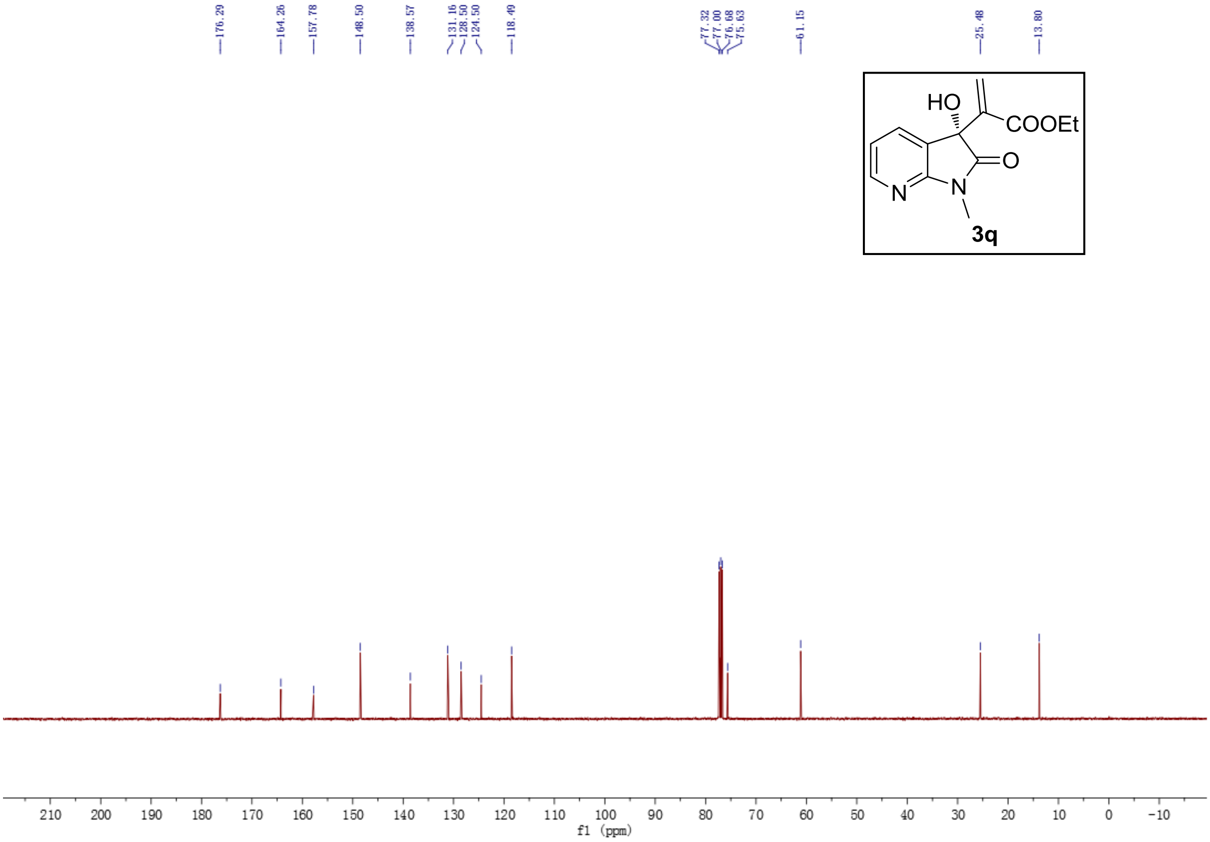
176.29
164.26
157.78
148.50
138.57
131.16
128.50
124.50
118.49
77.32
77.00
76.68
75.63
61.15
25.48
13.80
HO
COOEt
O
N
N
3q
f1 (ppm)

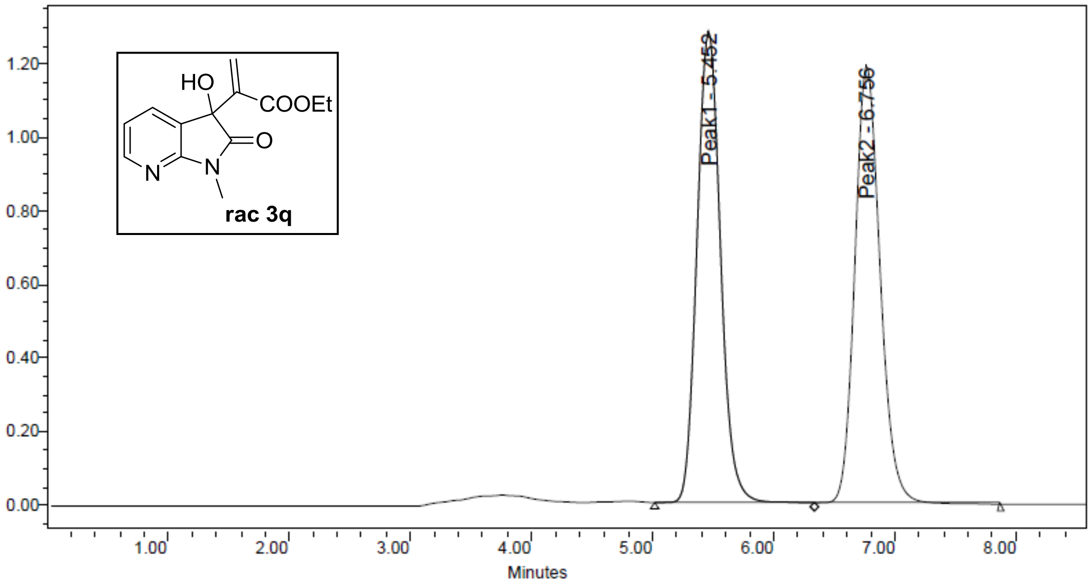

	Peak Name	RT (min)	Area (*sec)	% Area	Height ( )	% Height
1	Peak1	5.452	17295118	49.99	1281684	51.81
2	Peak2	6.756	17301318	50.01	1191986	48.19

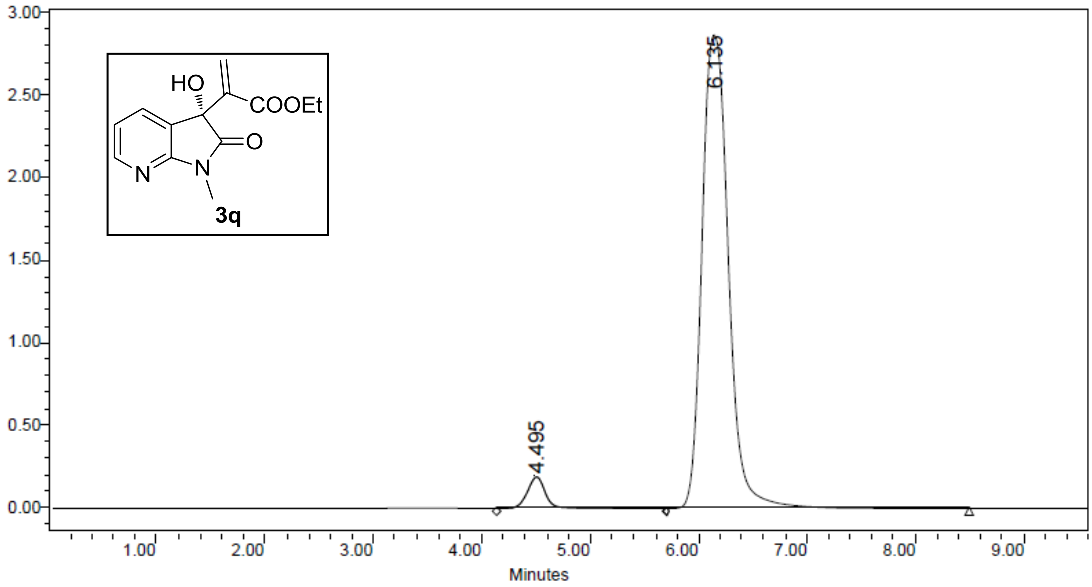

	RT (min)	Area (*sec)	% Area	Height ( )	% Height
1	4.495	2235840	4.40	191649	6.26
2	6.135	48609769	95.60	2871950	93.74

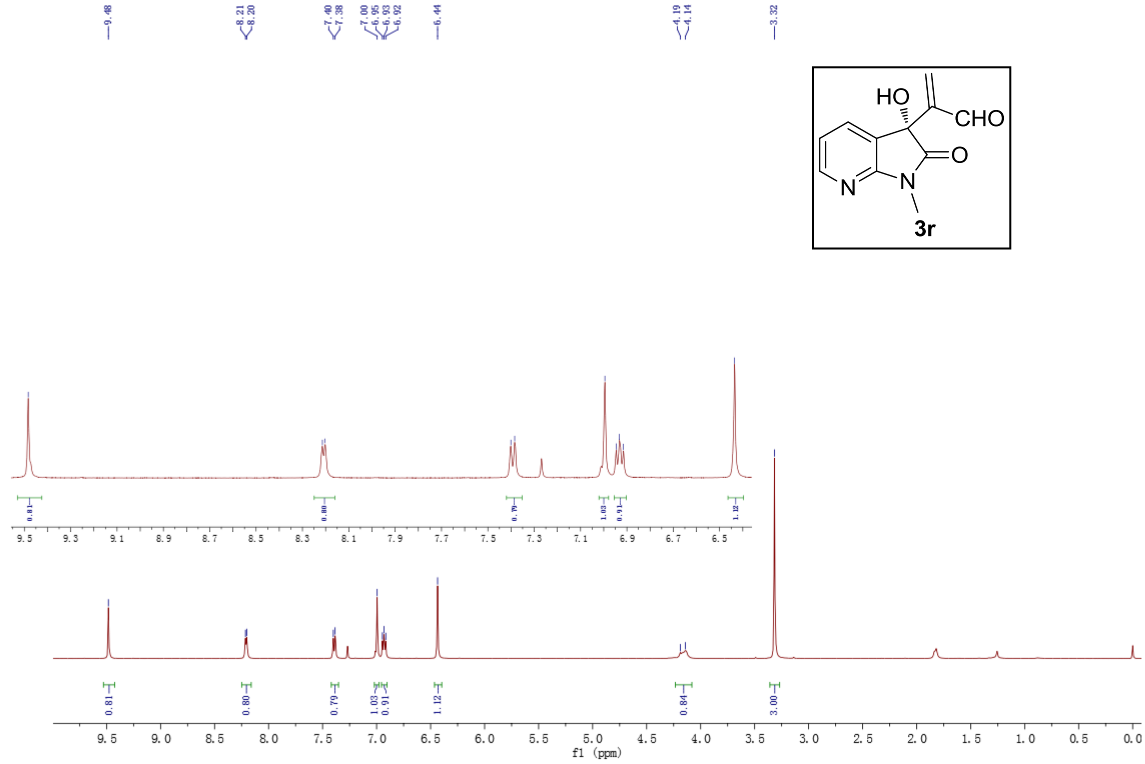
9.48
8.21
8.20
7.40
7.38
7.00
6.95
6.93
6.92
6.44
4.19
4.14
3.32
HO
CHO
O
N
N
3r
0.81
0.80
0.79
1.03
0.91
1.12
0.84
3.00
f1 (ppm)

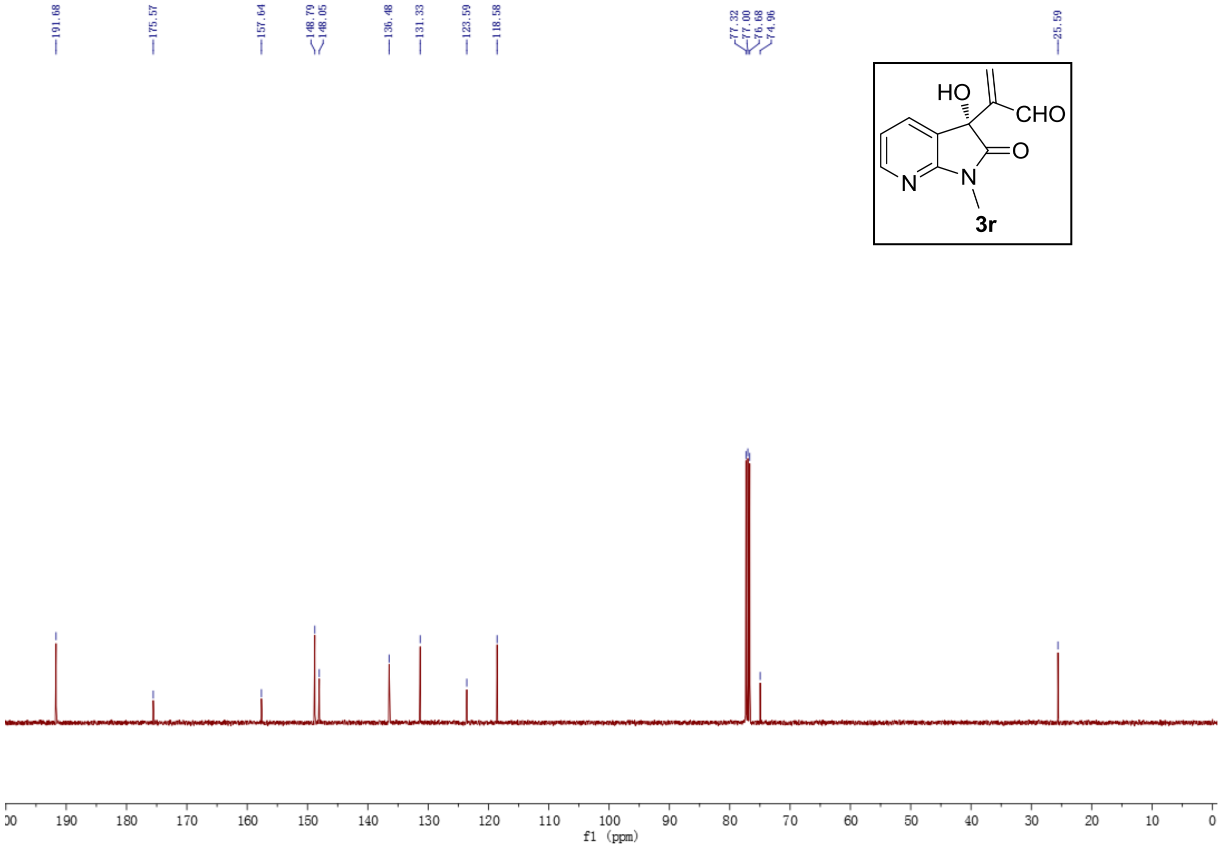
191.68
175.57
157.64
148.79
148.05
136.48
131.33
123.59
118.58
77.32
77.00
76.68
74.96
25.59
HO
CHO
O
N
N
3r
f1 (ppm)

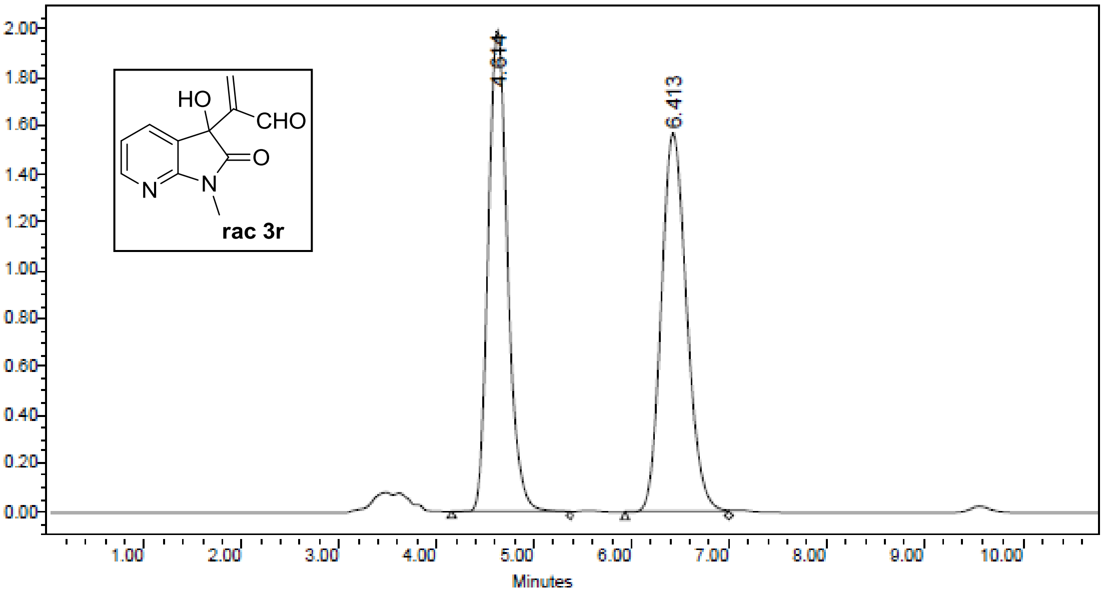

	RT (min)	Area (*sec)	% Area	Height ( )	% Height
1	4.614	27750191	49.22	1996050	55.94
2	6.413	28631347	50.78	1572143	44.06

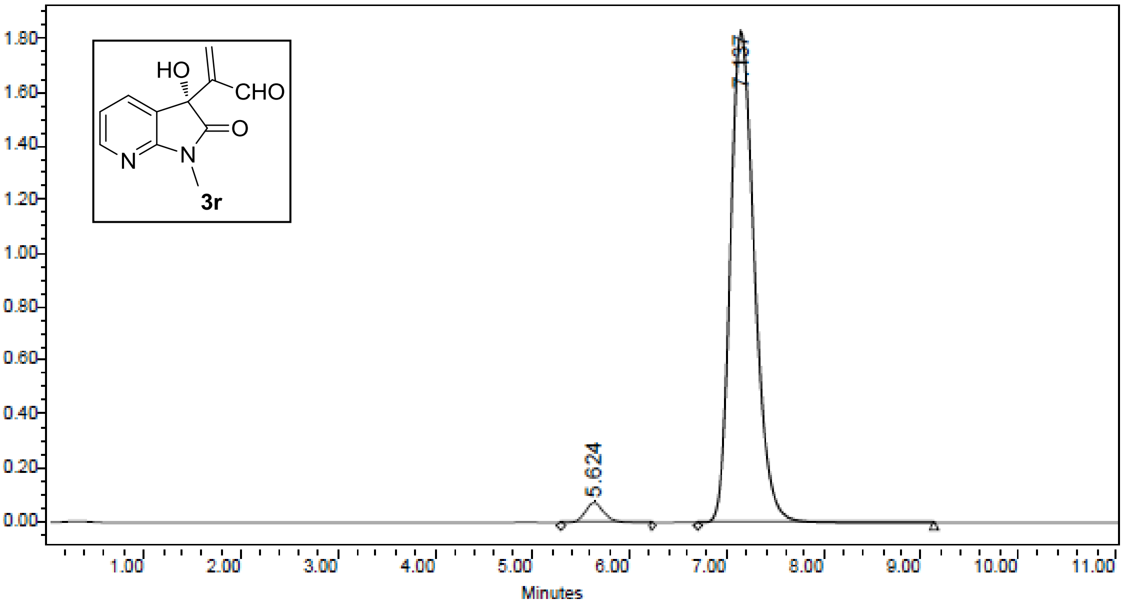

	RT (min)	Area (*sec)	% Area	Height ( )	% Height
1	5.624	1026751	3.15	74290	3.89
2	7.137	31595220	96.85	1833210	96.11
